# The Concise Guide to PHARMACOLOGY 2015/16: Enzymes

**DOI:** 10.1111/bph.13354

**Published:** 2015-12-09

**Authors:** Stephen PH Alexander, Doriano Fabbro, Eamonn Kelly, Neil Marrion, John A Peters, Helen E Benson, Elena Faccenda, Adam J Pawson, Joanna L Sharman, Christopher Southan, Jamie A Davies, CGTP Collaborators

**Affiliations:** ^1^ School of Biomedical Sciences University of Nottingham Medical School Nottingham NG7 2UH UK; ^2^ PIQUR Therapeutics Basel 4057 Switzerland; ^3^ School of Physiology and Pharmacology University of Bristol Bristol BS8 1TD UK; ^4^ Neuroscience Division, Medical Education Institute, Ninewells Hospital and Medical School University of Dundee Dundee DD1 9SY UK; ^5^ Centre for Integrative Physiology University of Edinburgh Edinburgh EH8 9XD UK

## Abstract

The Concise Guide to PHARMACOLOGY 2015/16 provides concise overviews of the key properties of over 1750 human drug targets with their pharmacology, plus links to an open access knowledgebase of drug targets and their ligands (www.guidetopharmacology.org), which provides more detailed views of target and ligand properties. The full contents can be found at http://onlinelibrary.wiley.com/doi/10.1111/bph.13354/full. G protein‐coupled receptors are one of the eight major pharmacological targets into which the Guide is divided, with the others being: G protein‐coupled receptors, ligand‐gated ion channels, voltage‐gated ion channels, other ion channels, nuclear hormone receptors, catalytic receptors and transporters. These are presented with nomenclature guidance and summary information on the best available pharmacological tools, alongside key references and suggestions for further reading. The Concise Guide is published in landscape format in order to facilitate comparison of related targets. It is a condensed version of material contemporary to late 2015, which is presented in greater detail and constantly updated on the website www.guidetopharmacology.org, superseding data presented in the previous Guides to Receptors & Channels and the Concise Guide to PHARMACOLOGY 2013/14. It is produced in conjunction with NC‐IUPHAR and provides the official IUPHAR classification and nomenclature for human drug targets, where appropriate. It consolidates information previously curated and displayed separately in IUPHAR‐DB and GRAC and provides a permanent, citable, point‐in‐time record that will survive database updates.

## Conflict of interest

The authors state that there are no conflicts of interest to declare.

## Overview

Enzymes are protein catalysts facilitating the conversion of substrates into products. The Nomenclature Committee of the International Union of Biochemistry and Molecular Biology (NC‐IUBMB) classifies enzymes into families, using a four number code, on the basis of the reactions they catalyse. There are six main families:

EC 1.‐.‐.‐ Oxidoreductases;

EC 2.‐.‐.‐ Transferases;

EC 3.‐.‐.‐ Hydrolases;

EC 4.‐.‐.‐ Lyases;

EC 5.‐.‐.‐ Isomerases;

EC 6.‐.‐.‐ Ligases.

Although there are many more enzymes than receptors in biology, and many drugs that target prokaryotic enzymes are effective medicines, overall the number of enzyme drug targets is relatively small [367, 401], which is not to say that they are of modest importance.

The majority of drugs which act on enzymes act as inhibitors; one exception is metformin, which appears to stimulate activity of AMP‐activated protein kinase, albeit through an imprecisely‐defined mechanism. Kinetic assays allow discrimination of competitive, non‐competitive, and un‐competitive inhibitors. The majority of inhibitors are competitive (acting at the enzyme's ligand recognition site), non‐competitive (acting at a distinct site; potentially interfering with co‐factor or co‐enzyme binding) or of mixed type. One rare example of an uncompetitive inhibitor is lithium ions, which are effective inhibitors at inositol monophosphatase only in the presence of high substrate concentrations. Some inhibitors are irreversible, including a group known as suicide substrates, which bind to the ligand recognition site and then couple covalently to the enzyme. It is beyond the scope of the Guide to give mechanistic information about the inhibitors described, although generally this information is available from the indicated literature.

Many enzymes require additional entities for functional activity. Some of these are used in the catalytic steps, while others promote a particular conformational change. Co‐factors are tightly bound to the enzyme and include metal ions and heme groups. Co‐enzymes are typically small molecules which accept or donate functional groups to assist in the enzymatic reaction. Examples include ATP, NAD, NADP and S‐adenosylmethionine, as well as a number of vitamins, such as riboflavin (vitamin B1) and thiamine (vitamin B2). Where co‐factors/co‐enzymes have been identified, the Guide indicates their involvement.

## Family structure

This is a complete listing of enzyme families included in the online IUPHAR/BPS Guide to PHARMACOLOGY database. Summary information is provided for a subset of enzyme families (those with page numbers) in the tables below. Family members judged to be of significant pharmacological interest have been included, with further enzymes listed in the database.

6028 Protein Kinases (EC 2.7.x.x)

‐ AGC: Containing PKA, PKC, PKG families

‐ DMPK family

‐ GEK subfamily

‐ Other DMPK family kinases

6028 Rho kinase

‐ G protein‐coupled receptor kinases

‐ BARK/GRK2 subfamily

‐ GRK1/3 subfamily

‐ MAST family

‐ NDR family

‐ PDK1 family

‐ Protein kinase A

‐ Akt (Protein kinase B)

6029 Protein kinase C (PKC)

6029 Alpha subfamily

6029 Delta subfamily

6030 Eta subfamily

‐ Iota subfamily

‐ Protein kinase G (PKG)

‐ Protein kinase N (PKN) family

‐ RSK family

‐ MSK subfamily

‐ p70 subfamily

‐ RSK subfamily

‐ RSKR subfamily

‐ RSKL family

‐ SGK family

‐ YANK family

‐ Atypical

‐ ABC1 family

‐ ABC1‐A subfamily

‐ ABC1‐B subfamily

‐ Alpha kinase family

‐ ChaK subfamily

‐ eEF2K subfamily

‐ Other alpha kinase family kinases

‐ BCR family

‐ Bromodomain kinase (BRDK) family

‐ G11 family

‐ Phosphatidyl inositol 3' kinase‐related kinases (PIKK) family

‐ ATR subfamily

6030 FRAP subfamily

‐ SMG1 subfamily

‐ TRRAP subfamily

‐ Other PIKK family kinases

‐ RIO family

‐ RIO1 subfamily

‐ RIO2 subfamily

‐ RIO3 subfamily

‐ PDHK family

‐ Pyruvate dehydrogenase kinase (PDHK) family

‐ TAF1 family

‐ TIF1 family

‐ CAMK: Calcium/calmodulin‐dependent protein kinases

‐ CAMK‐like (CAMKL) family

‐ AMPK subfamily

‐ BRSK subfamily

‐ CHK1 subfamily

‐ HUNK subfamily

‐ LKB subfamily

‐ MARK subfamily

‐ MELK subfamily

‐ NIM1 subfamily

‐ NuaK subfamily

‐ PASK subfamily

‐ QIK subfamily

‐ SNRK subfamily

‐ CAMK‐unique family

‐ CASK family

‐ DCAMKL family

‐ Death‐associated kinase (DAPK) family

‐ MAPK‐Activated Protein Kinase (MAPKAPK) family

‐ MAPKAPK subfamily

‐ MKN subfamily

‐ Myosin Light Chain Kinase (MLCK) family

‐ Phosphorylase kinase (PHK) family

‐ PIM family

‐ Protein kinase D (PKD) family

‐ PSK family

‐ RAD53 family

‐ Trbl family

‐ Trio family

‐ CK1: Casein kinase 1

‐ Casein kinase 1 (CK1) family

‐ Tau tubulin kinase (TTBK) family

‐ Vaccina related kinase (VRK) family

‐ CMGC: Containing CDK, MAPK, GSK3, CLK families

‐ CLK family

‐ Cyclin‐dependent kinase (CDK) family

‐ CCRK subfamily

‐ CDK1 subfamily

6031 CDK4 subfamily

‐ CDK5 subfamily

‐ CDK7 subfamily

‐ CDK8 subfamily

‐ CDK9 subfamily

‐ CDK10 subfamily

‐ CRK7 subfamily

‐ PITSLRE subfamily

‐ TAIRE subfamily

‐ Cyclin‐dependent kinase‐like (CDKL) family

‐ Dual‐specificity tyrosine‐(Y)‐phosphorylation regulated kinase (DYRK) family

‐ Dyrk1 subfamily

‐ Dyrk2 subfamily

‐ HIPK subfamily

‐ PRP4 subfamily

‐ Glycogen synthase kinase (GSK) family

6031 GSK subfamily

‐ Mitogen‐activated protein kinases (MAP kinases)

‐ ERK subfamily

‐ Erk7 subfamily

‐ JNK subfamily

‐ p38 subfamily

‐ nmo subfamily

‐ RCK family

‐ SRPK family

‐ Other protein kinases

‐ CAMKK family

‐ Meta subfamily

‐ Aurora kinase (Aur) family

‐ Bub family

‐ Bud32 family

‐ Casein kinase 2 (CK2) family

‐ CDC7 family

‐ Haspin family

‐ IKK family

‐ IRE family

‐ MOS family

‐ NAK family

‐ NIMA (never in mitosis gene a)‐ related kinase (NEK) family

‐ NKF1 family

‐ NKF2 family

‐ NKF4 family

‐ NKF5 family

‐ NRBP family

‐ Numb‐associated kinase (NAK) family

‐ Other‐unique family

6032 Polo‐like kinase (PLK) family

‐ PEK family

‐ GCN2 subfamily

‐ PEK subfamily

‐ Other PEK family kinases

‐ SgK493 family

‐ Slob family

‐ TBCK family

‐ TOPK family

‐ Tousled‐like kinase (TLK) family

‐ TTK family

‐ Unc‐51‐like kinase (ULK) family

‐ VPS15 family

‐ WEE family

‐ Wnk family

‐ Miscellaneous protein kinases

‐ actin‐binding proteins ADF family

‐ Twinfilin subfamily

‐ SCY1 family

‐ Hexokinases

‐ STE: Homologs of yeast Sterile 7, Sterile 11, Sterile 20 kinases

6032 STE7 family

‐ STE11 family

‐ STE20 family

‐ FRAY subfamily

‐ MSN subfamily

‐ NinaC subfamily

‐ PAKA subfamily

‐ PAKB subfamily

‐ STLK subfamily

‐ TAO subfamily

‐ STE20 family

‐ STE‐unique family

‐ TK: Tyrosine kinase

6033 Abl family

6033 Ack family

‐ Csk family

‐ Fak family

‐ Fer family

6034 Janus kinase (JakA) family

6034 Src family

‐ Syk family

6035 Tec family

‐ TKL: Tyrosine kinase‐like

‐ Interleukin‐1 receptor‐associated kinase (IRAK) family

‐ Leucine‐rich repeat kinase (LRRK) family

‐ LIM domain kinase (LISK) family

‐ LIMK subfamily

‐ TESK subfamily

‐ Mixed Lineage Kinase (MLK) family

‐ HH498 subfamily

‐ ILK subfamily

‐ LZK subfamily

‐ MLK subfamily

‐ TAK1 subfamily

6035 RAF family

‐ Receptor interacting protein kinase (RIPK) family

‐ TKL‐unique family

6036 Peptidases and proteinases

‐ AA: Aspartic (A) Peptidases

6036 A1: Pepsin

‐ AD: Aspartic (A) Peptidases

6037 A22: Presenilin

‐ CA: Cysteine (C) Peptidases

‐ C1: Papain

‐ C2: Calpain

‐ C12: Ubiquitin C‐terminal hydrolase

‐ C19: Ubiquitin‐specific protease

‐ C54: Aut2 peptidase

‐ CD: Cysteine (C) Peptidases

‐ C13: Legumain

6037 C14: Caspase

‐ CE: Cysteine (C) Peptidases

‐ C48: Ulp1 endopeptidase

‐ M‐: Metallo (M) Peptidases

‐ M79: Prenyl protease 2

‐ MA: Metallo (M) Peptidases

6037 M1: Aminopeptidase N

6038 M2: Angiotensin‐converting (ACE and ACE2)

6038 M10: Matrix metallopeptidase

6039 M12: Astacin/Adamalysin

‐ M13: Neprilysin

‐ M49: Dipeptidyl‐peptidase III

‐ MC: Metallo (M) Peptidases

‐ M14: Carboxypeptidase A

‐ ME: Metallo (M) Peptidases

‐ M16: Pitrilysin

‐ MF: Metallo (M) Peptidases

‐ M17: Leucyl aminopeptidase

‐ MG: Metallo (M) Peptidases

‐ M24: Methionyl aminopeptidase

‐ MH: Metallo (M) Peptidases

‐ M18: Aminopeptidase I

‐ M20: Carnosine dipeptidase

6039 M28: Aminopeptidase Y

‐ MJ: Metallo (M) Peptidases

6040 M19: Membrane dipeptidase

‐ MP: Metallo (M) Peptidases

‐ M67: PSMD14 peptidase

‐ PA: Serine (S) Peptidases

6040 S1: Chymotrypsin

‐ PB: Threonine (T) Peptidases

6041 T1: Proteasome

‐ T2: Glycosylasparaginase precursor

‐ PC: Cysteine (C) Peptidases

‐ C26: Gamma‐glutamyl hydrolase

‐ SB: Serine (S) Peptidases

6042 S8: Subtilisin

‐ SC: Serine (S) Peptidases

6042 S9: Prolyl oligopeptidase

‐ S10: Carboxypeptidase Y

‐ S28: Lysosomal Pro‐Xaa carboxypeptidase

‐ S33: prolyl aminopeptidase

6042 Acetylcholine turnover

6044 Adenosine turnover

6045 Amino acid hydroxylases

6046 L‐Arginine turnover

6047 Arginase

6047 Arginine:glycine amidinotransferase

6047 Dimethylarginine dimethylaminohydrolases

6048 Nitric oxide synthases

6048 Carboxylases and decarboxylases

6049 Carboxylases

6050 Decarboxylases

6052 Catecholamine turnover

6055 Ceramide turnover

6055 Serine palmitoyltransferase

‐ 3‐ketodihydrosphingosine reductase

6056 Ceramide synthase

6057 Sphingolipid *Δ* ^14^‐desaturase

6058 Sphingomyelin synthase

6058 Sphingomyelin phosphodiesterase

6059 Neutral sphingomyelinase coupling factors

6059 Ceramide glucosyltransferase

6060 Acid ceramidase

6060 Neutral ceramidases

6061 Alkaline ceramidases

6061 Ceramide kinase

6062 Chromatin modifying enzymes

‐ Enzymatic bromodomain‐containing proteins

‐ Bromodomain kinase (BRDK) family

‐ TAF1 family

‐ TIF1 family

‐ 1.14.11.‐ Histone demethylases

‐ 2.1.1.43 Histone methyltransferases (HMTs)

‐ 2.3.1.48 Histone acetyltransferases (HATs)

‐ 3.6.1.3 ATPases

6062 2.1.1.‐ Protein arginine N‐methyltransferases

6062 3.5.1.‐ Histone deacetylases (HDACs)

6063 Cyclic nucleotide turnover

6063 Adenylyl cyclases

6064 Soluble guanylyl cyclase

6065 Exchange protein activated by cyclic AMP (Epac)

6066 Phosphodiesterases, 3',5'‐cyclic nucleotide

6069 Cytochrome P450

6069 CYP1 family

6070 CYP2 family

6070 CYP3 family

6071 CYP4 family

6072 CYP5, CYP7 and CYP8 families

6072 CYP11, CYP17, CYP19, CYP20 and CYP21 families

6073 CYP24, CYP26 and CYP27 families

6074 CYP39, CYP46 and CYP51 families

6076 Eicosanoid turnover

6075 Endocannabinoid turnover

6077 Cyclooxygenase

6077 Prostaglandin synthases

6079 Lipoxygenases

6080 Leukotriene and lipoxin metabolism

6081 GABA turnover

6082 Glycerophospholipid turnover

‐ Lipid modifying kinases

6083 1‐phosphatidylinositol 4‐kinase family

6083 Phosphatidylinositol‐4‐phosphate 3‐kinase family

6084 Phosphatidylinositol 3‐kinase family

6084 Phosphatidylinositol‐4,5‐bisphosphate 3‐kinase family

6085 1‐phosphatidylinositol‐3‐phosphate 5‐kinase family

6085 Type I PIP kinases (1‐phosphatidylinositol‐4‐phosphate 5‐kinase family)

6086 Type II PIP kinases (1‐phosphatidylinositol‐5‐phosphate 4‐kinase family)

6100 Sphingosine kinase

6087 Phosphoinositide‐specific phospholipase C

6088 Phospholipase A_2_

6089 Phosphatidylcholine‐specific phospholipase D

6090 Lipid phosphate phosphatases

6082 Phosphatidylinositol kinases

6091 Haem oxygenase

6092 Hydrogen sulphide synthesis

6093 Hydrolases

6093 Inositol phosphate turnover

6094 Inositol 1,4,5‐trisphosphate 3‐kinases

6094 Inositol polyphosphate phosphatases

6094 Inositol monophosphatase

6095 Lanosterol biosynthesis pathway

6097 Nucleoside synthesis and metabolism

6099 Sphingosine 1‐phosphate turnover

6100 Sphingosine kinase

6100 Sphingosine 1‐phosphate phosphatase

6101 Sphingosine 1‐phosphate lyase

6101 Thyroid hormone turnover

‐ 1.4.3.13 Lysyl oxidases

‐ 1.13.11.‐ Dioxygenases

6103 1.14.11.29 2‐oxoglutarate oxygenases transferases

‐ 2.3.‐.‐ Acyltransferases

6103 2.4.2.30 poly(ADP‐ribose)polymerases

6104 2.5.1.58 Protein farnesyltransferase

‐ 3.1.‐.‐ Ester bond enzymes

‐ 3.1.1.‐ Carboxylic Ester Hydrolases

‐ 3.2.1.‐ Glycosidases

‐ 3.4.21.46 Complement factor D

6062 3.5.1.‐ Histone deacetylases (HDACs)

6104 3.5.3.15 Peptidyl arginine deiminases (PADI)

‐ 3.6.5.2 Small monomeric GTPases

6104 RAS subfamily

6105 4.2.1.1 Carbonate dehydratases

‐ 5.‐.‐.‐ Isomerases

‐ 5.2.‐.‐ Cis‐trans‐isomerases

6105 5.99.1.2 DNA Topoisomerases

## Protein Kinases (EC 2.7.x.x)

### Overview

Protein kinases (E.C. 2.7.11.‐) use the co‐substrate ATP to phosphorylate serine and/or threonine residues on target proteins. Analysis of the human genome suggests the presence of 518 protein kinases in man, with over 100 protein kinase‐like pseudogenes [313]. It is beyond the scope of the Concise Guide to list all these protein kinase activities; the full listing may be seen at www.GuidetoPHARMACOLOGY.org.

Most inhibitors of these enzymes have been assessed in cell‐free investigations and so may appear to ‘lose’ potency and selectivity in intact cell assays. In particular, ambient ATP concentrations may be influential in responses to inhibitors, since the majority are directed at the ATP binding site [103].

### Further Reading

Eglen R *et al*. (2011) Drug discovery and the human kinome: recent trends. *Pharmacol. Ther.* **130**: 144‐56 [PMID:21256157]

Graves LM *et al*. (2013) The dynamic nature of the kinome. *Biochem. J.* **450**: 1‐8 [PMID:23343193]

Liu Q *et al*. (2013) Developing irreversible inhibitors of the protein kinase cysteinome. *Chem. Biol.* **20**: 146‐59 [PMID:23438744]

Martin KJ *et al*. (2012) Selective kinase inhibitors as tools for neuroscience research. *Neuropharmacology* **63**: 1227‐37 [PMID:22846224]

Tarrant MK *et al*. (2009) The chemical biology of protein phosphorylation. *Annu. Rev. Biochem.* **78**: 797‐825 [PMID:19489734]

Wu‐Zhang AX *et al*. (2013) Protein kinase C pharmacology: refining the toolbox. *Biochem. J.* **452**: 195‐209 [PMID:23662807]

## Rho kinase

### Overview

Rho kinase (also known as P160ROCK, Rho‐activated kinase) is activated by members of the Rho small G protein family, which are activated by GTP exchange factors, such as *ARHGEF1*(Q92888, p115‐RhoGEF), which in turn may be activated by G*α*_12/13_ subunits [269].

**Table:** 

Nomenclature	Rho‐associated, coiled‐coil containing protein kinase 1	Rho‐associated, coiled‐coil containing protein kinase 2
Systematic nomenclature	ROCK1	ROCK2
Common abreviation	Rho kinase 1	Rho kinase 2
HGNC, UniProt	*ROCK1*, Q13464	*ROCK2*, O75116
EC number	2.7.11.1	2.7.11.1
Inhibitors	RKI‐1447 (pIC_50_>9) [387], Y27632 (pIC_50_ 7.3) [529], fasudil (p*K*_i_ 7) [403], Y27632 (p*K*_i_ 6.8) [496], fasudil (pIC_50_ 5.5) [403]	RKI‐1447 (pIC_50_>9) [387], compound 11d [DOI: 10.1039/c0md00194e] (pIC_50_>9) [77], GSK269962A (pIC_50_ 8.4) [118], compound 32 [PMID: 20471253] (pIC_50_ 8.4) [45], compound 22 [PMID: 20462760] (pIC_50_ 7.7) [529], Y27632 (pIC_50_ 7.2) [529], Y27632 (p*K*_i_ 6.8) [496], fasudil (pIC_50_ 5.9) [403]
Selective inhibitors	GSK269962A (pIC_50_ 8.8) [118]	–

## Protein kinase C (PKC)

### Overview

Protein kinase C is the target for the tumour‐promoting phorbol esters, such as tetradecanoyl‐*β*‐phorbol acetate (TPA, also known as phorbol 12‐myristate 13‐acetate).

*Classical protein kinase C isoforms:* **PKC*α*, PKC*β*, PKC*γ***. Members of the classical protein kinase C family are activated by Ca^2+^ and diacylglycerol, and may be inhibited by GF109203X, calphostin C, GÖ 6983, chelerythrine and Ro31‐8220.

*Novel protein kinase C isoforms:* **PKC*δ*, PKC*ε*, PKC*η*, PKC*θ* and PKC*μ***. Members of the classical protein kinase C family are activated by diacylglycerol and may be inhibited by calphostin C, GÖ 6983 and chelerythrine.

*Atypical protein kinase C isoforms:* **PKC*ι*, PKC*ζ***.

## Alpha subfamily

**Table:** 

Nomenclature	protein kinase C, beta	protein kinase C, gamma
Common abreviation	PKC*β*	PKC*γ*
HGNC, UniProt	*PRKCB*, P05771	*PRKCG*, P05129
EC number	2.7.11.13	2.7.11.13
Inhibitors	sotrastaurin (pIC_50_ 8.7) [506], GÖ 6983 (pIC_50_ 8.1) [183], GF109203X (pIC_50_ 7.8) [490] – Bovine, 7‐hydroxystaurosporine (pIC_50_ 7.5) [431]	GÖ 6983 (pIC_50_ 8.2) [183], 7‐hydroxystaurosporine (pIC_50_ 7.5) [431]
Selective inhibitors	ruboxistaurin (pIC_50_ 8.2) [238], enzastaurin (pIC_50_ 7.5) [132], CGP53353 (pIC_50_ 6.4) [70]	–

## Delta subfamily

**Table:** 

Nomenclature	protein kinase C, alpha	protein kinase C, delta
Common abreviation	PKC*α*	PKC*δ*
HGNC, UniProt	*PRKCA*, P17252	*PRKCD*, Q05655
EC number	2.7.11.13	2.7.11.13
Activators	–	ingenol mebutate (p*K*_i_ 9.4) [252]
Inhibitors	sotrastaurin (pIC_50_ 8.7) [506], GÖ 6983 (pIC_50_ 8.1) [183], 7‐hydroxystaurosporine (pIC_50_ 7.5) [431]	sotrastaurin (pIC_50_ 8.9) [506], GÖ 6983 (pIC_50_ 8) [183]

## Eta subfamily

**Table:** 

Nomenclature	protein kinase C, epsilon
Common abreviation	PKC*ε*
HGNC, UniProt	*PRKCE*, Q02156
EC number	2.7.11.13
Inhibitors	sotrastaurin (pIC_50_ 8.2) [506]

## FRAP subfamily

**Table:** 

Nomenclature	mechanistic target of rapamycin (serine/threonine kinase)
Common abreviation	mTOR
HGNC, UniProt	*MTOR*, P42345
EC number	2.7.11.1
Inhibitors	ridaforolimus (pIC_50_ 9.7) [408], torin 1 (pIC_50_ 9.5) [291], INK‐128 (pIC_50_ 9) [219], INK‐128 (p*K*_i_ 8.9) [219], gedatolisib (pIC_50_ 8.8) [500], dactolisib (pIC_50_ 8.2) [310], PP‐242 (pIC_50_ 8.1) [14], PP121 (pIC_50_ 8) [14], XL388 (pIC_50_ 8) [472], PF‐04691502 (p*K*_i_ 7.8) [290], apitolisib (p*K*_i_ 7.8) [467]
Selective inhibitors	everolimus (pIC_50_ 8.7) [427], temsirolimus (pIC_50_ 5.8) [266]

## CDK4 subfamily

**Table:** 

Nomenclature	cyclin‐dependent kinase 4	cyclin‐dependent kinase 6
Common abreviation	CDK4	CDK6
HGNC, UniProt	*CDK4*, P11802	*CDK6*, Q00534
EC number	2.7.11.22	2.7.11.22
Inhibitors	R547 (p*K*_i_ 9) [107], palbociclib (pIC_50_ 8) [151], Ro‐0505124 (pIC_50_ 7.7) [115], riviciclib (pIC_50_ 7.2) [246], alvocidib (p*K*_i_ 7.2) [64]	palbociclib (pIC_50_ 7.8) [151]

## GSK subfamily

**Table:** 

Nomenclature	glycogen synthase kinase 3 beta
Common abreviation	GSK3B
HGNC, UniProt	*GSK3B*, P49841
EC number	2.7.11.26
Inhibitors	CHIR‐98014 (pIC_50_ 9.2) [407], LY2090314 (pIC_50_ 9) [125], CHIR‐99021 (pIC_50_ 8.2) [407], SB 216763 (pIC_50_∼8.1) [88], 1‐azakenpaullone (pIC_50_ 7.7) [272], SB‐415286 (pIC_50_∼7.4) [88], IM‐12 (pIC_50_ 7.3) [424]
Selective inhibitors	AZD2858 (p*K*_i_ 8.3) [29]
Comments	Due to its Tau phosphorylating activity, small molecule inhibitors of GSK‐3*β* are being investigated as potential treatments for Alzheimer's disease (AD) [29]. GSK‐3*β* also plays a role in canonical Wnt pathway signalling, the normal activity of which is crucial for the maintenance of normal bone mass. It is hypothesised that small molecule inhibitors of GSK‐3*β* may provide effective therapeutics for the treatment of diseases characterised by low bone mass [317].

## Polo‐like kinase (PLK) family

**Table:** 

Nomenclature	polo‐like kinase 4
Common abreviation	PLK4
HGNC, UniProt	*PLK4*, O00444
EC number	2.7.11.21
Inhibitors	CFI‐400945 (pIC_50_ 8.6) [320]

## STE7 family

**Table:** 

Nomenclature	mitogen‐activated protein kinase kinase 1	mitogen‐activated protein kinase kinase 2
Common abreviation	MEK1	MEK2
HGNC, UniProt	*MAP2K1*, Q02750	*MAP2K2*, P36507
EC number	2.7.12.2	2.7.12.2
Inhibitors	trametinib (pIC_50_ 9–9.1) [173, 536], PD 0325901 (pIC_50_ 8.1) [195], binimetinib (pIC_50_ 7.9) [382], refametinib (pIC_50_ 7.7) [229], CI‐1040 (p*K*_d_ 6.9) [105]	trametinib (pIC_50_ 8.7) [536], binimetinib (pIC_50_ 7.9) [382], refametinib (pIC_50_ 7.3) [229]

## Abl family

**Table:** 

Nomenclature	ABL proto‐oncogene 1, non‐receptor tyrosine kinase
Common abreviation	Abl
HGNC, UniProt	*ABL1*, P00519
EC number	2.7.10.2
Inhibitors	compound 8h (pIC_50_ 9.7) [487], dasatinib (pIC_50_ 9.6) [258], compound 24 (pIC_50_ 9.3) [110], PD‐173955 (p*K*_d_ 9.2) [105], bosutinib (pIC_50_ 9) [176], PD‐173955 (pIC_50_∼8.3) [346], bafetinib (pIC_50_ 7.6–8.2) [216, 257], ponatinib (pIC_50_ 8.1) [220], nilotinib (pIC_50_ 7.8) [356], PP121 (pIC_50_ 7.7) [14], imatinib (pIC_50_ 6.7) [216], GNF‐5 (pIC_50_ 6.7) [549]

## Ack family

**Table:** 

Nomenclature	tyrosine kinase, non‐receptor, 2
Common abreviation	Ack
HGNC, UniProt	*TNK2*, Q07912
EC number	2.7.10.2
Inhibitors	compound 30 (pIC_50_ 9) [114]

## anus kinase (JakA) family

**Table:** 

Nomenclature	Janus kinase 1	Janus kinase 2	Janus kinase 3	tyrosine kinase 2
Common abreviation	JAK1	JAK2	JAK3	Tyk2
HGNC, UniProt	*JAK1*, P23458	*JAK2*, O60674	*JAK3*, P52333	*TYK2*, P29597
EC number	2.7.10.2	2.7.10.2	2.7.10.2	2.7.10.2
Inhibitors	ruxolitinib (pIC_50_ 8.5–10.1) [191, 395], filgotinib (pIC_50_ 8) [497]	NS‐018 (pIC_50_ 9.1) [350], BMS‐911543 (pIC_50_ 9) [393], AT‐9283 (pIC_50_ 8.9) [218], XL019 (pIC_50_ 8.7) [143], fedratinib (pIC_50_ 8.5) [311, 521], gandotinib (pIC_50_ 8.4) [308]	AT‐9283 (pIC_50_ 9) [218]	–
Selective inhibitors	–	compound 1d (pIC_50_>9) [509]	–	–
Comments	–	The JAK2^V617F^ mutation, which causes constitutive activation, plays an oncogenic role in the pathogenesis of the myeloproliferative disorders, polycythemia vera, essential thrombocythemia, and idiopathic myelofibrosis [58, 109]. Small molecule compounds which inhibit aberrant JAK2 activity are being developed as novel anti‐cancer pharmaceuticals.	–	–

## Src family

**Table:** 

Nomenclature	BLK proto‐oncogene, Src	fyn‐related Src family	FYN proto‐oncogene, Src	LYN proto‐oncogene, Src	SRC proto‐oncogene,
	Src family tyrosine kinase	tyrosine kinase	family tyrosine kinase	family tyrosine kinase	non-receptor tyrosine kinase
Common abreviation	Blk	FRK	Fyn	Lyn	Src
HGNC, UniProt	*BLK*, P51451	*FRK*, P42685	*FYN*, P06241	*LYN*, P07948	*SRC*, P12931
EC number	2.7.10.2	2.7.10.2	2.7.10.2	2.7.10.2	2.7.10.2
Inhibitors	–	–	–	bafetinib (pIC_50_ 8) [216]	WH‐4‐023 (pIC_50_ 8.2) [318], PD166285 (p*K*_i_ 8.1) [371], PP121 (pIC_50_ 7.8) [14], ENMD‐2076 (pIC_50_ 7.7) [389]

## Tec family

**Table:** 

Nomenclature	BMX non‐receptor tyrosine kinase	Bruton agammaglobulinemia tyrosine kinase	TXK tyrosine kinase
Common abreviation	Etk	Btk	TXK
HGNC, UniProt	*BMX*, P51813	*BTK*, Q06187	*TXK*, P42681
EC number	2.7.10.2	2.7.10.2	2.7.10.2
Inhibitors	–	ibrutinib (pIC_50_ 9.3) [370], compound 31 [PMID: 24915291] (pIC_50_ 8.4) [285], compound 38 [PMID: 24915291] (pIC_50_>8.4) [285]	–
Selective inhibitors	–	CGI1746 (pIC_50_ 8.7) [112]	–

## RAF family

**Table:** 

Nomenclature	B‐Raf proto‐oncogene, serine/threonine kinase	Raf‐1 proto‐oncogene, serine/threonine kinase
Common abreviation	B‐Raf	c‐Raf
HGNC, UniProt	*BRAF*, P15056	*RAF1*, P04049
EC number	2.7.11.1	2.7.11.1
Inhibitors	GDC‐0879 (pIC_50_ 9.7–9.9) [105, 193], dabrafenib (pIC_50_ 8.5) [277], regorafenib (pIC_50_ 7.6) [545], vemurafenib (pIC_50_ 7) [510], PLX‐4720 (p*K*_d_ 6.5) [105], compound 2 [PMID: 26061392] (p*K*_d_ 6.3) [215], CHIR‐265 (p*K*_d_ 5.9) [105]	–
Selective inhibitors	–	GW5074 (pIC_50_ 8.1) [80]

## 25 Peptidases and proteinases

### Overview

Peptidases and proteinases hydrolyse peptide bonds, and can be simply divided on the basis of whether terminal peptide bonds are cleaved (exopeptidases and exoproteinases) at the amino terminus (aminopeptidases) or carboxy terminus (carboxypeptidases). Non‐terminal peptide bonds are cleaved by endopeptidases and endoproteinases, which are divided into serine endopeptidases (EC 3.4.21.‐), cysteine endopeptidases (EC 3.4.22.‐), aspartate endopeptidases (EC 3.4.23.‐), metalloendopeptidases (EC 3.4.24.‐) and threonine endopeptidases (EC 3.4.25.‐).

It is beyond the scope of the Guide to list all peptidase and proteinase activities; this summary focuses on selected enzymes of significant pharmacological interest.

## A1: Pepsin

**Table:** 

Nomenclature	renin
HGNC, UniProt	*REN*, P00797
EC number	3.4.23.15
Inhibitors	aliskiren (pIC_50_ 9.2) [532]

## A22: Presenilin

### Overview

Presenilin (PS)‐1 or ‐2 act as the catalytic component/essential co‐factor of the *γ*‐secretase complex responsible for the final carboxy‐terminal cleavage of amyloid precursor protein (APP) [249] in the generation of amyloid beta (A*β*) [6, 471]. Given that the accumulation and aggregation of A*β* in the brain is pivotal in the development of Alzheimer's disease (AD), inhibition of PS activity is one mechanism being investigated as a therapeutic option for AD [177]. Several small molecule inhibitors of PS‐1 have been investigated, with some reaching early clinical trials, but none have been formally approved. Dewji *et al* (2015) have reported that small peptide fragments of human PS‐1 can significantly inhibit A*β* production (total A*β*, A*β*40 and A*β*42) both *in vitro* and when infused in to the brains of APP transgenic mice [111]. The most active small peptides in this report were P4 [PMID: 25923432] and P8 [PMID: 25923432], from the amino‐terminal domain of PS‐1.

## C14: Caspase

### Overview

Caspases, (E.C. 3.4.22.‐) which derive their name from Cysteine ASPartate‐specific proteASES, include at least two families; initiator caspases (caspases 2, 8, 9 and 10), which are able to hydrolyse and activate a second family of effector caspases (caspases 3, 6 and 7), which themselves are able to hydrolyse further cellular proteins to bring about programmed cell death. Caspases are heterotetrameric, being made up of two pairs of subunits, generated by a single gene product, which is proteolysed to form the mature protein. Members of the mammalian inhibitors of apoptosis proteins (IAP) are able to bind the procaspases, thereby preventing maturation to active proteinases.

### Comments

CARD16 (Caspase recruitment domain‐containing protein 16, caspase‐1 inhibitor COP, CARD only domain‐containing protein 1, pseudo interleukin‐1*β* converting enzyme, pseudo‐ICE, ENSG00000204397) shares sequence similarity with some of the caspases.

## M1: Aminopeptidase N

### Overview

Aminopeptidases catalyze the cleavage of amino acids from the amino (N) terminus of protein or peptide substrates, and are involved in many essential cellular functions. Members of this enzyme family may be monomeric or multi‐subunit complexes, and many are zinc metalloenzymes [480].

## M2: Angiotensin‐converting (ACE and ACE2)

**Table:** 

Nomenclature	Angiotensin‐converting enzyme
Common abreviation	ACE
HGNC, UniProt	*ACE*, P12821
EC number	3.4.15.1
Endogenous substrates	angiotensin I (*AGT*, P01019) >angiotensin II (*AGT*, P01019)
Inhibitors	zofenoprilat (p*K*_i_ 9.4) [270] – Rabbit, captopril (p*K*_i_ 8.4) [331], zofenopril
Selective inhibitors	perindoprilat (pIC_50_ 9) [67], cilazaprilat (pIC_50_ 8.7) [514] – Rabbit, imidaprilat (pIC_50_ 8.7) [409], lisinopril‐tryptophan (C‐domain assay) (pIC_50_ 8.2) [515], RXP‐407 (N‐domain selective inhibition) (pIC_50_ 8.1) [434], fosinoprilat (pIC_50_ 8) [106] – Rabbit, enalaprilat (pIC_50_ 7.5) [79], benazeprilat (pIC_50_ 6.6) [282]
Comments	Reports of ACE GPI hydrolase activity [265] have been refuted [283]

## M10: Matrix metallopeptidase

### Overview

Matrix metalloproteinases (MMP) are calcium‐ and zinc‐dependent proteinases regulating the extracellular matrix and are often divided (*e.g*. [501]) on functional and structural bases into gelatinases, collagenases, stromyelinases and matrilysins, as well as membrane type‐MMP (MT‐MMP).

**Table:** 

Nomenclature	MMP2	MMP8
HGNC, UniProt	*MMP2*, P08253	*MMP8*, P22894
EC number	3.4.24.24	3.4.24.34
Selective inhibitors	ARP100 [493]	–

### Comments

A number of small molecule ‘broad spectrum’ inhibitors of MMP have been described, including marimastat and batimastat.

**Tissue inhibitors of metalloproteinase (TIMP)** proteins are endogenous inhibitors acting to chelate MMP proteins: TIMP1(*TIMP1*, P01033), TIMP2(*TIMP2*, P16035), TIMP3(*TIMP3*, P35625), TIMP4(*TIMP4*, Q99727)

## M12: Astacin/Adamalysin

### Overview

ADAM (A Disintegrin And Metalloproteinase domain containing proteins) metalloproteinases cleave cell‐surface or transmembrane proteins to generate soluble and membrane‐limited products.

ADAMTS (with thrombospondin motifs) metalloproteinases cleave cell‐surface or transmembrane proteins to generate soluble and membrane‐limited products.

### Comments

Additional ADAM family members include AC123767.2 (cDNA FLJ58962, moderately similar to mouse ADAM3, ENSG00000231168), AL160191.3 (ADAM21‐like protein, ENSG00000235812), AC136428.3‐2 (ENSG00000185520) and ADAMDEC1 (decysin 1, ENSG00000134028).

Other ADAMTS family members include AC104758.12‐5 (FLJ00317 protein Fragment ENSG00000231463), AC139425.3‐1 (ENSG00000225577), and AC126339.6‐1 (ENSG00000225734).

## M28: Aminopeptidase Y

**Table:** 

Nomenclature	Folate hydrolase (prostate‐specific membrane antigen) 1
HGNC, UniProt	*FOLH1*, Q04609
EC number	3.4.17.21
Antibodies	capromab (Binding)

### Comment

folate hydrolase is also known as NAALADase as it is responsible for the hydrolysis of N‐acetaspartylglutamate to form N‐acetylaspartate and L‐glutamate. In the gut, the enzyme assists in the assimilation of folate by hydrolysing dietary poly‐gamma‐glutamylfolate. The enzyme is highly expressed in the prostate, and its expression is up‐regulated in cancerous tissue. A tagged version of the antibody capromab has been used for imaging purposes.

## M19: Membrane dipeptidase

**Table:** 

Nomenclature	Dipeptidase 1
HGNC, UniProt	*DPEP1*, P16444
EC number	3.4.13.19: LTD_4_ + H_2_O = LTE_4_ + glycine
Inhibitors	cilastatin (p*K*_i_ 6) [179] – Unknown

## S1: Chymotrypsin

**Table:** 

Nomenclature	complement component 1, r subcomponent	coagulation factor II (thrombin)	coagulation factor X	elastase, neutrophil expressed
HGNC, UniProt	*C1R*, P00736	*F2*, P00734	*F10*, P00742	*ELANE*, P08246
EC number	3.4.21.41	3.4.21.5	3.4.21.6	3.4.21.37
Inhibitors	nafamostat (pIC_50_ 4.9) [203]	lepirudin (p*K*_i_ 13) [511], desirudin (p*K*_i_ 12.7) [242], AZ12971554 (p*K*_i_ 9.5) [16], melagatran (p*K*_i_ 8.7) [186], bivalirudin (p*K*_i_ 8.6) [527], dabigatran (p*K*_i_ 8.3) [198], argatroban (p*K*_i_ 7.7) [225]	rivaroxaban (p*K*_i_ 9.4) [380], edoxaban (p*K*_i_ 9.2) [385], apixaban (p*K*_i_ 9.1) [528]	alvelestat (p*K*_i_ 8) [463], sivelestat (pIC_50_ 7.4) [96]
Selective inhibitors	–	AR‐H067637 (pIC_50_ 8.4) [108]	–	–

**Table:** 

Nomenclature	plasminogen	plasminogen activator, tissue	protease, serine, 1 (trypsin 1)	tryptase alpha/beta 1
HGNC, UniProt	*PLG*, P00747	*PLAT*, P00750	*PRSS1*, P07477	*TPSAB1*, Q15661
EC number	3.4.21.7	3.4.21.68	3.4.21.4	3.4.21.59
Inhibitors	aprotinin {Bovine} (Binding) (pIC_50_ 6.8) [454], tranexamic acid (Binding) (pIC_50_ 3.6) [454], 6‐aminocaproic acid (Binding)	6‐aminocaproic acid	nafamostat (pIC_50_ 7.8) [203]	nafamostat (pIC_50_ 10) [342]
Selective inhibitors	–	–	–	gabexate (pIC_50_ 8.5) [127]

## T1: Proteasome

### Overview

The T1 macropain beta subunits form the catalytic proteinase core of the 20S proteasome complex [86]. This catalytic core enables the degradation of peptides with Arg, Phe, Tyr, Leu, and Glu adjacent to the cleavage site. The *β*5 subunit is the principal target of the approved drug proteasome inhibitor bortezomib.

**Table:** 

Nomenclature	proteasome (prosome, macropain) subunit, beta type, 5
HGNC, UniProt	*PSMB5*, P28074
EC number	3.4.25.1
Inhibitors	bortezomib (pIC_50_ 7.7) [347]
Selective inhibitors	ixazomib (p*K*_i_ 9) [273]

## S8: Subtilisin

### Overview

One member of this family has garnered intense interest as a clinical drug target. As liver PCSK9 acts to maintain cholesterol homeostasis, it has become a target of intense interest for clinical drug development. Inhibition of PCSK9 can lower low‐density cholesterol (LDL‐C) by clearing LDLR‐bound LDL particles, thereby lowering circulating cholesterol levels. It is hypothesised that this action may improve outcomes in patients with atherosclerotic cardiovascular disease [297, 416, 462]. Therapeutics which inhibit PCSK9 are viewed as potentially lucrative replacements for statins, upon statin patent expiry. Several monoclonal antibodies including alirocumab, evolocumab, bococizumab, RG‐7652 and LY3015014 are under development. One RNAi therapeutic, code named ALN‐PCS02, is also in development [99, 139, 146].

## S9: Prolyl oligopeptidase

**Table:** 

Nomenclature	dipeptidyl‐peptidase 4
HGNC, UniProt	*DPP4*, P27487
EC number	3.4.14.5
Endogenous substrates	glucagon‐like peptide 1 (*GCG*, P01275)
Inhibitors	saxagliptin (p*K*_i_ 9.2) [184], linagliptin (p*K*_i_ 9) [122], sitagliptin (pIC_50_ 8.1) [104], vildagliptin (p*K*_i_ 7.8) [184]

## Acetylcholine turnover

### Overview

Acetylcholine is familiar as a neurotransmitter in the central nervous system and in the periphery. In the somatic nervous system, it activates nicotinic acetylcholine receptors at the skeletal neuromuscular junction. It is also employed in the autonomic nervous system, in both parasympathetic and sympathetic branches; in the former, at the smooth muscle neuromuscular junction, activating muscarinic acetylcholine receptors. In the latter, acetylcholine is involved as a neurotransmitter at the ganglion, activating nicotinic acetylcholine receptors. Acetylcholine is synthesised in neurones through the action of choline O‐acetyltransferase and metabolised after release through the extracellular action of acetylcholinesterase and cholinesterase. Choline is accumulated from the extracellular medium by selective transporters (see SLC5A7 and the SLC44 family). Acetylcholine is accumulated in synaptic vesicles through the action of the vesicular acetylcholine transporter SLC18A3.

**Table:** 

Nomenclature	choline O‐acetyltransferase	acetylcholinesterase (Yt blood group)	butyrylcholinesterase
Common abreviation	ChAT	AChE	BChE
HGNC, UniProt	*CHAT*, P28329	*ACHE*, P22303	*BCHE*, P06276
EC number	2.3.1.6: acetyl CoA + choline = acetylcholine + coenzyme A	3.1.1.7: acetylcholine + H_2_O = acetic acid + choline + H^+^	3.1.1.7: acetylcholine + H_2_O = acetic acid + choline + H^+^
Inhibitors	–	tacrine (p*K*_i_ 7.5) [52], galantamine (pIC_50_ 6.3) [87], rivastigmine (pIC_50_ 5.4) [305]	rivastigmine (pIC_50_ 7.4) [305], tacrine (p*K*_i_ 7.2) [52]
(Sub)family‐selective inhibitors	–	physostigmine (pIC_50_ 7.6–7.8) [305]	physostigmine (pIC_50_ 7.6–7.8) [305]
Selective inhibitors	–	donepezil (pIC_50_ 7.7–8.3) [62, 160, 305], BW284C51 (pIC_50_ 7.7) [172]	bambuterol (pIC_50_ 8.5) [172]
Comments	Splice variants of choline O‐acetyltransferase are suggested to be differentially distributed in the periphery and CNS (see [28]).	–	–

### Comments

A number of organophosphorus compounds inhibit acetylcholinesterase and cholinesterase irreversibly, including pesticides such as chlorpyrifos‐oxon, and nerve agents such as tabun, soman and sarin. AChE is unusual in its exceptionally high turnover rate which has been calculated at 740 000/min/molecule [505].

### Further Reading

Abreu‐Villaça Y *et al*. (2011) Developmental aspects of the cholinergic system. *Behav. Brain Res.* **221**: 367‐78 [PMID:20060019]

Bellier JP *et al*. (2011) Peripheral type of choline acetyltransferase: biological and evolutionary implications for novel mechanisms in cholinergic system. *J. Chem. Neuroanat.* **42**: 225‐35 [PMID:21382474]

Engel AG *et al*. (2015) Congenital myasthenic syndromes: pathogenesis, diagnosis, and treatment. *Lancet Neurol* **14**: 420‐34 [PMID:25792100]

Rosini M *et al*. (2014) Multi‐target design strategies in the context of Alzheimer's disease: acetylcholinesterase inhibition and NMDA receptor antagonism as the driving forces. *Neurochem. Res.* **39**: 1914‐23 [PMID:24493627]

Zimmermann M. (2013) Neuronal AChE splice variants and their non‐hydrolytic functions: redefining a target of AChE inhibitors? *Br. J. Pharmacol.* **170**: 953‐67 [PMID:23991627]

## Adenosine turnover

### Overview

A multifunctional, ubiquitous molecule, adenosine acts at cell‐surface G protein‐coupled receptors, as well as numerous enzymes, including protein kinases and adenylyl cyclase. Extracellular adenosine is thought to be produced either by export or by metabolism, predominantly through ecto‐5'‐nucleotidase activity (also producing inorganic phosphate). It is inactivated either by extracellular metabolism *via*adenosine deaminase (also producing ammonia) or, following uptake by nucleoside transporters, *via*adenosine deaminase or adenosine kinase (requiring ATP as co‐substrate). Intracellular adenosine may be produced by cytosolic 5'‐nucleotidases or through S‐adenosylhomocysteine hydrolase (also producing L‐homocysteine).

**Table:** 

Nomenclature	Adenosine deaminase	Adenosine kinase	Ecto‐5'‐Nucleotidase	S‐Adenosylhomocysteine hydrolase
Common abreviation	ADA	ADK	NT5E	SAHH
HGNC, UniProt	*ADA*, P00813	*ADK*, P55263	*NT5E*, P21589	*AHCY*, P23526
EC number	3.5.4.4: adenosine + H_2_O = inosine + NH_3_	2.7.1.20	3.1.3.5	3.3.1.1
Endogenous substrates	–	–	–	S‐adenosylhomocysteine
Rank order of affinity	2'‐deoxyadenosine>adenosine	adenosine	adenosine 5'‐monophosphate, 5'‐GMP, 5'‐inosine monophosphate, 5'‐UMP>5'‐dAMP, 5'‐dGMP	–
Products	2'‐deoxyinosine, inosine	adenosine 5'‐monophosphate	uridine, inosine, guanine, adenosine	adenosine
Inhibitors	–	–	–	DZNep (p*K*_i_ 12.3) [174] – Hamster
Selective inhibitors	pentostatin (pIC_50_ 10.8) [3], EHNA (p*K*_i_ 8.8) [3]	A134974 (pIC_50_ 10.2) [325], ABT702 (pIC_50_ 8.8) [236]	*αβ*‐methyleneADP (pIC_50_ 8.7) [50]	3‐deazaadenosine (pIC_50_ 8.5) [185]

### Comments

An extracellular adenosine deaminase activity, termed ADA2 or adenosine deaminase growth factor (ADGF, *CECR1*, Q9NZK5) has been identified [94, 309], which is insensitive to EHNA[546]. Other forms of adenosine deaminase act on ribonucleic acids and may be divided into two families: *ADAT1*(Q9BUB4) deaminates transfer RNA; *ADAR*(EC 3.5.4.37, also known as 136 kDa double‐stranded RNA‐binding protein, P136, K88DSRBP, Interferon‐inducible protein 4); *ADARB1* (EC 3.5.‐.‐, , also known as dsRNA adenosine deaminase) and *ADARB2* (EC 3.5.‐.‐, also known as dsRNA adenosine deaminase B2, RNA‐dependent adenosine deaminase 3) act on double‐stranded RNA. Particular polymorphisms of the ADA gene result in loss‐of‐function and severe combined immunodeficiency syndrome. Adenosine deaminase is able to complex with dipeptidyl peptidase IV (EC 3.4.14.5, *DPP4*, also known as T‐cell activation antigen CD26, TP103, adenosine deaminase complexing protein 2) to form a cell‐surface activity [248].

### Further Reading

Antonioli L *et al*. (2013) CD39 and CD73 in immunity and inflammation. *Trends Mol Med* **19**: 355‐67 [PMID:23601906]

Boison D. (2013) Adenosine kinase: exploitation for therapeutic gain. *Pharmacol. Rev.* **65**: 906‐43 [PMID:23592612]

Cortés A *et al*. (2015) Moonlighting adenosine deaminase: a target protein for drug development. *Med Res Rev* **35**: 85‐125 [PMID:24933472]

Tomaselli S *et al*. (2014) The RNA editing enzymes ADARs: mechanism of action and human disease. *Cell Tissue Res.* **356**: 527‐32 [PMID:24770896]

Zimmermann H *et al*. (2012) Cellular function and molecular structure of ecto‐nucleotidases. *Purinergic Signal.* **8**: 437‐502 [PMID:22555564]

## Amino acid hydroxylases

### Overview

The amino acid hydroxylases (monooxygenases), EC.1.14.16.‐, are iron‐containing enzymes which utilise molecular oxygen and sapropterin as co‐substrate and co‐factor, respectively. In humans, as well as in other mammals, there are two distinct L‐Tryptophan hydroxylase 2 genes. In humans, these genes are located on chromosomes 11 and 12 and encode two different homologous enzymes, TPH1 and TPH2.

**Table:** 

Nomenclature	L‐Phenylalanine hydroxylase	L‐Tyrosine hydroxylase	L‐Tryptophan hydroxylase 1	L‐Tryptophan hydroxylase 2
HGNC, UniProt	*PAH*, P00439	*TH*, P07101	*TPH1*, P17752	*TPH2*, Q8IWU9
EC number	1.14.16.1: L‐phenylalanine + O_2_ ‐>L‐tyrosine	1.14.16.2: L‐tyrosine + O_2_ ‐>levodopa	1.14.16.4	1.14.16.4
Endogenous activators	Protein kinase A‐mediated phosphorylation (Rat) [1]	Protein kinase A‐mediated phosphorylation [239]	Protein kinase A‐mediated phosphorylation [240]	Protein kinase A‐mediated phosphorylation [240]
Endogenous substrates	L‐phenylalanine	L‐tyrosine	L‐tryptophan	L‐tryptophan
Products	L‐tyrosine	levodopa	5‐hydroxy‐L‐tryptophan	5‐hydroxy‐L‐tryptophan
Cofactors	sapropterin	sapropterin, Fe^2+^	–	–
Selective activators	sapropterin (p*K*_i_ 5.4) [481]	–	–	–
Inhibitors	–	methyltyrosine	–	–
Selective inhibitors	*α*‐methylphenylalanine [180] – Rat, fenclonine	*α*‐propyldopacetamide, 3‐chlorotyrosine, 3‐iodotyrosine, alpha‐methyltyrosine	*α*‐propyldopacetamide, 6‐fluorotryptophan [352], fenclonine, fenfluramine	*α*‐propyldopacetamide, 6‐fluorotryptophan [352], fenclonine, fenfluramine
Comments	PAH is an iron bound homodimer or ‐tetramer from the same structural family as tyrosine 3‐monooxygenase and the tryptophan hydroxylases. Deficiency or loss‐of‐function of PAH is associated with phenylketonuria	TH is a homotetramer, which is inhibited by dopamine and other catecholamines in a physiological negative feedback pathway [102].	–	–

### Further Reading

Amireault P *et al*. (2013) Life without peripheral serotonin: insights from tryptophan hydroxylase 1 knockout mice reveal the existence of paracrine/autocrine serotonergic networks. *ACS Chem Neurosci* **4**: 64‐71 [PMID:23336045]

Daubner SC *et al*. (2011) Tyrosine hydroxylase and regulation of dopamine synthesis. *Arch. Biochem. Biophys.* **508**: 1‐12 [PMID:21176768]

Flydal MI *et al*. (2013) Phenylalanine hydroxylase: function, structure, and regulation. *IUBMB Life* **65**: 341‐9 [PMID:23457044]

Roberts KM *et al*. (2013) Mechanisms of tryptophan and tyrosine hydroxylase. *IUBMB Life* **65**: 350‐7 [PMID:23441081]

Tekin I *et al*. (2014) Complex molecular regulation of tyrosine hydroxylase. *J Neural Transm* **121**: 1451‐81 [PMID:24866693]

## L‐Arginine turnover

### Overview

L‐arginine is a basic amino acid with a guanidino sidechain. As an amino acid, metabolism of L‐arginine to form L‐ornithine, catalysed by arginase, forms the last step of the urea production cycle. L‐Ornithine may be utilised as a precursor of polyamines (see Carboxylases and Decarboxylases) or recycled via L‐argininosuccinic acid to L‐arginine. L‐Arginine may itself be decarboxylated to form agmatine, although the prominence of this pathway in human tissues is uncertain. L‐Arginine may be used as a precursor for guanidoacetic acid formation in the creatine synthesis pathway under the influence of arginine:glycine amidinotransferase with L‐ornithine as a byproduct. Nitric oxide synthase uses L‐arginine to generate nitric oxide, with L‐citrulline also as a byproduct.

L‐Arginine in proteins may be subject to post‐translational modification through methylation, catalysed by protein arginine methyltransferases. Subsequent proteolysis can liberate asymmetric N^G^,N^G^‐dimethyl‐L‐arginine(ADMA), which is an endogenous inhibitor of nitric oxide synthase activities. ADMA is hydrolysed by dimethylarginine dimethylhydrolase activities to generate L‐citrulline and dimethylamine.

### Further Reading

Moncada S *et al*. (1997) International Union of Pharmacology Nomenclature in Nitric Oxide Research. *Pharmacol. Rev.* **49**: 137‐42 [PMID:9228663]

Tang L *et al*. (2014) Targeting NOS as a therapeutic approach for heart failure. *Pharmacol. Ther.* **142**: 306‐15 [PMID:24380841]

Tratsiakovich Y *et al*. (2013) Arginase as a target for treatment of myocardial ischemia‐reperfusion injury. *Eur. J. Pharmacol.* **720**: 121‐3 [PMID:24183975]

Whiteley CG. (2014) Arginine metabolising enzymes as targets against Alzheimers' disease. *Neurochem. Int.* **67**: 23‐31 [PMID:24508404]

Yang Y *et al*. (2013) Protein arginine methyltransferases and cancer. *Nat. Rev. Cancer* **13**: 37‐50 [PMID:23235912]

## Arginase

### Overview

Arginase (EC 3.5.3.1) are manganese‐containing isoforms, which appear to show differential distribution, where the ARG1 isoform predominates in the liver and erythrocytes, while ARG2 is associated more with the kidney.

### Comments

N^*ω*^‐hydroxyarginine, an intermediate in NOS metabolism of L‐arginine acts as a weak inhibitor and may function as a physiological regulator of arginase activity. Although isoform‐selective inhibitors of arginase are not available, examples of inhibitors selective for arginase compared to NOS are N^*ω*^‐hydroxy‐nor‐L‐arginine[483], S‐(2‐boronoethyl)‐L‐cysteine[90, 256] and 2(S)‐amino‐6‐boronohexanoic acid [22, 90].

## Arginine:glycine amidinotransferase

**Table:** 

Nomenclature	Arginine:glycine amidinotransferase
Common abreviation	AGAT
HGNC, UniProt	*GATM*, P50440
EC number	2.1.4.1

## Dimethylarginine dimethylaminohydrolases

### Overview

Dimethylarginine dimethylaminohydrolases (DDAH, EC 3.5.3.18) are cytoplasmic enzymes which hydrolyse N^G^,N^G^‐dimethyl‐L‐arginine to form dimethylamine and L‐citrulline.

**Table:** 

Nomenclature	*N*^G^,*N*^G^‐Dimethylarginine dimethylaminohydrolase 1	*N*^G^,*N*^G^‐Dimethylarginine dimethylaminohydrolase 2
Common abreviation	DDAH1	DDAH2
HGNC, UniProt	*DDAH1*, O94760	*DDAH2*, O95865
EC number	3.5.3.18	3.5.3.18
Cofactors	Zn^2+^	–

## Nitric oxide synthases

### Overview

Nitric oxide synthases (NOS, E.C. 1.14.13.39) utilise L‐arginine(not D‐arginine) and molecular oxygen to generate nitric oxide and L‐citrulline. The nomenclature suggested by **NC‐IUPHAR**of NOS I, II and III [340] has not gained wide acceptance. eNOS and nNOS isoforms are activated at concentrations of calcium greater than 100 nM, while iNOS shows higher affinity for Ca^2+^/calmodulin(*CALM1* *CALM2* *CALM3*, P62158) and thus appears to be constitutively active. All the three isoforms are homodimers and require sapropterin, flavin adenine dinucleotide, flavin mononucleotide and NADPH for catalytic activity. L‐NAME is an inhibitor of all three isoforms, with an IC_50_ value in the micromolar range.

**Table:** 

Nomenclature	Endothelial NOS	Inducible NOS	Neuronal NOS
Common abreviation	eNOS	iNOS	nNOS
HGNC, UniProt	*NOS3*, P29474	*NOS2*, P35228	*NOS1*, P29475
EC number	1.14.13.39	1.14.13.39	1.14.13.39
Inhibitors	–	–	N^*ω*^propyl‐L‐arginine (p*K*_i_ 7.2) [548] – Rat
Selective inhibitors	–	1400W (pIC_50_ 8.2) [168], 2‐amino‐4‐methylpyridine (pIC_50_ 7.4) [131], PIBTU (pIC_50_ 7.3) [169], NIL (pIC_50_ 5.5) [341], aminoguanidine [92]	3‐bromo‐7NI (pIC_50_ 6.1–6.5) [40], 7NI (pIC_50_ 5.3) [19]

### Comments

The reductase domain of NOS catalyses the reduction of cytochrome c and other redox‐active dyes [322]. NADPH:O_2_ oxidoreductase catalyses the formation of superoxide anion/H_2_O_2_ in the absence of L‐arginine and sapropterin.

## Carboxylases and decarboxylases

## Carboxylases

### Overview

The carboxylases allow the production of new carbon‐carbon bonds by introducing HCO_3_^−^ or CO_2_ into target molecules. Two groups of carboxylase activities, some of which are bidirectional, can be defined on the basis of the cofactor requirement, making use of biotin (EC 6.4.1.‐) or vitamin K hydroquinone (EC 4.1.1.‐).

**Table:** 

Nomenclature	Pyruvate carboxylase	Acetyl‐CoA carboxylase 1	Acetyl‐CoA carboxylase 2
Common abreviation	PC	ACC1	ACC2
HGNC, UniProt	*PC*, P11498	*ACACA*, Q13085	*ACACB*, O00763
EC number	6.4.1.1	6.4.1.2	6.4.1.2
Endogenous substrates	ATP, pyruvic acid	ATP, acetyl CoA	acetyl CoA, ATP
Products	P_i_, adenosine diphosphate, oxalacetic acid	P_i_, adenosine diphosphate, malonyl‐CoA	P_i_, adenosine diphosphate, malonyl‐CoA
Cofactors	biotin	biotin	biotin
Selective inhibitors	–	TOFA [294]	TOFA [294]
Comments	–	Citrate and other dicarboxylic acids are allosteric activators of acetyl‐CoA carboxylase.	Citrate and other dicarboxylic acids are allosteric activators of acetyl‐CoA carboxylase.

**Table:** 

Nomenclature	Propionyl‐CoA carboxylase	*γ*‐Glutamyl carboxylase
Common abreviation	PCCA, PCCB	GGCX
HGNC, UniProt	*PCCA*, P05165	*GGCX*, P38435
	*PCCB*, P05166	
Subunits	Propionyl‐CoA carboxylase *α* subunit	
	Propionyl‐CoA carboxylase *β* subunit	
		–
EC number	6.4.1.3	4.1.1.90
Endogenous substrates	propionyl‐CoA, ATP	glutamyl peptides
Products	adenosine diphosphate, methylmalonyl‐CoA, P_i_	carboxyglutamyl peptides
Cofactors	biotin	vitamin K hydroquinone, NADPH
Inhibitors	–	anisindione
Comments	Propionyl‐CoA carboxylase is able to function in both forward and reverse activity modes, as a ligase (carboxylase) or lyase (decarboxylase), respectively.	Loss‐of‐function mutations in *γ*‐glutamyl carboxylase are associated with clotting disorders.

### Comments

Dicarboxylic acids including citric acid are able to activate ACC1/ACC2 activity allosterically. PCC is able to function in forward and reverse modes as a ligase (carboxylase) or lyase (decarboxylase) activity, respectively. Loss‐of‐function mutations in GGCX are associated with clotting disorders.

## Decarboxylases

### Overview

The decarboxylases generate CO_2_ and the indicated products from acidic substrates, requiring pyridoxal phosphate or pyruvic acid as a co‐factor.

**Table:** 

Nomenclature	S‐Adenosylmethionine	L‐Arginine	L‐Aromatic amino‐acid	Glutamic acid	Glutamic acid
	decarboxylase	decarboxylase	decarboxylase	decarboxylase 1	decarboxylase 2
Common abreviation	SAMDC	ADC	AADC	GAD1	GAD2
HGNC, UniProt	*AMD1*, P17707	*AZIN2*, Q96A70	*DDC*, P20711	*GAD1*, Q99259	*GAD2*, Q05329
EC number	4.1.1.50	4.1.1.19	4.1.1.28: levodopa ‐>dopamine + CO_2_ 5‐hydroxy‐L‐tryptophan ‐>5‐hydroxytryptamine + CO_2_ This enzyme also catalyses the following reaction:: L‐tryptophan ‐>tryptamine + CO_2_	4.1.1.15: L‐glutamic acid + H^+^ ‐>GABA + CO_2_	4.1.1.15: L‐glutamic acid + H^+^ ‐>GABA + CO_2_
Substrates	–	–	Deuterium‐substituted	–	–
			L‐DOPA [312]		
Endogenous substrates	S‐adenosyl methionine	L‐arginine	levodopa, 5‐hydroxy‐L‐tryptophan, L‐tryptophan	L‐glutamic acid, L‐aspartic acid	L‐glutamic acid, L‐aspartic acid
Products	S‐adenosyl‐L‐	agmatine [552]	5‐hydroxytryptamine,	GABA	GABA
	methioninamine		dopamine		
Cofactors	pyruvic acid	pyridoxal phosphate	pyridoxal phosphate	pyridoxal phosphate	pyridoxal phosphate
Selective inhibitors	sardomozide (pIC_50_ 8) [455]	–	3‐hydroxybenzylhydrazine, L‐*α*‐methyldopa, benserazide [101], carbidopa	s‐allylglycine	s‐allylglycine
Comments	s‐allylglycine is also an inhibitor of SAMDC [368].	The presence of a functional ADC activity in human tissues has been questioned [89].	AADC is a homodimer.	L‐aspartic acid is a less rapidly metabolised substrate of mouse brain glutamic acid decarboxylase generating *β*‐alanine [530]. Autoantibodies against GAD1 and GAD2 are elevated in type 1 diabetes mellitus and neurological disorders (see Further reading).	L‐aspartic acid is a less rapidly metabolised substrate of mouse brain glutamic acid decarboxylase generating *β*‐alanine [530]. Autoantibodies against GAD1 and GAD2 are elevated in type 1 diabetes mellitus and neurological disorders (see Further reading).

**Table:** 

Nomenclature	Histidine decarboxylase	Malonyl‐CoA decarboxylase	Ornithine decarboxylase	Phosphatidylserine decarboxylase
Common abreviation	HDC	MLYCD	ODC	PSDC
HGNC, UniProt	*HDC*, P19113	*MLYCD*, O95822	*ODC1*, P11926	*PISD*, Q9UG56
EC number	4.1.1.22	4.1.1.9	4.1.1.17	4.1.1.65
Endogenous substrates	L‐histidine	malonyl‐CoA	L‐ornithine	phosphatidylserine
Products	histamine	acetyl CoA	putrescine	phosphatidylethanolamine
Cofactors	pyridoxal phosphate	pyridoxal phosphate	pyridoxal phosphate	pyruvic acid
Selective inhibitors	AMA, FMH [164]	–	APA (pIC_50_ 7.5) [456], eflornithine (p*K*_d_ 4.9) [394]	–
Comments	–	Inhibited by AMP‐activated protein kinase‐evoked phosphorylation [415]	The activity of ODC is regulated by the presence of an antizyme (ENSG00000104904) and an ODC antizyme inhibitor (ENSG00000155096).	S‐allylglycine is also an inhibitor of SAMDC [368].

### Further Reading

Bale S *et al*. (2010) Structural biology of S‐adenosylmethionine decarboxylase. *Amino Acids* **38**: 451‐60 [PMID:19997761]

Jitrapakdee S *et al*. (2008) Structure, mechanism and regulation of pyruvate carboxylase. *Biochem. J.* **413**: 369‐87 [PMID:18613815]

Lietzan AD *et al*. (2014) Functionally diverse biotin‐dependent enzymes with oxaloacetate decarboxylase activity. *Arch. Biochem. Biophys.* **544**: 75‐86 [PMID:24184447]

Moya‐García AA *et al*. (2009) Structural features of mammalian histidine decarboxylase reveal the basis for specific inhibition. *Br. J. Pharmacol.* **157**: 4‐13 [PMID:19413567]

Tong L. (2013) Structure and function of biotin‐dependent carboxylases. *Cell. Mol. Life Sci.* **70**: 863‐91 [PMID:22869039]

Vance JE *et al*. (2013) Formation and function of phosphatidylserine and phosphatidylethanolamine in mammalian cells. *Biochim. Biophys. Acta* **1831**: 543‐54 [PMID:22960354]

## Catecholamine turnover

### Overview

Catecholamines are defined by the presence of two adjacent hydroxyls on a benzene ring with a sidechain containing an amine. The predominant catacholamines in mammalian biology are the neurotransmitter/hormones dopamine, (‐)‐noradrenaline (norepinephrine) and (‐)‐adrenaline(epinephrine). These hormone/transmitters are synthesized by sequential metabolism from L‐phenylalanine via L‐tyrosine. Hydroxylation of L‐tyrosine generates levodopa, which is decarboxylated to form dopamine. Hydroxylation of the ethylamine sidechain generates (‐)‐noradrenaline (norepinephrine), which can be methylated to form (‐)‐adrenaline(epinephrine). In particular neuronal and adrenal chromaffin cells, the catecholamines dopamine, (‐)‐noradrenaline and (‐)‐adrenaline are accumulated into vesicles under the influence of the vesicular monoamine transporters (VMAT1/SLC18A1 and VMAT2/SLC18A2). After release into the synapse or the bloodstream, catecholamines are accumulated through the action cell‐surface transporters, primarily the dopamine (DAT/SLC6A3) and norepinephrine transporter (NET/SLC6A2). The primary routes of metabolism of these catecholamines are oxidation via monoamine oxidase activities of methylation via catechol O‐methyltransferase.

**Table:** 

Nomenclature	L‐Phenylalanine hydroxylase	Tyrosine aminotransferase	L‐Aromatic amino‐acid decarboxylase
Common abreviation	–	TAT	AADC
HGNC, UniProt	*PAH*, P00439	*TAT*, P17735	*DDC*, P20711
EC number	1.14.16.1: L‐phenylalanine + O_2_ ‐>L‐tyrosine	2.6.1.5: L‐tyrosine + *α*‐ketoglutaric acid ‐>4‐hydroxyphenylpyruvic acid + L‐glutamic acid	4.1.1.28: levodopa ‐>dopamine + CO_2_ 5‐hydroxy‐L‐tryptophan ‐>5‐hydroxytryptamine + CO_2_ This enzyme also catalyses the following reaction:: L‐tryptophan ‐>tryptamine + CO_2_
Endogenous activators	Protein kinase A‐mediated phosphorylation (Rat) [1]	–	–
Substrates	–	–	Deuterium‐substituted L‐DOPA [312]
Endogenous substrates	L‐phenylalanine	–	levodopa, 5‐hydroxy‐L‐tryptophan, L‐tryptophan
Products	L‐tyrosine	–	5‐hydroxytryptamine, dopamine
Cofactors	sapropterin	pyridoxal phosphate	pyridoxal phosphate
Selective activators	sapropterin (p*K*_i_ 5.4) [481]	–	–
Selective inhibitors	*α*‐methylphenylalanine [180] – Rat, fenclonine	–	3‐hydroxybenzylhydrazine, L‐*α*‐methyldopa, benserazide [101], carbidopa
Comments	PAH is an iron bound homodimer or ‐tetramer from the same structural family as tyrosine 3‐monooxygenase and the tryptophan hydroxylases. Deficiency or loss‐of‐function of PAH is associated with phenylketonuria	Tyrosine may also be metabolized in the liver by tyrosine transaminase to generate 4‐hydroxyphenylpyruvic acid, which can be further metabolized to homogentisic acid. TAT is a homodimer, where loss‐of‐function mutations are associated with type II tyrosinemia.	AADC is a homodimer.

**Table:** 

Nomenclature	L‐Tyrosine hydroxylase	Dopamine beta‐hydroxylase	Phenylethanolamine N‐methyltransferase
		(dopamine beta‐monooxygenase)	
Common abreviation	–	DBH	PNMT
HGNC, UniProt	*TH*, P07101	*DBH*, P09172	*PNMT*, P11086
EC number	1.14.16.2: L‐tyrosine + O_2_ ‐>levodopa	1.14.17.1: dopamine + O_2_ = (‐)‐noradrenaline + H_2_O	2.1.1.28: (‐)‐noradrenaline ‐>(‐)‐adrenaline
Endogenous activators	Protein kinase A‐mediated phosphorylation [239]	–	–
Endogenous substrates	L‐tyrosine	–	–
Products	levodopa	–	–
Cofactors	sapropterin, Fe^2+^	Cu^2+^, L‐ascorbic acid	S‐adenosyl methionine
Inhibitors	methyltyrosine	nepicastat (pIC_50_ 8) [458]	LY134046 (p*K*_i_ 7.6) [154]
Selective inhibitors	*α*‐propyldopacetamide, 3‐chlorotyrosine, 3‐iodotyrosine, alpha‐methyltyrosine	–	–
Comments	TH is a homotetramer, which is inhibited by dopamine and other catecholamines in a physiological negative feedback pathway [102].	DBH is a homotetramer. A protein structurally‐related to DBH (*MOXD1*, Q6UVY6) has been described and for which a function has yet to be identified [71].	–

**Table:** 

Nomenclature	Monoamine oxidase A	Monoamine oxidase B	Catechol‐O‐methyltransferase
Common abreviation	MAO‐A	MAO‐B	COMT
HGNC, UniProt	*MAOA*, P21397	*MAOB*, P27338	*COMT*, P21964
EC number	1.4.3.4 (‐)‐adrenaline ‐>3,4‐dihydroxymandelic acid + NH_3_ (‐)‐noradrenaline ‐>3,4‐dihydroxymandelic acid + NH_3_ tyramine ‐>4‐hydroxyphenyl acetaldehyde + NH_3_ dopamine ‐>3,4‐dihydroxyphenylacetaldehyde + NH_3_ 5‐hydroxytryptamine ‐>5‐hydroxyindole acetaldehyde + NH_3_	1.4.3.4	2.1.1.6: S‐adenosyl‐L‐methionine + a catechol = S‐adenosyl‐L‐homocysteine + a guaiacol (‐)‐noradrenaline ‐>normetanephrine dopamine ‐>3‐methoxytyramine 3,4‐dihydroxymandelic acid ‐>vanillylmandelic acid (‐)‐adrenaline ‐>metanephrine
Cofactors	flavin adenine dinucleotide	flavin adenine dinucleotide	S‐adenosyl methionine
Inhibitors	moclobemide (p*K*_i_ 8.3) [234], phenelzine (Irreversible inhibition) (p*K*_i_ 7.3) [36], tranylcypromine (pIC_50_ 4.7) [538], selegiline (p*K*_i_ 4.2) [333], befloxatone [100], clorgiline, pirlindole [327]	rasagiline (pIC_50_ 7.8) [542], phenelzine (Irreversible inhibition) (p*K*_i_ 7.8) [36], lazabemide (p*K*_i_ 7.1) [188, 489], selegiline (p*K*_i_ 5.7–6) [113, 333], tranylcypromine (pIC_50_ 4.7) [538]	tolcapone (soluble enzyme) (p*K*_i_ 9.6) [299], tolcapone (membrane‐bound enzyme) (p*K*_i_ 9.5) [299], entacapone (soluble enzyme) (p*K*_i_ 9.5) [299], entacapone (membrane‐bound enzyme) (p*K*_i_ 8.7) [299]
Selective inhibitors	–	safinamide (p*K*_i_ 6.3) [35]	–
Comments	–	–	COMT appears to exist in both membrane‐bound and soluble forms. COMT has also been described to methylate steroids, particularly hydroxyestradiols

### Further Reading

Al‐Nuaimi SK *et al*. (2012) Monoamine oxidase inhibitors and neuroprotection: a review. *Am J Ther* **19**: 436‐48 [PMID:22960850]

Fitzpatrick PF. (2012) Allosteric regulation of phenylalanine hydroxylase. *Arch. Biochem. Biophys.* **519**: 194‐201 [PMID:22005392]

Flydal MI *et al*. (2013) Phenylalanine hydroxylase: function, structure, and regulation. *IUBMB Life* **65**: 341‐9 [PMID:23457044]

Ma Z *et al*. (2013) Structure‐based drug design of catechol‐O‐methyltransferase inhibitors for CNS disorders. *Br J Clin Pharmacol* [PMID:23713800]

Shih JC *et al*. (2011) Transcriptional regulation and multiple functions of MAO genes. *J Neural Transm* **118**: 979‐86 [PMID:21359973]

## Ceramide turnover

### Overview

Ceramides are a family of sphingophospholipids synthesized in the endoplasmic reticulum, which mediate cell stress responses, including apoptosis, autophagy and senescence, Serine palmitoyltransferase generates 3‐ketosphinganine, which is reduced to sphinganine(dihydrosphingosine). N‐Acylation allows the formation of dihydroceramides, which are subsequently reduced to form ceramides. Once synthesized, ceramides are trafficked from the ER to the Golgi bound to the ceramide transfer protein, CERT (*COL4A3BP*, Q9Y5P4). Ceramide can be metabolized via multiple routes, ensuring tight regulation of its cellular levels. Addition of phosphocholine generates sphingomyelin while carbohydrate is added to form glucosyl‐ or galactosylceramides. Ceramidase re‐forms sphingosine or sphinganine from ceramide or dihydroceramide. Phosphorylation of ceramide generates ceramide phosphate. The determination of accurate kinetic parameters for many of the enzymes in the sphingolipid metabolic pathway is complicated by the lipophilic nature of the substrates.

## Serine palmitoyltransferase

### Overview

The functional enzyme is a heterodimer of SPT1 (LCB1) with either SPT2 (LCB2) or SPT3 (LCB2B); the small subunits of SPT (ssSPTa or ssSPTb) bind to the heterodimer to enhance enzymatic activity. The complexes of SPT1/SPT2/ssSPTa and SPT1/SPT2/ssSPTb were most active with palmitoylCoA as substrate, with the latter complex also showing some activity with stearoylCoA [190]. Complexes involving SPT3 appeared more broad in substrate selectivity, with incorporation of myristoylCoA prominent for SPT1/SPT3/ssSPTa complexes, while SP1/SPT3/ssSPTb complexes had similar activity with C16, C18 and C20 acylCoAs [190].

**Table:** 

Nomenclature	serine palmitoyltransferase,	serine palmitoyltransferase,	serine palmitoyltransferase,	serine palmitoyltransferase,	serine palmitoyltransferase,
	long chain base subunit 1	long chain base subunit 2	long chain base subunit 3	small subunit A	small subunit B
Common abreviation	SPT1	SPT2	SPT3	SPTSSA	SPTSSB
HGNC, UniProt	*SPTLC1*, O15269	*SPTLC2*, O15270	*SPTLC3*, Q9NUV7	*SPTSSA*, Q969W0	*SPTSSB*, Q8NFR3
EC number	2.3.1.50: L‐serine +	2.3.1.50: L‐serine +	2.3.1.50: L‐serine +	–	–
	palmitoyl‐CoA ‐>	palmitoyl‐CoA ‐>	palmitoyl‐CoA ‐>		
	3‐ketosphinganine +	3‐ketosphinganine +	3‐ketosphinganine +		
	coenzyme A + CO_2_	coenzyme A CO_2_ +	coenzyme A CO_2_ +		
Cofactors	pyridoxal phosphate	pyridoxal phosphate	pyridoxal phosphate	–	–
Selective inhibitors	myriocin [334]	myriocin [334]	myriocin [334]	–	–

## Ceramide synthase

### Overview

This family of enzymes, also known as sphingosine *N*‐acyltransferase, is located in the ER facing the cytosol with an as‐yet undefined topology and stoichiometry. Ceramide synthase *in vitro* is sensitive to inhibition by the fungal derived toxin, fumonisin B1.

**Table:** 

Nomenclature	ceramide synthase 1	ceramide synthase 2	ceramide synthase 3
Common abreviation	CERS1	CERS2	CERS3
HGNC, UniProt	*CERS1*, P27544	*CERS2*, Q96G23	*CERS3*, Q8IU89
EC number	2.3.1.24: acylCoA + sphinganine ‐> dihydroceramide + coenzyme A sphingosine + acylCoA ‐> ceramide + coenzyme A	2.3.1.24: acylCoA + sphinganine ‐> dihydroceramide + coenzyme A sphingosine + acylCoA ‐> ceramide + coenzyme A	2.3.1.24: acylCoA + sphinganine ‐> dihydroceramide + coenzyme A sphingosine + acylCoA ‐> ceramide + coenzyme A
Substrates	C18‐CoA [499]	C24‐ and C26‐CoA [278]	C26‐CoA and longer [337, 396]

**Table:** 

Nomenclature	ceramide synthase 4	ceramide synthase 5	ceramide synthase 6
Common abreviation	CERS4	CERS5	CERS6
HGNC, UniProt	*CERS4*, Q9HA82	*CERS5*, Q8N5B7	*CERS6*, Q6ZMG9
EC number	2.3.1.24: acylCoA + sphinganine ‐> dihydroceramide + coenzyme A sphingosine + acylCoA ‐> ceramide + coenzyme A	2.3.1.24: acylCoA + sphinganine ‐> dihydroceramide + coenzyme A sphingosine + acylCoA ‐> ceramide + coenzyme A	2.3.1.24: acylCoA + sphinganine ‐> dihydroceramide + coenzyme A sphingosine + acylCoA ‐> ceramide + coenzyme A
Substrates	C18‐, C20‐ and C22‐CoA [405]	C16‐CoA [274, 405]	C14‐ and C16‐CoA [336]

## Sphingolipid Δ^4^‐desaturase

### Overview

DEGS1 and DEGS2 are 4TM proteins.

**Table:** 

Nomenclature	delta(4)‐desaturase, sphingolipid 1	delta(4)‐desaturase, sphingolipid 2
HGNC, UniProt	*DEGS1*, O15121	*DEGS2*, Q6QHC5
EC number	1.14.‐.‐	1.14.‐.‐
Cofactors	NAD	NAD
Comments	Myristoylation of DEGS1 enhances its activity and targets it to the mitochondria [26].	–

### Comments

DEGS1 activity is inhibited by a number of natural products, including curcumin and Δ^9^‐tetrahydrocannabinol[130].

## Sphingomyelin synthase

### Overview

Following translocation from the ER to the Golgi under the influence of the ceramide transfer protein, sphingomyelin synthases allow the formation of sphingomyelin by the transfer of phosphocholine from the phospholipid phosphatidylcholine.

Sphingomyelin synthase‐related protein 1 is structurally related but lacks sphingomyelin synthase activity.

**Table:** 

Nomenclature	sphingomyelin synthase 1	sphingomyelin synthase 2	sterile alpha motif domain containing 8
HGNC, UniProt	*SGMS1*, Q86VZ5	*SGMS2*, Q8NHU3	*SAMD8*, Q96LT4
EC number	2.7.8.27: ceramide + phosphatidylcholine ‐> sphingomyelin + diacylglycerol	2.7.8.27: ceramide + phosphatidylcholine ‐> sphingomyelin + diacylglycerol	2.7.8.‐: ceramide + phosphatidylethanolamine ‐> ceramide phosphoethanolamine
Comments	–	Palmitoylation of sphingomyelin synthase 2 may allow targeting to the plasma membrane [476].	–

## Sphingomyelin phosphodiesterase

### Overview

Also known as sphingomyelinase.

**Table:** 

Nomenclature	sphingomyelin phosphodiesterase 1,	sphingomyelin phosphodiesterase 2,	sphingomyelin phosphodiesterase 3,
	acid lysosomal	neutral membrane (neutral sphingomyelinase)	neutral membrane (neutral sphingomyelinase II)
HGNC, UniProt	*SMPD1*, P17405	*SMPD2*, O60906	*SMPD3*, Q9NY59
EC number	3.1.4.12: sphingomyelin ‐> ceramide + phosphocholine	3.1.4.12: sphingomyelin ‐> ceramide + phosphocholine	3.1.4.12: sphingomyelin ‐> ceramide + phosphocholine

**Table:** 

Nomenclature	sphingomyelin phosphodiesterase 4, neutral	sphingomyelin phosphodiesterase, acid‐like 3A	sphingomyelin phosphodiesterase, acid‐like 3B
	membrane (neutral sphingomyelinase‐3)		
HGNC, UniProt	*SMPD4*, Q9NXE4	*SMPDL3A*, Q92484	*SMPDL3B*, Q92485
EC number	3.1.4.12: sphingomyelin ‐> ceramide + phosphocholine	3.1.4.‐: sphingomyelin ‐> ceramide + phosphocholine	3.1.4.‐: sphingomyelin ‐> ceramide + phosphocholine

## Neutral sphingomyelinase coupling factors

### Overview

Protein FAN [2] and polycomb protein EED [383] allow coupling between TNF receptors and neutral sphingomyelinase phosphodiesterases.

**Table:** 

Nomenclature	embryonic ectoderm development	neutral sphingomyelinase (N‐SMase) activation associated factor
HGNC, UniProt	*EED*, O75530	*NSMAF*, Q92636

## Ceramide glucosyltransferase

**Table:** 

Nomenclature	UDP‐glucose ceramide glucosyltransferase
HGNC, UniProt	*UGCG*, Q16739
EC number	2.4.1.80: UDP‐glucose + ceramide = uridine diphosphate + glucosylceramide
Inhibitors	miglustat (p*K*_i_ 5.1) [53]
Comments	Glycoceramides are an extended family of sphingolipids, differing in the content and organization of the sugar moieties, as well as the acyl sidechains.

## Acid ceramidase

### Overview

The six human ceramidases may be divided on the basis of pH optimae into acid, neutral and alkaline ceramidases, which also differ in their subcellular location.

**Table:** 

Nomenclature	N‐acylsphingosine amidohydrolase (acid ceramidase) 1
HGNC, UniProt	*ASAH1*, Q13510
EC number	3.5.1.23: ceramide ‐>sphingosine + a fatty acid
Comments	This lysosomal enzyme is proteolysed to form the mature protein made up of two chains from the same gene product [261].

## Neutral ceramidases

### Overview

The six human ceramidases may be divided on the basis of pH optimae into acid, neutral and alkaline ceramidases, which also differ in their subcellular location.

**Table:** 

Nomenclature	N‐acylsphingosine amidohydrolase (non‐lysosomal ceramidase) 2	N‐acylsphingosine amidohydrolase (non‐lysosomal ceramidase) 2B
HGNC, UniProt	*ASAH2*, Q9NR71	*ASAH2B*, P0C7U1
EC number	3.5.1.23: ceramide ‐>sphingosine + a fatty acid	–
Comments	The enzyme is associated with the plasma membrane [475].	–

### Comments

ASAH2B appears to be an enzymatically inactive protein, which may result from gene duplication and truncation.

## Alkaline ceramidases

### Overview

The six human ceramidases may be divided on the basis of pH optimae into acid, neutral and alkaline ceramidases, which also differ in their subcellular location.

**Table:** 

Nomenclature	alkaline ceramidase 1	alkaline ceramidase 2	alkaline ceramidase 3
HGNC, UniProt	*ACER1*, Q8TDN7	*ACER2*, Q5QJU3	*ACER3*, Q9NUN7
EC number	3.5.1.23: ceramide ‐>sphingosine + a fatty acid	3.5.1.23: ceramide ‐>sphingosine + a fatty acid	3.5.1.‐
Comments	ACER1 is associated with the ER [466].	ACER2 is associated with the Golgi apparatus [534].	ACER3 is associated with the ER and Golgi apparatus [314].

## Ceramide kinase

**Table:** 

Nomenclature	ceramide kinase
HGNC, UniProt	*CERK*, Q8TCT0
EC number	2.7.1.138: ceramide + ATP ‐> ceramide 1‐phosphate + adenosine diphosphate
Inhibitors	NVP 231 (pIC_50_ 7.9) [178]

### Comments

A ceramide kinase‐like protein has been identified in the human genome (*CERKL*, Q49MI3).

### Further Reading

Castro BM *et al*. (2014) Ceramide: a simple sphingolipid with unique biophysical properties. *Prog. Lipid Res.* **54**: 53‐67 [PMID:24513486]

Halmer R *et al*. (2014) Sphingolipids: important players in multiple sclerosis. *Cell. Physiol. Biochem.* **34**: 111‐8 [PMID:24977485]

Ito M *et al*. (2014) New insight into the structure, reaction mechanism, and biological functions of neutral ceramidase. *Biochim. Biophys. Acta* **1841**: 682‐91 [PMID:24064302]

Khavandgar Z *et al*. (2015) Sphingolipid metabolism and its role in the skeletal tissues. *Cell. Mol. Life Sci.* **72**: 959‐69 [PMID:25424644]

Lowther J *et al*. (2012) Structural, mechanistic and regulatory studies of serine palmitoyltransferase. *Biochem. Soc. Trans.* **40**: 547‐54 [PMID:22616865]

Saied EM *et al*. (2014) Small molecule inhibitors of ceramidases. *Cell. Physiol. Biochem.* **34**: 197‐212 [PMID:24977492]

## Chromatin modifying enzymes

### Overview

Chromatin modifying enzymes, and other chromatin‐modifying proteins, fall into three broad categories: **writers**, **readers** and **erasers**. The function of these proteins is to dynamically maintain cell identity and regulate processes such as differentiation, development, proliferation and genome integrity *via* recognition of specific 'marks' (covalent post‐translational modifications) on histone proteins and DNA [267]. In normal cells, tissues and organs, precise co‐ordination of these proteins ensures expression of only those genes required to specify phenotype or which are required at specific times, for specific functions. Chromatin modifications allow DNA modifications not coded by the DNA sequence to be passed on through the genome and underlies heritable phenomena such as X chromosome inactivation, aging, heterochromatin formation, reprogramming, and gene silencing (epigenetic control).

To date at least eight distinct types of modifications are found on histones. These include small covalent modifications such as acetylation, methylation, and phosphorylation, the attachment of larger modifiers such as ubiquitination or sumoylation, and ADP ribosylation, proline isomerization and deimination. Chromatin modifications and the functions they regulate in cells are reviewed by Kouzarides (2007) [267].

**Writer** proteins include the histone methyltransferases, histone acetyltransferases, some kinases and ubiquitin ligases.

**Readers** include proteins which contain methyl‐lysine‐recognition motifs such as bromodomains, chromodomains, tudor domains, PHD zinc fingers, PWWP domains and MBT domains.

**Erasers** include the histone demethylases and histone deacetylases (HDACs and sirtuins).

Dysregulated epigenetic control can be associated with human diseases such as cancer [129], where a wide variety of cellular and protein abberations are known to perturb chromatin structure, gene transcription and ultimately cellular pathways [24, 439]. Due to the reversible nature of epigenetic modifications, chromatin regulators are very tractable targets for drug discovery and the development of novel therapeutics. Indeed, small molecule inhibitors of writers (*e.g.* azacitidine and decitabine target the DNA methyltransferases DNMT1 and DNMT3 for the treatment of myelodysplastic syndromes [165, 520]) and erasers (*e.g.* the HDAC inhibitors vorinostat, romidepsin and belinostat for the treatment of T‐cell lymphomas [144, 254]) are already being used in the clinic. The search for the next generation of compounds with improved specificity against chromatin‐associated proteins is an area of intense basic and clinical research [56]. Current progress in this field is reviewed by Simó‐Riudalbas and Esteller (2015) [440].

## 2.1.1.‐ Protein arginine N‐methyltransferases

### Overview

Protein arginine N‐methyltransferases (PRMT, EC 2.1.1.‐) encompass histone arginine *N*‐methyltransferases (PRMT4, PRMT7, EC 2.1.1.125) and myelin basic protein *N*‐methyltransferases (PRMT7, EC 2.1.1.126). They are dimeric or tetrameric enzymes which use S‐adenosyl methionine as a methyl donor, generating S‐adenosylhomocysteine as a by‐product. They generate both mono‐methylated and di‐methylated products; these may be symmetric (SDMA) or asymmetric (N^G^,N^G^‐dimethyl‐L‐arginine) versions, where both guanidine nitrogens are monomethylated or one of the two is dimethylated, respectively.

## 3.5.1.‐ Histone deacetylases (HDACs)

### Overview

Histone deacetylases act as erasers of epigenetic acetylation marks on lysine residues in histones. Removal of the acetyl groups facilitates tighter packing of chromatin (heterochromatin formation) leading to transcriptional repression.

The histone deacetylase family has been classified in to five subfamilies based on phylogenetic comparison with yeast homologues:

Class I contains HDACs 1, 2, 3 and 8

Class IIa contains HDACs 4, 5, 7 and 9

Class IIb contains HDACs 6 and 10

Class III contains the sirtuins (SIRT1‐7)

Class IV contains only HDAC11.

Classes I, II and IV use Zn^+^ as a co‐factor, whereas catalysis by Class III enzymes requires NAD^+^ as a co‐factor, and members of this subfamily have ADP‐ribosylase activity in addition to protein deacetylase function [420].

HDACs have more general protein deacetylase activity, being able to deacetylate lysine residues in non‐histone proteins [82] such as microtubules [221], the hsp90 chaperone [268] and the tumour suppressor p53 [302].

Dysregulated HDAC activity has been identified in cancer cells and tumour tissues [288, 410], making HDACs attractive molecular targets in the search for novel mechanisms to treat cancer [522]. Several small molecule HDAC inhibitors are already approved for clinical use: romidepsin, belinostat, vorinostat, panobinostat, belinostat, valproic acid and chidamide. HDACs and HDAC inhibitors currently in development as potential anti‐cancer therapeutics are reviewed by Simó‐Riudalbas and Esteller (2015) [440].

## Cyclic nucleotide turnover

### Overview

Cyclic nucleotides are second messengers generated by cyclase enzymes from precursor triphosphates and hydrolysed by phosphodiesterases. The cellular actions of these cyclic nucleotides are mediated through activation of protein kinases (cAMP‐ and cGMP‐dependent protein kinases), ion channels (cyclic nucleotide‐gated, CNG, and hyperpolarization and cyclic nucleotide‐gated, HCN) and guanine nucleotide exchange factors (GEFs, Epac).

## Adenylyl cyclases

### Overview

Adenylyl cyclase, E.C. 4.6.1.1, converts ATP to cyclic AMP and pyrophosphate. Mammalian membrane‐bound adenylyl cyclases are typically made up of two clusters of six TM domains separating two intracellular, overlapping catalytic domains that are the target for the nonselective activators forskolin, NKH477(except AC9, [392]) and G*α*_s_(the stimulatory G protein *α* subunit). Adenosine and its derivatives (*e.g.* 2',5'‐dideoxyadenosine), acting through the P‐site, appear to be physiological inhibitors of adenylyl cyclase activity [485]. Three families of adenylyl cyclase are distinguishable: calmodulin(*CALM1* *CALM2* *CALM3*, P62158)‐stimulated (AC1, AC3 and AC8), Ca^2+^‐inhibitable (AC5, AC6 and AC9) and Ca^2+^‐insensitive (AC2, AC4 and AC7) forms.

**Table:** 

Nomenclature	AC1	AC2	AC3	AC4	AC5
HGNC, UniProt	*ADCY1*, Q08828	*ADCY2*, Q08462	*ADCY3*, O60266	*ADCY4*, Q8NFM4	*ADCY5*, O95622
EC number	4.6.1.1	4.6.1.1	4.6.1.1	4.6.1.1	4.6.1.1
Endogenous activators	calmodulin (*CALM1* *CALM2* *CALM3*, P62158), PKC‐evoked phosphorylation [233, 474]	G*β* *γ*, PKC‐evoked phosphorylation [73, 306, 478]	calmodulin (*CALM1* *CALM2* *CALM3*, P62158), PKC‐evoked phosphorylation [81, 233]	G*β* *γ* [163]	PKC‐evoked phophorylation [250]
Endogenous inhibitors	G*α*_i_, G*α*_o_, G*β* *γ* [478, 479]	–	G*α*_i_, RGS2, CaM kinase II‐evoked phosphorylation [441, 479, 517]	PKC‐evoked phophorylation [554]	G*α*_i_, Ca^2+^, PKA‐evoked phosphorylation [227, 230, 479]
Selective inhibitors	–	–	–	–	NKY80 [364]

**Table:** 

Nomenclature	AC6	AC7	AC8	AC9
HGNC, UniProt	*ADCY6*, O43306	*ADCY7*, P51828	*ADCY8*, P40145	*ADCY9*, O60503
EC number	4.6.1.1	4.6.1.1	4.6.1.1	4.6.1.1
Endogenous activators	–	PKC‐evoked phosphorylation [516]	Ca^2+^ [57]	–
Endogenous inhibitors	G*α*_i_, Ca^2+^, PKA‐evoked phosphorylation, PKC‐evoked phosphorylation [76, 275, 479, 541]	–	–	Ca^2+^/calcineurin [376]

### Comments

Nitric oxide has been proposed to inhibit AC5 and AC6 selectively [210], although it is unclear whether this phenomenon is of physiological significance. A soluble adenylyl cyclase has been described (*ADCY10*, Q96PN6 [48]), unaffected by either G*α* or G*β* *γ* subunits, which has been suggested to be a cytoplasmic bicarbonate (pH‐insensitive) sensor [75]. It can be inhibited selectively by KH7 (pIC_50_ 5.0‐5.5) [208].

## Soluble guanylyl cyclase

### Overview

Soluble guanylyl cyclase (GTP diphosphate‐lyase (cyclising)), E.C. 4.6.1.2, is a heterodimer comprising *α* and *β* chains, both of which have two subtypes in man (predominantly *α*1*β*1; [544]). A haem group is associated with the *β* chain and is the target for the endogenous ligand nitric oxide (NO∙), and, potentially, carbon monoxide [150]. The enzyme converts guanosine‐5'‐triphosphate to the intracellular second messenger 3',5'‐guanosine monophosphate (cyclic GMP).

**Table:** 

Nomenclature	Soluble guanylyl cyclase
Common abreviation	sGC
Subunits	Soluble guanylyl cyclase *β* 1 subunit, Soluble guanylyl cyclase *α* 1 subunit
EC number	4.6.1.2
Activators	isosorbide dinitrate, isosorbide mononitrate, nitroglycerin
Selective activators	BAY412272 [460], NO, YC1 [150], ataciguat [421], cinaciguat [461], riociguat [461]
Inhibitors	NS 2028 (pIC_50_ 8.1) [363] – Bovine
Selective inhibitors	ODQ (pIC_50_ 7.5) [167]

### Subunits

**Table:** 

Nomenclature	*α* 1 subunit	*α* 2 subunit	*β* 1 subunit	*β* 2 subunit
HGNC, UniProt	*GUCY1A3*, Q02108	*GUCY1A2*, P33402	*GUCY1B3*, Q02153	*GUCY1B2*, O75343

### Comments

ODQ also shows activity at other haem‐containing proteins [134], while YC1 may also inhibit cGMP‐hydrolysing phosphodiesterases [149, 159].

## Exchange protein activated by cyclic AMP (Epac)

### Overview

Epacs are members of a family of guanine nucleotide exchange factors (ENSFM00250000000899), which also includes *RapGEF5*(GFR, KIAA0277, MR‐GEF, Q92565) and *RapGEFL1* (Link‐GEFII, Q9UHV5). They are activated endogenously by cyclic AMP and with some pharmacological selectivity by 8‐pCPT‐2'‐O‐Me‐cAMP[126]. Once activated, Epacs induce an enhanced activity of the monomeric G proteins, Rap1 and Rap2 by facilitating binding of guanosine‐5'‐triphosphate in place of guanosine 5'‐diphosphate, leading to activation of phospholipase C [423].

**Table:** 

Nomenclature	Epac1	Epac2
HGNC, UniProt	*RAPGEF3*, O95398	*RAPGEF4*, Q8WZA2
Inhibitors	–	HJC 0350 (pIC_50_ 6.5) [72], ESI‐09 (pIC_50_ 4.4) [11]

## Phosphodiesterases, 3',5'‐cyclic nucleotide

### Overview

3',5'‐Cyclic nucleotide phosphodiesterases (PDEs, 3',5'‐cyclic‐nucleotide 5'‐nucleotidohydrolase), E.C. 3.1.4.17, catalyse the hydrolysis of a 3',5'‐cyclic nucleotide (usually cyclic AMP or cyclic GMP). Isobutylmethylxanthine is a nonselective inhibitor with an IC_50_ value in the millimolar range for all isoforms except PDE 8A, 8B and 9A. A 2',3'‐cyclic nucleotide 3'‐phosphodiesterase (E.C. 3.1.4.37 CNPase) activity is associated with myelin formation in the development of the CNS.

**Table:** 

Nomenclature	PDE1A	PDE1B	PDE1C
HGNC, UniProt	*PDE1A*, P54750	*PDE1B*, Q01064	*PDE1C*, Q14123
EC number	3.1.4.17	3.1.4.17	3.1.4.17
Rank order of affinity	cyclic GMP>cyclic AMP	cyclic GMP>cyclic AMP	cyclic GMP = cyclic AMP
Endogenous activators	calmodulin (*CALM1* *CALM2* *CALM3*, P62158)	calmodulin (*CALM1* *CALM2* *CALM3*, P62158)	calmodulin (*CALM1* *CALM2* *CALM3*, P62158)
Selective inhibitors	SCH51866 (pIC_50_ 7.2) [498], vinpocetine (pIC_50_ 5.1) [300]	SCH51866 (pIC_50_ 7.2) [498]	SCH51866 (pIC_50_ 7.2) [498], vinpocetine (pIC_50_ 4.3) [300]
Comments	–	–	–

**Table:** 

Nomenclature	PDE2A	PDE3A	PDE3B
HGNC, UniProt	*PDE2A*, O00408	*PDE3A*, Q14432	*PDE3B*, Q13370
EC number	3.1.4.17	3.1.4.17	3.1.4.17
Rank order of affinity	cyclic AMP≫cyclic GMP	–	–
Endogenous activators	cyclic GMP	–	–
Endogenous inhibitors	–	cyclic GMP (Selective)	cyclic GMP (Selective)
Inhibitors	milrinone (pIC_50_<6.5) [465]	cilostazol (pIC_50_ 6.7) [465], inamrinone (pIC_50_ 4.8) [442]	–
Selective inhibitors	BAY607550 (pIC_50_ 8.3–8.8) [44], EHNA (pIC_50_ 5.3) [332]	cilostamide (pIC_50_ 7.5) [465], anagrelide (pIC_50_ 7.1–7.3) [245, 319, 326], milrinone (pIC_50_ 6.3–6.4) [123, 465]	cilostamide (pIC_50_ 7.3) [465], cilostazol (pIC_50_ 6.4) [465], milrinone (pIC_50_ 6) [465], inamrinone (pIC_50_ 4.5) [465]
Comments	EHNA is also an inhibitor of adenosine deaminase (E.C. 3.5.4.4).	–	–

**Table:** 

Nomenclature	PDE4A	PDE4B	PDE4C	PDE4D	PDE5A
HGNC, UniProt	*PDE4A*, P27815	*PDE4B*, Q07343	*PDE4C*, Q08493	*PDE4D*, Q08499	*PDE5A*, O76074
EC number	3.1.4.17	3.1.4.17	3.1.4.17	3.1.4.17	3.1.4.17
Activator	–	–	–	PKA‐mediated phosphorylation [217]	Protein kinase A, protein kinase G [93]
Rank order of affinity	cyclic AMP≫cyclic GMP	cyclic AMP≫cyclic GMP	cyclic AMP≫cyclic GMP	cyclic AMP≫cyclic GMP	cyclic GMP>cyclic AMP
Inhibitors	ibudilast (pIC_50_ 7.3) [262], RS‐25344 (pIC_50_ 7.2) [417]	roflumilast (pIC_50_ 9.4) [301], ibudilast (pIC_50_ 7.2) [262], RS‐25344 (pIC_50_ 6.5) [417]	RS‐25344 (pIC_50_ 8.1) [417], ibudilast (pIC_50_ 6.6) [262]	RS‐25344 (pIC_50_ 8.4) [417]	gisadenafil (pIC_50_ 8.9) [402], milrinone (pIC_50_ 7.3)
(Sub)family‐selective inhibitors	rolipram (pIC_50_ 9) [508], Ro20‐1724 (pIC_50_ 6.5) [508]	rolipram (pIC_50_ 9) [508], Ro20‐1724 (pIC_50_ 6.4) [508]	rolipram (pIC_50_ 6.5) [508], Ro20‐1724 (pIC_50_ 5.4) [508]	rolipram (pIC_50_ 7.2) [508], Ro20‐1724 (pIC_50_ 6.2) [508]	–
Selective inhibitors	YM976 (pIC_50_ 8.3) [13]	–	–	–	vardenafil (pIC_50_ 9.7) [47], T0156 (pIC_50_ 9.5) [338], sildenafil (pIC_50_ 9) [494], tadalafil (pIC_50_ 8.5) [339], SCH51866 (pIC_50_ 7.2) [498], zaprinast (pIC_50_ 6.8) [494]

**Table:** 

Nomenclature	PDE6A	PDE6B	PDE6C	PDE6D	PDE6G	PDE6H
HGNC, UniProt	*PDE6A*, P16499	*PDE6B*, P35913	*PDE6C*, P51160	*PDE6D*, O43924	*PDE6G*, P18545	*PDE6H*, Q13956
EC number	3.1.4.17	3.1.4.17	3.1.4.17	3.1.4.17	3.1.4.17	3.1.4.17
Activators	–	–	–	–	–	–
Rank order of affinity	–	–	–	–	–	–
Inhibitors	–	–	–	–	–	–
Selective inhibitors	–	–	–	–	–	–

**Table:** 

Nomenclature	PDE7A	PDE7B	PDE8A	PDE8B
HGNC, UniProt	*PDE7A*, Q13946	*PDE7B*, Q9NP56	*PDE8A*, O60658	*PDE8B*, O95263
EC number	3.1.4.17	3.1.4.17	3.1.4.17	3.1.4.17
Rank order of affinity	cyclic AMP≫cyclic GMP [330]	cyclic AMP≫cyclic GMP [166]	cyclic AMP≫cyclic GMP [138]	cyclic AMP≫cyclic GMP [201]
Inhibitors	–	BRL50481 (pIC_50_ 4.9) [7]	–	–
Selective inhibitors	BRL50481 (pIC_50_ 6.7–6.8) [7, 448]	dipyridamole (pIC_50_ 5.7–6) [166, 419], SCH51866 (pIC_50_ 5.8) [419]	dipyridamole (pIC_50_ 5.1) [138]	dipyridamole (pIC_50_ 4.3) [201]
Comments	–	–	PDE7A appears to be membrane‐bound or soluble for PDE7A1 and 7A2 splice variants, respectively	–

**Table:** 

Nomenclature	PDE9A	PDE10A	PDE11A
HGNC, UniProt	*PDE9A*, O76083	*PDE10A*, Q9Y233	*PDE11A*, Q9HCR9
EC number	3.1.4.17	3.1.4.17	3.1.4.17
Rank order of affinity	cyclic GMP≫cyclic AMP [137]	cyclic AMP, cyclic GMP [152]	cyclic AMP, cyclic GMP [133]
Inhibitors	–	–	tadalafil (pIC_50_ 6.5) [339], BC11‐38 (pIC_50_ 6.5) [68]
Selective inhibitors	SCH51866 (pIC_50_ 5.8) [137], zaprinast (pIC_50_ 4.5) [137]	–	–
Comments	–		

### Comments

PDE1A, 1B and 1C appear to act as soluble homodimers, while PDE2A is a membrane‐bound homodimer. PDE3A and PDE3B are membrane‐bound.

PDE4 isoforms are essentially cyclic AMP specific. The potency of YM976 at other members of the PDE4 family has not been reported. PDE4B‐D long forms are inhibited by extracellular signal‐regulated kinase (ERK)‐mediated phosphorylation [212, 213]. PDE4A‐D splice variants can be membrane‐bound or cytosolic [217]. PDE4 isoforms may be labelled with [^3^H]rolipram.

PDE6 is a membrane‐bound tetramer composed of two catalytic chains (PDE6A or PDE6C and PDE6B), an inhibitory chain (PDE6G or PDE6H) and the PDE6D chain. The enzyme is essentially cyclic GMP specific and is activated by the *α*‐subunit of transducin (G*α*_t_) and inhibited by sildenafil, zaprinast and dipyridamole with potencies lower than those observed for PDE5A. Defects in PDE6B are a cause of retinitis pigmentosa and congenital stationary night blindness.

### Further Reading

Cooper DM *et al*. (2014) Adenylate cyclase‐centred microdomains. *Biochem. J.* **462**: 199‐213 [PMID:25102028]

Derbyshire ER *et al*. (2012) Structure and regulation of soluble guanylate cyclase. *Annu. Rev. Biochem.* **81**: 533‐59 [PMID:22404633]

Francis SH *et al*. (2011) Mammalian cyclic nucleotide phosphodiesterases: molecular mechanisms and physiological functions. *Physiol. Rev.* **91**: 651‐90 [PMID:21527734]

Nicol X *et al*. (2014) Routes to cAMP: shaping neuronal connectivity with distinct adenylate cyclases. *Eur. J. Neurosci.* **39**: 1742‐51 [PMID:24628976]

Schmidt M *et al*. (2013) Exchange protein directly activated by cAMP (epac): a multidomain cAMP mediator in the regulation of diverse biological functions. *Pharmacol. Rev.* **65**: 670‐709 [PMID:23447132]

Steegborn C. (2014) Structure, mechanism, and regulation of soluble adenylyl cyclases ‐ similarities and differences to transmembrane adenylyl cyclases. *Biochim. Biophys. Acta* **1842**: 2535‐47 [PMID:25193033]

## Cytochrome P450

### Overview

The cytochrome P450 enzyme family (CYP450), E.C. 1.14.‐.‐, were originally defined by their strong absorbance at 450 nm due to the reduced carbon monoxide‐complexed haem component of the cytochromes. They are an extensive family of haem‐containing monooxygenases with a huge range of both endogenous and exogenous substrates. Listed below are the human enzymes; their relationship with rodent CYP450 enzyme activities is obscure in that the species orthologue may not mediate metabolism of the same substrates. Although the majority of CYP450 enzyme activities are concentrated in the liver, the extrahepatic enzyme activities also contribute to patho/physiological processes. Genetic variation of CYP450 isoforms is widespread and likely underlies a significant proportion of the individual variation to drug administration. Further family members are included on the online database at www.GuidetoPHARMACOLOGY.org

## CYP1 family

**Table:** 

Nomenclature	CYP1A1	CYP1A2	CYP1B1
HGNC, UniProt	*CYP1A1*, P04798	*CYP1A2*, P05177	*CYP1B1*, Q16678
EC number	1.14.1.1	1.14.1.1	1.14.1.1
Comments	–	–	Mutations have been associated with primary congenitial glucoma [464]

## CYP2 family

**Table:** 

Nomenclature	CYP2A6	CYP2A7	CYP2C8
HGNC, UniProt	*CYP2A6*, P11509	*CYP2A7*, P20853	*CYP2C8*, P10632
EC number	1.14.14.1	1.14.14.1	1.14.14.1
Comments	Metabolises nicotine	CYP2A7 does not incorporate haem and is functionally inactive [153]	Converts arachidonic acid to 11(R)‐12(S)‐epoxyeicosatrienoic acid or 14(R)‐15(S)‐epoxyeicosatrienoic acid [547].

**Table:** 

Nomenclature	CYP2J2	CYP2R1
HGNC, UniProt	*CYP2J2*, P51589	*CYP2R1*, Q6VVX0
EC number	1.14.14.1	1.14.13.15
Comments	Converts arachidonic acid to 14(R)‐15(S)‐epoxyeicosatrienoic acid [531].	Converts vitamin D3 to 25‐hydroxyvitamin D_3_ [78].

## CYP3 family

**Table:** 

Nomenclature	CYP3A4
HGNC, UniProt	*CYP3A4*, P08684
EC number	1.14.13.32: Albendazole + NADPH + O_2_ = albendazole S‐oxide + NADP^+^ + H_2_ 1.14.13.157: 1,8‐cineole + NADPH + O_2_ = 2‐exo‐hydroxy‐1,8‐cineole + NADP^+^ + H_2_O 1.14.13.97: Taurochenodeoxycholate + NADPH + O_2_ = taurohyocholate + NADP^+^ + H_2_O Lithocholate + NADPH + O_2_ = hyodeoxycholate + NADP^+^ + H_2_O 1.14.13.67: quinine + NADPH + O_2_ = 3‐hydroxyquinine + NADP^+^ + H_2_O_2_
Substrates	atorvastatin [140], codeine [140], diazepam [140], tamoxifen [140], erlotinib [140]
Products	4‐hydroxy‐tamoxifen quinone methide [432], 4‐hydroxy‐tamoxifen [432]
Comments	Metabolises a vast range of xenobiotics, including antidepressants, benzodiazepines, calcium channel blockers, and chemotherapeutic agents. CYP3A4 catalyses the 25‐hydroxylation of trihydroxycholestane in liver microsomes [157].

## CYP4 family

**Table:** 

Nomenclature	CYP4A11	CYP4F2	CYP4F3	CYP4F8	CYP4F12
HGNC, UniProt	*CYP4A11*, Q02928	*CYP4F2*, P78329	*CYP4F3*, Q08477	*CYP4F8*, P98187	*CYP4F12*, Q9HCS2
EC number	1.14.15.3	1.14.13.30	1.14.13.30	1.14.14.1	1.14.14.1
Comments	Converts lauric acid to 12‐hydroxylauric acid.	Responsible for *ω*‐hydroxylation of LTB_4_, LXB_4_ [335], and tocopherols, including vitamin E [453]	Responsible for *ω*‐hydroxylation of LTB_4_, LXB_4_ [335], and polyunsaturated fatty acids [135, 194]	Converts PGH_2_ to 19‐hydroxyPGH_2_ [54] and 8,9‐EET or 11,12‐EET to 18‐hydroxy‐8,9‐EET or 18‐hydroxy‐11,12‐EET [353].	AC004597.1 (ENSG00000225607) is described as being highly similar to CYP4F12

**Table:** 

Nomenclature	CYP4F22	CYP4V2	CYP4X1	CYP4Z1
HGNC, UniProt	*CYP4F22*, Q6NT55	*CYP4V2*, Q6ZWL3	*CYP4X1*, Q8N118	*CYP4Z1*, Q86W10
EC number	1.14.14.‐	1.14.‐.‐	1.14.14.1	1.14.14.1
Comments	Converts arachidonic acid to 16‐HETE and 18‐HETE [353].	Converts myristic acid to 14‐hydroxymyristic acid [348].	Converts anandamide to 14,15‐epoxyeicosatrienoic ethanolamide [459].	Converts lauric acid to 12‐hydroxylauric acid

## CYP5, CYP7 and CYP8 families

**Table:** 

Nomenclature	CYP5A1	CYP8A1	CYP7A1	CYP7B1	CYP8B1
Common name	–	Prostacyclin synthase	–	–	–
HGNC, UniProt	*TBXAS1*, P24557	*PTGIS*, Q16647	*CYP7A1*, P22680	*CYP7B1*, O75881	*CYP8B1*, Q9UNU6
EC number	5.3.99.5: PGH_2_ = thromboxane A_2_	5.3.99.4	1.14.13.17	1.14.13.100	1.14.13.95
Comments	Inhibited by dazoxiben [398] and camonagrel [182].	Converts PGH_2_ to PGI_2_ [196]. Inhibited by tranylcypromine [181]	Converts cholesterol to 7*α*‐hydroxycholesterol [354].	Converts dehydroepiandrosterone to 7*α*‐DHEA [411].	Converts 7*α*‐hydroxycholest‐4‐en‐3‐one to 7‐alpha,12*α*‐dihydroxycholest‐4‐en‐3‐one (in rabbit) [226] in the biosynthesis of bile acids.

## CYP11, CYP17, CYP19, CYP20 and CYP21 families

**Table:** 

Nomenclature	CYP11A1	CYP11B1	CYP11B2	CYP17A1
Common name	–	–	Aldosterone synthase	–
HGNC, UniProt	*CYP11A1*, P05108	*CYP11B1*, P15538	*CYP11B2*, P19099	*CYP17A1*, P05093
EC number	1.14.15.6	1.14.15.4	1.14.15.4	1.14.99.9
			1.14.15.5	
Inhibitors	mitotane	metyrapone (pIC_50_ 7.8) [553], mitotane	osilodrostat (pIC_50_ 9.7) [537]	abiraterone (pIC_50_ 7.1–8.4) [386, 390]
Selective inhibitors	–	–	–	galeterone (pIC_50_ 6.5) [192]
Comments	Converts cholesterol to pregnenolone plus 4‐methylpentanal.	Converts deoxycortisone and 11‐deoxycortisol to cortisone and cortisol, respectively. Loss‐of‐function mutations are associated with familial adrenal hyperplasia and hypertension. Inhibited by metyrapone [513]	Converts corticosterone to aldosterone	Converts pregnenolone and progesterone to 17*α*‐hydroxypregnenolone and 17*α*‐hydroxyprogesterone, respectively. Converts 17*α*‐hydroxypregnenolone and 17*α*‐hydroxyprogesterone to dehydroepiandrosterone and androstenedione, respectively. Converts corticosterone to cortisol.

**Table:** 

Nomenclature	CYP19A1	CYP20A1	CYP21A2
Common name	Aromatase	–	–
HGNC, UniProt	*CYP19A1*, P11511	*CYP20A1*, Q6UW02	*CYP21A2*, P08686
EC number	1.14.14.1	1.14.‐.‐	1.14.99.10
Inhibitors	anastrozole (pIC_50_ 7.8) [343], aminoglutethimide [379]	–	–
Selective inhibitors	letrozole (p*K*_i_ 10.7) [323], exemestane (pIC_50_ 7.3) [85], testolactone (p*K*_i_ 4.5) [95]	–	–
Comments	Converts androstenedione and testosterone to estrone and 17*β*‐estradiol, respectively. Inhibited by anastrozole [388], and letrozole [33]	–	Converts progesterone and 17*α*‐hydroxyprogesterone to deoxycortisone and 11‐deoxycortisol, respectively

## CYP24, CYP26 and CYP27 families

**Table:** 

Nomenclature	CYP24A1	CYP26A1	CYP26B1	CYP27A1	CYP27B1
Common abreviation	–	–	–	Sterol 27‐hydroxylase	–
HGNC, UniProt	*CYP24A1*, Q07973	*CYP26A1*, O43174	*CYP26B1*, Q9NR63	*CYP27A1*, Q02318	*CYP27B1*, O15528
EC number	1.14.13.126	1.14.‐.‐	1.14.‐.‐	1.14.13.15	1.14.13.13
Comments	Converts 1,25‐dihydroxyvitamin D3 (calcitriol) to 1*α*,24R,25‐trihydroxyvitamin D_3_.	Converts retinoic acid to 4‐hydroxyretinoic acid. Inhibited by liarozole	Converts retinoic acid to 4‐hydroxyretinoic acid.	Converts cholesterol to 27‐hydroxycholesterol.	Converts 25‐hydroxyvitamin D_3_ to 1,25‐dihydroxyvitamin D3 (calcitriol)

## CYP39, CYP46 and CYP51 families

**Table:** 

Nomenclature	CYP39A1	CYP46A1	CYP51A1
Common abreviation	–	Cholesterol 24‐hydroxylase	Lanosterol 14‐*α*‐demethylase
HGNC, UniProt	*CYP39A1*, Q9NYL5	*CYP46A1*, Q9Y6A2	*CYP51A1*, Q16850
EC number	1.14.13.99	1.14.13.98	–
Comments	Converts 24‐hydroxycholesterol to 7*α*,24‐dihydroxycholesterol [286].	Converts cholesterol to 24(S)‐hydroxycholesterol.	Converts lanosterol to 4,4‐dimethylcholesta‐8.14.24‐trienol.

### Further Reading

Guengerich FP *et al*. (2011) Orphans in the human cytochrome P450 superfamily: approaches to discovering functions and relevance in pharmacology. *Pharmacol. Rev.* **63**: 684‐99 [PMID:21737533]

Lorbek G *et al*. (2012) Cytochrome P450s in the synthesis of cholesterol and bile acids–from mouse models to human diseases. *FEBS J.* **279**: 1516‐33 [PMID:22111624]

Orr ST *et al*. (2012) Mechanism‐based inactivation (MBI) of cytochrome P450 enzymes: structure‐activity relationships and discovery strategies to mitigate drug‐drug interaction risks. *J. Med. Chem.* **55**: 4896‐933 [PMID:22409598]

Peñas‐Lledó EM *et al*. (2014) CYP2D6 variation, behaviour and psychopathology: implications for pharmacogenomics‐guided clinical trials. *Br J Clin Pharmacol* **77**: 673‐83 [PMID:24033670]

Ross AC *et al*. (2011) Cytochrome P450s in the regulation of cellular retinoic acid metabolism. *Annu. Rev. Nutr.* **31**: 65‐87 [PMID:21529158]

Shahabi P *et al*. (2014) Human cytochrome P450 epoxygenases: variability in expression and role in inflammation‐related disorders. *Pharmacol. Ther.* **144**: 134‐61 [PMID:24882266]

Werk AN *et al*. (2014) Functional gene variants of CYP3A4. *Clin. Pharmacol. Ther.* **96**: 340‐8 [PMID:24926778]

## Endocannabinoid turnover

### Overview

The principle endocannabinoids are 2‐arachidonoylglycerol(2AG) and anandamide(N‐arachidonoylethanolamine, AEA), thought to be generated on demand rather than stored, although this may not always be the case [10]. Mechanisms for release and re‐uptake of endocannabinoids (and related entities) are unclear, although candidates for intracellular transport have been suggested. For the generation of 2‐arachidonoylglycerol, the key enzyme involved is diacylglycerol lipase (DGL), whilst several routes for anandamide synthesis have been described, the best characterized of which involves *N*‐acylphosphatidylethanolamine‐phospholipase D (NAPE‐PLD, [438]). Inactivation of these endocannabinoids appears to occur predominantly through monoacylglycerol lipase (MGL) and fatty acid amide hydrolase (FAAH) for 2‐arachidonoylglycerol and anandamide, respectively. Note that these enzymes also contribute to the turnover of many endogenous ligands inactive at CB_1_ and CB_2_ cannabinoid receptors, such as N‐oleoylethanolamide, N‐palmitoylethanolamine and 2‐oleoyl glycerol. *In vitro* experiments indicate that the endocannabinoids are also substrates for oxidative metabolism *via* cyclooxygenase, lipoxygenase and cytochrome P450 enzyme activities [9, 145, 450].

**Table:** 

Nomenclature	Diacylglycerol lipase *α*	Diacylglycerol lipase *β*	*N*‐Acylphosphatidylethanolamine‐phospholipase D
Common abreviation	DGL*α*	DGL*β*	NAPE‐PLD
HGNC, UniProt	*DAGLA*, Q9Y4D2	*DAGLB*, Q8NCG7	*NAPEPLD*, Q6IQ20
EC number	3.1.1.‐	3.1.1.‐	–
Selective inhibitors	orlistat (pIC_50_ 7.2) [37], RHC80267 (pIC_50_ 4.2) [243]	orlistat (pIC_50_ 7) [37], RHC80267	–
Comments	–	–	NAPE‐PLD activity appears to be enhanced by polyamines in the physiological range [292], but fails to transphosphatidylate with alcohols [381] unlike phosphatidylcholine‐specific phospholipase D.

**Table:** 

Nomenclature	Monoacylglycerol lipase	Fatty acid amide hydrolase	Fatty acid amide hydrolase‐2	*N*‐Acylethanolamine acid amidase
Common abreviation	MGL	FAAH	FAAH2	NAAA
HGNC, UniProt	*MGLL*, Q99685	*FAAH*, O00519	*FAAH2*, Q6GMR7	*NAAA*, Q02083
EC number	3.1.1.23	3.5.1.‐	3.5.1.‐	3.5.1.‐
Rank order of affinity	2‐oleoyl glycerol = 2‐arachidonoylglycerol≫anandamide [171]	anandamide>oleamide>N‐oleoylethanolamide>N‐palmitoylethanolamine [518]	oleamide>N‐oleoylethanolamide>anandamide>N‐palmitoylethanolamine [518]	N‐palmitoylethanolamine>MEA>SEA≥N‐oleoylethanolamide>anandamide [495]
Selective inhibitors	JZL184 (pIC_50_ 8.1) [295]	JNJ1661010 (pIC_50_ 7.8) [253], PF750 (pIC_50_ 6.3–7.8) [4], OL135 (pIC_50_ 7.4) [518], URB597 (pIC_50_ 6.3–7) [518], PF3845 (pIC_50_ 6.6) [5]	URB597 (pIC_50_ 7.5–8.3) [518], OL135 (pIC_50_ 7.9) [518]	*S*‐OOPP (pIC_50_ 6.4) [451] – Rat, CCP (pIC_50_ 5.3) [492]

### Comments

Many of the compounds described as inhibitors are irreversible and so potency estimates will vary with incubation time. FAAH2 is not found in rodents [518] and few of the inhibitors described have been assessed at this enzyme activity. 2‐arachidonoylglycerol has been reported to be hydrolysed by multiple enzyme activities from neural preparations, including *ABHD6*(Q9BV23) [41], *ABHD12* (8N2K0) [41], neuropathy target esterase (*PNPLA6*, Q8IY17[316]) and carboxylesterase 1 (*CES1*, P23141[533]). Although these have been incompletely defined, WWL70 has been described to inhibit ABHD6 selectively with a pIC_50_ value of 7.2 [284]. Other selective inhibitors of NAAA (with respect to FAAH) have been described, but these are not yet commercially available.

### Further Reading

Blankman JL *et al*. (2013) Chemical probes of endocannabinoid metabolism. *Pharmacol. Rev.* **65**: 849‐71 [PMID:23512546]

Fowler CJ. (2013) Transport of endocannabinoids across the plasma membrane and within the cell. *FEBS J.* **280**: 1895‐904 [PMID:23441874]

Hermanson DJ *et al*. (2014) Substrate‐selective COX‐2 inhibition as a novel strategy for therapeutic endocannabinoid augmentation. *Trends Pharmacol. Sci.* **35**: 358‐67 [PMID:24845457]

Savinainen JR *et al*. (2012) The serine hydrolases MAGL, ABHD6 and ABHD12 as guardians of 2‐arachidonoylglycerol signalling through cannabinoid receptors. *Acta Physiol (Oxf)* **204**: 267‐76 [PMID:21418147]

Ueda N *et al*. (2013) Metabolism of endocannabinoids and related N‐acylethanolamines: canonical and alternative pathways. *FEBS J.* **280**: 1874‐94 [PMID:23425575]

Wellner N *et al*. (2013) N‐acylation of phosphatidylethanolamine and its biological functions in mammals. *Biochim. Biophys. Acta* **1831**: 652‐62 [PMID:23000428]

## Eicosanoid turnover

### Overview

Eicosanoids are 20‐carbon fatty acids, where the usual focus is the polyunsaturated analogue arachidonic acid and its metabolites. Arachidonic acid is thought primarily to derive from phospholipase A2 action on membrane phosphatidylcholine, and may be re‐cycled to form phospholipid through conjugation with coenzyme A and subsequently glycerol derivatives. Oxidative metabolism of arachidonic acid is conducted through three major enzymatic routes: cyclooxygenases; lipoxygenases and cytochrome P450‐like epoxygenases, particularly CYP2J2. Isoprostanes are structural analogues of the prostanoids (hence the nomenclature D‐, E‐, F‐isoprostanes and isothromboxanes), which are produced in the presence of elevated free radicals in a non‐enzymatic manner, leading to suggestions for their use as biomarkers of oxidative stress. Molecular targets for their action have yet to be defined.

## Cyclooxygenase

### Overview

Prostaglandin (PG) G/H synthase, most commonly referred to as cyclooxygenase (COX, (5Z,8Z,11Z,14Z)‐icosa‐5,8,11,14‐tetraenoate,hydrogen‐donor : oxygen oxidoreductase) activity, catalyses the formation of PGG_2_ from arachidonic acid. Hydroperoxidase activity inherent in the enzyme catalyses the formation of PGH_2_ from PGG_2_. COX‐1 and ‐2 can be nonselectively inhibited by ibuprofen, ketoprofen, naproxen, indomethacin and paracetamol (acetaminophen). PGH_2_ may then be metabolised to prostaglandins and thromboxanes by various prostaglandin synthases in an apparently tissue‐dependent manner.

**Table:** 

Nomenclature	COX‐1	COX‐2
HGNC, UniProt	*PTGS1*, P23219	*PTGS2*, P35354
EC number	1.14.99.1: Hydrogen donor + arachidonic acid + 2O_2_ = hydrogen acceptor + H_2_O + PGH_2_ arachidonic acid =>PGG_2_ =>PGH_2_ This enzyme is also associated with the following reaction:: docosahexaenoic acid => PGH_3_	1.14.99.1: Hydrogen donor + arachidonic acid + 2O_2_ = hydrogen acceptor + H_2_O + PGH_2_ arachidonic acid =>PGG_2_ =>PGH_2_ This enzyme is also associated with the following reaction:: docosahexaenoic acid => PGH_3_
Inhibitors	bromfenac (pIC_50_ 8.1) [17], diclofenac (pIC_50_ 7.9) [556], meclofenamic acid (pIC_50_ 7.3) [247], flurbiprofen (pIC_50_ 7.1) [512], fenoprofen (pIC_50_ 6.8) [17], ketoprofen (pIC_50_ 6.5) [55], suprofen (pIC_50_ 6.2) [55],	benzquinamide (pIC_50_ 8.3) [17], flurbiprofen (pIC_50_ 8) [25], meclofenamic acid (pIC_50_ 7.4) [247], carprofen (pIC_50_ 7) [209], ketorolac (pIC_50_ 6.9) [503], nimesulide (pIC_50_ 6.2) [366], ketoprofen (pIC_50_ 6.2) [55],
Selective inhibitors	ketorolac (pIC_50_ 9.7) [512], FR122047 (pIC_50_ 7.5) [357]	celecoxib (pIC_50_ 8.7) [38], valdecoxib (pIC_50_ 8.3) [473], diclofenac (pIC_50_ 7.7) [42], rofecoxib (pIC_50_ 6.1–6.5) [512], lumiracoxib (p*K*_i_ 6.5) [43], meloxicam (pIC_50_ 6.3) [280], etoricoxib (pIC_50_ 6) [406]

## Prostaglandin synthases

### Overview

Subsequent to the formation of PGH_2_, the cytochrome P450 activities thromboxane synthase (CYP5A1, *TBXAS1*, P24557, EC 5.3.99.5) and prostacyclin synthase (CYP8A1, *PTGIS*, Q16647, EC 5.3.99.4) generate thromboxane A_2_ and prostacyclin (PGI_2_), respectively. Additionally, multiple enzyme activities are able to generate prostaglandin E_2_(PGE_2_), prostaglandin D_2_(PGD_2_) and prostaglandin F_2*α*_(PGF_2*α*_). PGD_2_ can be metabolised to 9*α*,11*β*‐prostacyclin F_2*α*_ through the multifunctional enzyme activity of AKR1C3. PGE_2_ can be metabolised to 9*α*,11*β*‐prostaglandin F_2*α*_ through the 9‐ketoreductase activity of CBR1. Conversion of the 15‐hydroxyecosanoids, including prostaglandins, lipoxins and leukotrienes to their keto derivatives by the NAD‐dependent enzyme HPGD leads to a reduction in their biological activity.

**Table:** 

Nomenclature	CYP5A1	CYP8A1	mPGES1	mPGES2	cPGES
Common name	–	Prostacyclin synthase	–	–	–
HGNC, UniProt	*TBXAS1*, P24557	*PTGIS*, Q16647	*PTGES*, O14684	*PTGES2*, Q9H7Z7	*PTGES3*, Q15185
EC number	5.3.99.5: PGH_2_ = thromboxane A_2_	5.3.99.4	5.3.99.3: PGH_2_ = PGE_2_	5.3.99.3: PGH_2_ = PGE_2_	5.3.99.3: PGH_2_ = PGE_2_
Cofactors	–	–	glutathione	dihydrolipoic acid	–
Comments	Inhibited by dazoxiben [398] and camonagrel [182].	Converts PGH_2_ to PGI_2_ [196]. Inhibited by tranylcypromine [181]	–	–	Phosphorylated and activated by casein kinase 2 (CK2) [260]. Appears to regulate steroid hormone function by interaction with dimeric hsp90 [69, 241].

**Table:** 

Nomenclature	L‐PGDS	H‐PGDS	AKR1C3	CBR1	HPGD
HGNC, UniProt	*PTGDS*, P41222	*HPGDS*, O60760	*AKR1C3*, P42330	*CBR1*, P16152	*HPGD*, P15428
EC number	5.3.99.2: PGH_2_ = PGD_2_	5.3.99.2: PGH_2_ = PGD_2_	1.3.1.20 1.1.1.188: PGD_2_ + NADP^+^ = PGF_2*α*_ + NADPH + H^+^ 1.1.1.64 1.1.1.239 1.1.1.213	1.1.1.184 1.1.1.189: PGE_2_ + NADP^+^ = PGF_2*α*_ + NADPH + H^+^ 1.1.1.197	1.1.1.141 15‐hydroxyprostaglandins => 15‐ketoprostaglandins LXA_4_ => 15‐keto‐lipoxin A_4_
Inhibitors	–	–	flufenamic acid, indomethacin, flavonoids [321, 446]	–	–
Cofactors	–	–	NADP^+^	NADP^+^	–
Inhibitors	–	HQL‐79 (pIC_50_ 5.3–5.5) [15]	–	wedelolactone (pIC_50_ 5.4) [555]	–
Comments	–	–	Also acts as a hydroxysteroid dehydrogenase activity.	–	–

### Comments

YS121 has been reported to inhibit mPGES1 and 5‐LOX with a *p*IC_50_value of 5.5 [263].

## Lipoxygenases

### Overview

The lipoxygenases (LOXs) are a structurally related family of non‐heme iron dioxygenases that function in the production, and in some cases metabolism, of fatty acid hydroperoxides. For arachidonic acid as substrate, these products are hydroperoxyeicosatetraenoic acids (HPETEs). In humans there are five lipoxygenases, the 5S‐(arachidonate : oxygen 5‐oxidoreductase), 12R‐(arachidonate 12‐lipoxygenase, 12R‐type), 12S‐(arachidonate : oxygen 12‐oxidoreductase), and two distinct 15S‐(arachidonate : oxygen 15‐oxidoreductase) LOXs that oxygenate arachidonic acid in different positions along the carbon chain and form the corresponding 5S‐, 12S‐, 12R‐, or 15S‐hydroperoxides, respectively.

**Table:** 

Nomenclature	5‐LOX	12R‐LOX	12S‐LOX	15‐LOX‐1	15‐LOX‐2	E‐LOX
HGNC, UniProt	*ALOX5*, P09917	*ALOX12B*, O75342	*ALOX12*, P18054	*ALOX15*, P16050	*ALOX15B*, O15296	*ALOXE3*, Q9BYJ1
EC number	1.13.11.34: arachidonic acid + O_2_ = LTA_4_ + H_2_O	1.13.11.31 arachidonic acid + O_2_ =>12R‐HPETE	1.13.11.31 arachidonic acid + O_2_ =>12S‐HPETE	1.13.11.33: arachidonic acid + O_2_ = 15S‐HPETE linoleic acid + O_2_ => 13S‐HPODE	1.13.11.33: arachidonic acid + O_2_ = 15S‐HPETE	1.13.11.‐
Endogenous inhibitor	Protein kinase A‐mediated phosphorylation [304]	–	–	–	–	–
Substrates	–	methyl arachidonate	–	–	–	–
Endogenous substrates	arachidonic acid	–	–	–	–	12R‐HPETE
Endogenous activators	5‐LOX activating protein (*ALOX5AP*, P20292)	–	–	–	–	–
Selective inhibitors	CJ13610 (pIC_50_ 7.2) [136], zileuton	–	–	–	–	–
Comments	FLAP activity can be inhibited by MK‐886 [116] and BAY‐X1005 [197] leading to a selective inhibition of 5‐LOX activity	–	–	–	–	E‐LOX metabolises the product from the 12R‐lipoxygenase (12R‐HPETE) to a specific epoxyalcohol compound [543].

### Comments

An 8‐LOX (EC 1.13.11.40, arachidonate:oxygen 8‐oxidoreductase) may be the mouse orthologue of 15‐LOX‐2 [158]. Some general LOX inhibitors are nordihydroguiaretic acid and esculetin. Zileuton and caffeic acid are used as 5‐lipoxygenase inhibitors, while baicalein and CDC are 12‐lipoxygenase inhibitors. The specificity of these inhibitors has not been rigorously assessed with all LOX forms: baicalein, along with other flavonoids, such as fisetin and luteolin, also inhibits 15‐LOX‐1 [414].

## 1 Leukotriene and lipoxin metabolism

### Overview

Leukotriene A_4_(LTA_4_), produced by 5‐LOX activity, and lipoxins may be subject to further oxidative metabolism; *ω*‐hydroxylation is mediated by CYP4F2 and CYP4F3, while *β*‐oxidation in mitochondria and peroxisomes proceeds in a manner dependent on coenzyme A conjugation. Conjugation of LTA_4_ at the 6 position with reduced glutathione to generate LTC_4_ occurs under the influence of leukotriene C_4_ synthase, with the subsequent formation of LTD_4_ and LTE_4_, all three of which are agonists at CysLT receptors. LTD_4_ formation is catalysed by *γ*‐glutamyltransferase, and subsequently dipeptidase 2 removes the terminal glycine from LTD_4_ to generate LTE_4_. Leukotriene A_4_ hydrolase converts the 5,6‐epoxide LTA_4_ to the 5‐hydroxylated LTB_4_, an agonist for BLT receptors. LTA_4_ is also acted upon by 12S‐LOX to produce the trihydroxyeicosatetraenoic acids lipoxins LXA_4_ and LXB_4_. Treatment with a LTA_4_ hydrolase inhibitor in a murine model of allergic airway inflammation increased LXA_4_ levels, in addition to reducing LTB_4_, in lung lavage fluid [400].

LTA_4_ hydrolase is also involved in biosynthesis of resolvin Es. Aspirin has been reported to increase endogenous formation of 18S‐hydroxyeicosapentaenoate (18S‐HEPE) compared with 18R‐HEPE, a resolvin precursor. Both enantiomers may be metabolised by human recombinant 5‐LOX; recombinant LTA_4_hydrolase converted chiral 5S(6)‐epoxide‐containing intermediates to resolvin E1 and 18S‐resolvin E1 [358].

**Table:** 

Nomenclature	Leukotriene C4 synthase	*γ*‐Glutamyltransferase	Dipeptidase 1	Dipeptidase 2
HGNC, UniProt	*LTC4S*, Q16873	*GGCT*, O75223	*DPEP1*, P16444	*DPEP2*, Q9H4A9
EC number	4.4.1.20: LTC_4_ = glutathione + LTA_4_	2.3.2.2: (5‐L‐glutamyl)‐peptide + an amino acid = a peptide + a 5‐L‐glutamyl amino acid LTC_4_ + H_2_O =>LTD_4_ + L‐glutamate	3.4.13.19: LTD_4_ + H_2_O = LTE_4_ + glycine	3.4.13.19: LTD_4_ + H_2_O = LTE_4_ + glycine
Inhibitors	–	–	cilastatin (p*K*_i_ 6) [179] – Unknown	–

### Comments

LTA4H is a member of a family of arginyl aminopeptidases (ENSFM00250000001675), which also includes aminopeptidase B (*RNPEP*, 9H4A4) and aminopeptidase B‐like 1 (*RNPEPL1*, Q9HAU8). Dipeptidase 1 and 2 are members of a family of membrane dipeptidases, which also includes (*DPEP3*, Q9H4B8) for which LTD_4_ appears not to be a substrate.

### Further Reading

Durand T *et al*. (2011) Isoprostanes and phytoprostanes: Bioactive lipids. *Biochimie* **93**: 52‐60 [PMID:20594988]

Floyd CN *et al*. (2014) Mechanisms of aspirin resistance. *Pharmacol. Ther.* **141**: 69‐78 [PMID:23993980]

Haeggström JZ *et al*. (2011) Lipoxygenase and leukotriene pathways: biochemistry, biology, and roles in disease. *Chem. Rev.* **111**: 5866‐98 [PMID:21936577]

Korotkova M *et al*. (2014) Persisting eicosanoid pathways in rheumatic diseases. *Nat Rev Rheumatol* **10**: 229‐41 [PMID:24514915]

Krieg P *et al*. (2014) The role of lipoxygenases in epidermis. *Biochim. Biophys. Acta* **1841**: 390‐400 [PMID:23954555]

Rodríguez M *et al*. (2014) Polarization of the innate immune response by prostaglandin E2: a puzzle of receptors and signals. *Mol. Pharmacol.* **85**: 187‐97 [PMID:24170779]

Smith WL *et al*. (2011) Enzymes of the cyclooxygenase pathways of prostanoid biosynthesis. *Chem. Rev.* **111**: 5821‐65 [PMID:21942677]

Tai HH *et al*. (2011) Regulation of 15‐hydroxyprostaglandin dehydrogenase (15‐PGDH) by non‐steroidal anti‐inflammatory drugs (NSAIDs). *Prostaglandins Other Lipid Mediat.* **96**: 37‐40 [PMID:21763448]

## 2 GABA turnover

### Overview

The inhibitory neurotransmitter *γ*‐aminobutyrate (GABA, 4‐aminobutyrate) is generated in neurones by glutamic acid decarboxylase. GAD1 and GAD2 are differentially expressed during development, where GAD2 is thought to subserve a trophic role in early life and is distributed throughout the cytoplasm. GAD1 is expressed in later life and is more associated with nerve terminals [128] where GABA is principally accumulated in vesicles through the action of the vesicular inhibitory amino acid transporter SLC32A1. The role of *γ*‐aminobutyraldehyde dehydrogenase (ALDH9A1) in neurotransmitter GABA synthesis is less clear. Following release from neurons, GABA may interact with either GABA_A_ or GABA_B_ receptors and may be accumulated in neurones and glia through the action of members of the SLC6 family of transporters. Successive metabolism through GABA transaminase and succinate semialdehyde dehydrogenase generates succinic acid, which may be further metabolized in the mitochondria in the tricarboxylic acid cycle.

**Table:** 

Nomenclature	Glutamic acid	aldehyde dehydrogenase 9	4‐aminobutyrate	aldehyde dehydrogenase 5
	decarboxylase 1,	family, member A1	aminotransferase	family, member A1
	decarboxylase 2			
Common abreviation	GAD1, GAD2	–	GABA‐T	SSADH
HGNC, UniProt	*GAD1*, Q99259,	*ALDH9A1*, P49189	*ABAT*, P80404	*ALDH5A1*, P51649
	*GAD2*, Q05329			
EC number	4.1.1.15: L‐glutamic acid + H^+^ ‐>GABA + CO_2_	1.2.1.19: 4‐aminobutanal + NAD + H_2_O = GABA + NADH + H^+^ 1.2.1.47: 4‐trimethylammoniobutanal + NAD + H_2_O = 4‐trimethylammoniobutanoate + NADPH + 2H^+^ 1.2.1.3: an aldehyde + H_2_O + NAD = a carboxylate + 2H^+^ + NADH	2.6.1.19: GABA + *α*‐ketoglutaric acid = L‐glutamic acid + 4‐oxobutanoate 2.6.1.22: (S)‐3‐amino‐2‐methylpropanoate + *α*‐ketoglutaric acid = 2‐methyl‐3‐oxopropanoate + L‐glutamic acid	1.2.1.24: 4‐oxobutanoate + NAD + H_2_O = succinic acid + NADH + 2H^+^ 4‐hydroxy‐trans‐2‐nonenal + NAD + H_2_O = 4‐hydroxy‐trans‐2‐nonenoate + NADH + 2H^+^
Endogenous substrates	L‐glutamic acid, L‐aspartic acid	–	–	–
Products	GABA	–	–	–
Cofactors	pyridoxal phosphate	NAD	pyridoxal phosphate	NAD [432]
Inhibitors	–	–	vigabatrin (Irreversible inhibition) (p*K*_i_ 3.1) [289, 437]	–
Selective inhibitors	s‐allylglycine	–	–	–
Comments	L‐aspartic acid is a less rapidly metabolised substrate of mouse brain glutamic acid decarboxylase generating *β*‐alanine [530]. Autoantibodies against GAD1 and GAD2 are elevated in type 1 diabetes mellitus and neurological disorders (see Further reading).	–	–	–

### Further Reading

Errichiello L *et al*. (2011) Temporal lobe epilepsy and anti glutamic acid decarboxylase autoimmunity. *Neurol. Sci.* **32**: 547‐50 [PMID:21468678]

Kim KJ *et al*. (2011) Succinic semialdehyde dehydrogenase: biochemical‐molecular‐clinical disease mechanisms, redox regulation, and functional significance. *Antioxid. Redox Signal.* **15**: 691‐718 [PMID:20973619]

McQuail JA *et al*. (2015) Molecular aspects of age‐related cognitive decline: the role of GABA signaling. *Trends Mol Med* **21**: 450‐60 [PMID:26070271]

Pan ZZ. (2012) Transcriptional control of Gad2. *Transcription* **3**: 68‐72 [PMID:22414751]

Verrotti A *et al*. (2012) Seizures and type 1 diabetes mellitus: current state of knowledge. *Eur. J. Endocrinol.* **167**: 749‐58 [PMID:22956556]

## 3 Glycerophospholipid turnover

### Overview

Phospholipids are the basic barrier components of membranes in eukaryotic cells divided into glycerophospholipids (phosphatidic acid, phosphatidylethanolamine, phosphatidylcholine, phosphatidylserine, phosphatidylinositol and its phosphorylated derivatives) and sphingolipids (ceramide phosphorylcholine and ceramide phosphorylethanolamine).

## 4 Phosphatidylinositol kinases

### Overview

Phosphatidylinositol may be phosphorylated at either 3‐ or 4‐ positions on the inositol ring by PI 3‐kinases or PI 4‐kinases, respectively.

**Phosphatidylinositol 3‐kinases**Phosphatidylinositol 3‐kinases (PI3K, provisional nomenclature) catalyse the introduction of a phosphate into the 3‐position of phosphatidylinositol (PI), phosphatidylinositol 4‐phosphate (PIP) or phosphatidylinositol 4,5‐bisphosphate (PIP_2_). There is evidence that PI3K can also phosphorylate serine/threonine residues on proteins. In addition to the classes described below, further serine/threonine protein kinases, including ATM (Q13315) and mTOR(P42345), have been described to phosphorylate phosphatidylinositol and have been termed PI3K‐related kinases. Structurally, PI3K have common motifs of at least one C2, calcium‐binding domain and helical domains, alongside structurally‐conserved catalytic domains. Wortmannin and LY294002 are widely‐used inhibitors of PI3K activities. Wortmannin is irreversible and shows modest selectivity between Class I and Class II PI3K, while LY294002 is reversible and selective for Class I compared to Class II PI3K.

**Class I PI3Ks** (EC 2.7.1.153) phosphorylate phosphatidylinositol 4,5‐bisphosphate to generate phosphatidylinositol 3,4,5‐trisphosphate and are heterodimeric, matching catalytic and regulatory subunits. Class IA PI3Ks include p110*α*, p110*β* and p110*δ* catalytic subunits, with predominantly p85 and p55 regulatory subunits. The single catalytic subunit that forms Class IB PI3K is p110*γ*. Class IA PI3Ks are more associated with receptor tyrosine kinase pathways, while the Class IB PI3K is linked more with GPCR signalling.

**Class II PI3Ks** (EC 2.7.1.154) phosphorylate phosphatidylinositol to generate phosphatidylinositol 3‐phosphate (and possibly phosphatidylinositol 4‐phosphate to generate phosphatidylinositol 3,4‐bisphosphate). Three monomeric members exist, PI3K‐C2*α*, *β* and *γ*, and include Ras‐binding, Phox homology and two C2domains.

The only **class III PI3K** isoform (EC 2.7.1.137) is a heterodimer formed of a catalytic subunit (VPS34) and regulatory subunit (VPS15).

**Phosphatidylinositol 4‐kinases**Phosphatidylinositol 4‐kinases (EC 2.7.1.67) generate phosphatidylinositol 4‐phosphate and may be divided into higher molecular weight type III and lower molecular weight type II forms.

## 5 1‐phosphatidylinositol 4‐kinase family

**Table:** 

Nomenclature	phosphatidylinositol 4‐kinase, catalytic, alpha	phosphatidylinositol 4‐kinase, catalytic, beta
Common abreviation	PI4KIII*α*/PIK4CA	PI4KIII*β*/PIK4CB
HGNC, UniProt	*PI4KA*, P42356	*PI4KB*, Q9UBF8
EC number	2.7.1.67	2.7.1.67
Endogenous activation	–	PKD‐mediated phosphorylation [199]
(Sub)family‐selective inhibitors	wortmannin (pIC_50_ 6.7–6.8) [170, 329]	wortmannin (pIC_50_ 6.7–6.8) [170, 329]
Selective inhibitors	–	PIK‐93 [259]

## 6 Phosphatidylinositol‐4‐phosphate 3‐kinase family

**Table:** 

Nomenclature	phosphatidylinositol‐4‐phosphate 3‐kinase,	phosphatidylinositol‐4‐phosphate 3‐kinase,	phosphatidylinositol‐4‐phosphate 3‐kinase,
	catalytic subunit type 2 alpha	catalytic subunit type 2 beta	catalytic subunit type 2 gamma
Common abreviation	C2*α*/PIK3C2A	C2*β*/PIK3C2B	C2*γ*/PIK3C2G
HGNC, UniProt	*PIK3C2A*, O00443	*PIK3C2B*, O00750	*PIK3C2G*, O75747
EC number	2.7.1.154	2.7.1.154	2.7.1.154

## 7 Phosphatidylinositol 3‐kinase family

**Table:** 

Nomenclature	phosphatidylinositol 3‐kinase, catalytic subunit type 3
Common abreviation	VPS34/PIK3C3
HGNC, UniProt	*PIK3C3*, Q8NEB9
EC number	2.7.1.137

## 8 Phosphatidylinositol‐4,5‐bisphosphate 3‐kinase family

**Table:** 

Nomenclature	phosphatidylinositol‐4,	phosphatidylinositol‐4,	phosphatidylinositol‐4,	phosphatidylinositol‐4,
	5-bisphosphate 3-kinase,	5-bisphosphate 3-kinase,	5-bisphosphate 3-kinase,	5-bisphosphate 3-kinase,
	catalytic subunit alpha	catalytic subunit beta	catalytic subunit gamma	catalytic subunit delta
Common abreviation	p110*α*/PIK3CA	p110*β*/PIK3CB	p110*γ*/PIK3CG	p110*δ*/PIK3CD
HGNC, UniProt	*PIK3CA*, P42336	*PIK3CB*, P42338	*PIK3CG*, P48736	*PIK3CD*, O00329
EC number	2.7.1.153	2.7.1.153	2.7.1.153	2.7.1.153
	2.7.11.1			
Inhibitors	PIK‐75 (pIC_50_ 9.5) [200], gedatolisib (pIC_50_ 9.4) [500], PF‐04691502 (p*K*_i_ 9.2) [290], PI‐103 (pIC_50_ 8.7) [404], BGT‐226 (pIC_50_ 8.4) [315], KU‐0060648 (pIC_50_ 8.4) [60], dactolisib (pIC_50_ 8.4) [310], apitolisib (pIC_50_ 8.3) [467], alpelisib (pIC_50_ 8.3) [155], PIK‐75 (pIC_50_ 8.2) [259], buparlisib (pIC_50_ 7.5) [49], PP121 (pIC_50_ 7.3) [14]	KU‐0060648 (pIC_50_ 9.3) [60], PI‐103 (pIC_50_ 8.5) [404], AZD6482 (pIC_50_ 8) [355], ZSTK474 (pIC_50_ 7.8) [535], apitolisib (pIC_50_ 7.6) [467], BGT‐226 (pIC_50_ 7.2) [315]	dactolisib (pIC_50_ 8.3) [310], apitolisib (pIC_50_ 7.8) [467], PI‐103 (pIC_50_ 7.8) [404], BGT‐226 (pIC_50_ 7.4) [315], ZSTK474 (pIC_50_ 7.3) [535], TG‐100‐115 (pIC_50_ 7.1) [369], alpelisib (pIC_50_ 6.6) [155], KU‐0060648 (pIC_50_ 6.2) [60]	KU‐0060648 (pIC_50_>10) [60], idelalisib (*in vitro* activity against recombinant enzyme) (pIC_50_ 8.6) [276], PI‐103 (pIC_50_ 8.5) [404], ZSTK474 (pIC_50_ 8.2) [535], apitolisib (pIC_50_ 8.2) [467], dactolisib (pIC_50_ 8.1) [310], alpelisib (pIC_50_ 6.5) [155]
(Sub)family‐selective inhibitors	pictilisib (pIC_50_ 8.5) [141]	pictilisib (pIC_50_ 7.5) [141]	pictilisib (pIC_50_ 7.1) [141]	pictilisib (pIC_50_ 8.5) [141]
Selective inhibitors	–	–	CZC 24832 (p*K*_d_ 7.7) [30], CZC 24832 (pIC_50_ 7.6) [30]	–

## 9 1‐phosphatidylinositol‐3‐phosphate 5‐kinase family

**Table:** 

Nomenclature	phosphoinositide kinase, FYVE finger containing
HGNC, UniProt	*PIKFYVE*, Q9Y2I7‐4
EC number	2.7.1.150: ATP + 1‐phosphatidyl‐1D‐myo‐inositol 3‐phosphate = ADP + 1‐phosphatidyl‐1D‐myo‐inositol 3,5‐bisphosphate

## 10 Type I PIP kinases (1‐phosphatidylinositol‐4‐phosphate 5‐kinase family)

### Overview

Type I PIP kinases are required for the production of the second messenger phosphatidylinositol 4,5‐bisphosphate (PtdIns(4,5)P2) by phosphorylating PtdIns(4)P [397]. This enzyme family is also known as type I PIP(5)Ks.

**Table:** 

Nomenclature	phosphatidylinositol‐4‐phosphate 5‐kinase, type I, alpha	phosphatidylinositol‐4‐phosphate 5‐kinase, type I, gamma
Common abreviation	PIP5K1A	PIP5K1C
HGNC, UniProt	*PIP5K1A*, Q99755	*PIP5K1C*, O60331
EC number	2.7.1.68	2.7.1.68
Inhibitors	ISA‐2011B [428]	–

## 11 Type II PIP kinases (1‐phosphatidylinositol‐5‐phosphate 4‐kinase family)

### Overview

Type II PIP kinases are essential for the production of the second messenger phosphatidylinositol 4,5‐bisphosphate (PtdIns(4,5)P2) by phosphorylating PtdIns(5)P [397]. This enzyme family is also known as type II PIP(5)Ks.

**Table:** 

Nomenclature	phosphatidylinositol‐5‐phosphate 4‐kinase,	phosphatidylinositol‐5‐phosphate 4‐kinase,	phosphatidylinositol‐5‐phosphate 4‐kinase,
	type II, alpha	type II, beta	type II, gamma
Common abreviation	–	PIP4K2B	PIP4K2C
HGNC, UniProt	*PIP4K2A*, P48426	*PIP4K2B*, P78356	*PIP4K2C*, Q8TBX8
EC number	2.7.1.149	2.7.1.149	2.7.1.149
	ATP + 1‐phosphatidyl‐1D‐*m* *y* *o*‐inositol 5‐phosphate <=> ADP + 1‐phosphatidyl‐1D‐*m* *y* *o*‐inositol 4,5‐bisphosphate		

### Further Reading

Neubauer HA *et al*. (2013) Roles, regulation and inhibitors of sphingosine kinase 2. *FEBS J.* **280**: 5317‐36 [PMID:23638983]

Truman JP *et al*. (2014) Evolving concepts in cancer therapy through targeting sphingolipid metabolism. *Biochim. Biophys. Acta* **1841**: 1174‐88 [PMID:24384461]

## 12 Phosphoinositide‐specific phospholipase C

### Overview

Phosphoinositide‐specific phospholipase C (PLC, EC 3.1.4.11), catalyses the hydrolysis of PIP_2_ to IP_3_ and 1,2‐diacylglycerol, each of which have major second messenger functions. Two domains, X and Y, essential for catalytic activity, are conserved in the different forms of PLC. Isoforms of PLC‐*β* are activated primarily by G protein‐coupled receptors through members of the G_q/11_ family of G proteins. The receptor‐mediated activation of PLC‐*γ* involves their phosphorylation by receptor tyrosine kinases (RTK) in response to activation of a variety of growth factor receptors and immune system receptors. PLC‐*ε*1 may represent a point of convergence of signalling via both G protein‐coupled and catalytic receptors. Ca^2+^ ions are required for catalytic activity of PLC isoforms and have been suggested to be the major physiological form of regulation of PLC‐*δ* activity. PLC has been suggested to be activated non‐selectively by the small molecule *m*3M3FBS[21], although this mechanism of action has been questioned [271]. The aminosteroid U73122 has been described as an inhibitor of phosphoinositide‐specific PLC [447], although its selectivity among the isoforms is untested and it has been reported to occupy the H1 histamine receptor [222].

**Table:** 

Nomenclature	PLC*β*1	PLC*β*2	PLC*β*3	PLC*β*4	PLC*γ*1	PLC*γ*2
HGNC, UniProt	*PLCB1*, Q9NQ66	*PLCB2*, Q00722	*PLCB3*, Q01970	*PLCB4*, Q15147	*PLCG1*, P19174	*PLCG2*, P16885
Endogenous activators	G*α*q, G*α*11, G*β* *γ* [207, 374, 449]	G*α*16, G*β* *γ*, Rac2 (*RAC2*, P15153) [59, 223, 224, 281, 374]	G*α*q, G*β* *γ* [65, 281, 374]	G*α*q [237]	PIP_3_ [20]	PIP_3_, Rac1 (*RAC1*, P63000), Rac2 (*RAC2*, P15153), Rac3 (*RAC3*, P60763) [20, 384, 507]

**Table:** 

Nomenclature	PLC*δ*1	PLC*δ*3	PLC*δ*4	PLC*ε*1	PLC*ζ*1	PLC*η*1	PLC*η*2
HGNC, UniProt	*PLCD1*, P51178	*PLCD3*, Q8N3E9	*PLCD4*, Q9BRC7	*PLCE1*, Q9P212	*PLCZ1*, Q86YW0	*PLCH1*, Q4KWH8	*PLCH2*, O75038
Endogenous activators	Transglutaminase II, p122‐RhoGAP {Rat}, spermine, G*β* *γ* [187, 214, 344, 374]	–	–	Ras, rho [452, 525]	–	–	G*β* *γ* [551]
Endogenous inhibitors	Sphingomyelin [378]	–	–	–	–	–	–

### Comments

A series of PLC‐like proteins (*PLCL1*, Q15111; *PLCL2*, Q9UPR0 and *PLCH1*, Q4KWH8) form a family with PLC*δ* and PLC*ζ*1 isoforms, but appear to lack catalytic activity.

PLC‐*δ*2 has been cloned from bovine sources [328].

## 13 Phospholipase A_2_

### Overview

Phospholipase A_2_(PLA_2_, EC 3.1.1.4) cleaves the *sn*‐2 fatty acid of phospholipids, primarily phosphatidylcholine, to generate lysophosphatidylcholine and arachidonic acid. Most commonly‐used inhibitors (*e.g.* bromoenol lactone, arachidonyl trifluoromethyl ketone or methyl arachidonyl fluorophosphonate) are either non‐selective within the family of phospholipase A_2_ enzymes or have activity against other eicosanoid‐metabolising enzymes.

**Secreted or extracellular forms:** sPLA_2_‐1B, sPLA_2_‐2A, sPLA_2_‐2D, sPLA_2_‐2E, sPLA_2_‐2F, sPLA_2_‐3, sPLA_2_‐10 and sPLA_2_‐12A

**Cytosolic, calcium‐dependent forms:** cPLA_2_‐4A, cPLA_2_‐4B, cPLA_2_‐4C, cPLA_2_‐4D, cPLA_2_‐4E and cPLA_2_‐4F

**Other forms:** PLA_2_‐G5, iPLA_2_‐G6, PLA_2_‐G7 and PAFAH2 (platelet‐activating factor acetylhydrolase 2)

**Table:** 

Nomenclature	sPLA_2_‐1B	sPLA_2_‐2A	sPLA_2_‐2D	sPLA_2_‐2E	sPLA_2_‐2F
HGNC, UniProt	*PLA2G1B*, P04054	*PLA2G2A*, P14555	*PLA2G2D*, Q9UNK4	*PLA2G2E*, Q9NZK7	*PLA2G2F*, Q9BZM2

**Table:** 

Nomenclature	sPLA_2_‐3	sPLA_2_‐10	sPLA_2_‐12A	cPLA_2_‐4A	cPLA_2_‐4B
HGNC, UniProt	*PLA2G3*, Q9NZ20	*PLA2G10*, O15496	*PLA2G12A*, Q9BZM1	*PLA2G4A*, P47712	*PLA2G4B*, P0C869
Comments	–	–	–	cPLA_2_‐4A also expresses lysophospholipase (EC 3.1.1.5) activity [435].	–

**Table:** 

Nomenclature	cPLA_2_‐4C	cPLA_2_‐4D	cPLA_2_‐4E	cPLA_2_‐4F
HGNC, UniProt	*PLA2G4C*, Q9UP65	*PLA2G4D*, Q86XP0	*PLA2G4E*, Q3MJ16	*PLA2G4F*, Q68DD2

**Table:** 

Nomenclature	PLA_2_‐G5	iPLA_2_‐G6	PLA_2_‐G7	platelet‐activating factor
				acetylhydrolase 2, 40kDa
HGNC, UniProt	*PLA2G5*, P39877	*PLA2G6*, O60733	*PLA2G7*, Q13093	*PAFAH2*, Q99487
Inhibitors	–	–	darapladib (pIC_50_ 10) [39]	–
Selective inhibitors	–	–	rilapladib (Competitive) (pIC_50_ 9.6) [523]	–
Comments	–	–	–	PAFAH2 also expresses PAF hydrolase activity (EC 3.1.1.47)

### Comments

The sequence of PLA_2_‐2C suggests a lack of catalytic activity, while PLA_2_‐12B (GXIIB, GXIII sPLA_2_‐like) appears to be catalytically inactive [413]. A further fragment has been identified with sequence similarities to Group II PLA_2_ members. Otoconin 90 (OC90) shows sequence homology to PLA_2_‐G10.

A binding protein for secretory phospholipase A_2_ has been identified which shows modest selectivity for sPLA_2_‐1B over sPLA_2_‐2A, and also binds snake toxin phospholipase A_2_[12]. The binding protein appears to have clearance function for circulating secretory phospholipase A_2_, as well as signalling functions, and is a candidate antigen for idiopathic membraneous nephropathy [27].

PLA_2_‐G7 and PAFAH2 also express platelet‐activating factor acetylhydrolase activity (EC 3.1.1.47).

## 14 Phosphatidylcholine‐specific phospholipase D

### Overview

Phosphatidylcholine‐specific phospholipase D (PLD, EC 3.1.1.4) catalyses the formation of phosphatidic acid from phosphatidylcholine. In addition, the enzyme can make use of alcohols, such as butanol in a transphosphatidylation reaction [399].

**Table:** 

Nomenclature	PLD1	PLD2
HGNC, UniProt	*PLD1*, Q13393	*PLD2*, O14939
Endogenous activators	ADP‐ribosylation factor 1 (*ARF1*, P84077), PIP_2_, RhoA, PKC evoked phosphorylation, RalA [189, 303]	–
Endogenous inhibitor	G*β* *γ* [391]	G*β* *γ* [391]
Endogenous activators	–	ADP‐ribosylation factor 1 (*ARF1*, P84077), PIP_2_ [298], oleic acid [418]
Selective inhibitors	–	VU0364739 (pIC_50_ 7.7) [279]

### Comments

A lysophospholipase D activity (*ENPP2*, Q13822, also known as ectonucleotide pyrophosphatase/phosphodiesterase 2, phosphodiesterase I, nucleotide pyrophosphatase 2, autotaxin) has been described, which not only catalyses the production of lysophosphatidic acid (LPA) from lysophosphatidylcholine, but also cleaves ATP (see Goding *et al*., 2003 [175]). Additionally, an N‐acylethanolamine‐specific phospholipase D (*NAPEPLD*, Q6IQ20) has been characterized, which appears to have a role in the generation of endocannabinoids/endovanilloids, including anandamide[362]. This enzyme activity appears to be enhanced by polyamines in the physiological range [292] and fails to transphosphatidylate with alcohols [381].

Three further, less well‐characterised isoforms are PLD3 (*PLD3*, Q8IV08, other names Choline phosphatase 3, HindIII K4L homolog, Hu‐K4), PLD4 (*PLD4*, Q96BZ4, other names Choline phosphatase 4, Phosphatidylcholine‐hydrolyzing phospholipase, D4C14orf175 UNQ2488/PRO5775) and PLD5 (*PLD5*, Q8N7P1). PLD3 has been reported to be involved in myogenesis [365]. PLD4 is described not to have phospholipase D catalytic activity [539], but has been associated with inflammatory disorders [360, 468, 484]. Sequence analysis suggests that PLD5 is catalytically inactive.

## 15 Lipid phosphate phosphatases

### Overview

Lipid phosphate phosphatases, divided into phosphatidic acid phosphatases or lipins catalyse the dephosphorylation of phosphatidic acid (and other phosphorylated lipid derivatives) to generate inorganic phosphate and diacylglycerol. PTEN, a phosphatase and tensin homolog (BZS, MHAM, MMAC1, PTEN1, TEP1) is a phosphatidylinositol 3,4,5‐trisphosphate 3‐phosphatase which acts as a tumour suppressor by reducing cellular levels of PI 3,4,5‐P, thereby toning down activity of PDK1 and PKB. Loss‐of‐function mutations are frequently identified as somatic mutations in cancers.

**Table:** 

Nomenclature	Lipin1	Lipin2	Lipin3	PPA2A	PPA2B	PPA3A	phosphatase and
							tensin homolog
HGNC, UniProt	*LPIN1*, Q14693	*LPIN2*, Q92539	*LPIN3*, Q9BQK8	*PPAP2A*, O14494	*PPAP2B*, O14495	*PPAP2C*, O43688	*PTEN*, P60484
EC number	3.1.3.4	3.1.3.4	3.1.3.4	3.1.3.4	3.1.3.4	3.1.3.4	3.1.3.67
							3.1.3.48
							3.1.3.16
Substrates	–	phosphatidic acid	–	–	phosphatidic acid	–	phosphatidylinositol (3,4,5)‐trisphosphate

### Further Reading

Astudillo AM *et al*. (2012) Dynamics of arachidonic acid mobilization by inflammatory cells. *Biochim. Biophys. Acta* **1821**: 249‐56 [PMID:22155285]

Berridge MJ. (2009) Inositol trisphosphate and calcium signalling mechanisms. *Biochim. Biophys. Acta* **1793**: 933‐40 [PMID:19010359]

Brown HA *et al*. (2011) Introduction to lipid biochemistry, metabolism, and signaling. *Chem. Rev.* **111**: 5817‐20 [PMID:21951202]

Bunney TD *et al*. (2011) PLC regulation: emerging pictures for molecular mechanisms. *Trends Biochem. Sci.* **36**: 88‐96 [PMID:20870410]

Clayton EL *et al*. (2013) Mammalian phosphatidylinositol 4‐kinases as modulators of membrane trafficking and lipid signaling networks. *Prog. Lipid Res.* **52**: 294‐304 [PMID:23608234]

Dan P *et al*. (2012) Phospholipase A_2 activities in skin physiology and pathology. *Eur. J. Pharmacol.* **691**: 1‐8 [PMID:22819703]

Harden TK *et al*. (2011) Mechanism of activation and inactivation of Gq/phospholipase C‐*β* signaling nodes. *Chem. Rev.* **111**: 6120‐9 [PMID:21988240]

Hermansson M *et al*. (2011) Mechanisms of glycerophospholipid homeostasis in mammalian cells. *Prog. Lipid Res.* **50**: 240‐57 [PMID:21382416]

Hui DY. (2012) Phospholipase A(2) enzymes in metabolic and cardiovascular diseases. *Curr. Opin. Lipidol.* **23**: 235‐40 [PMID:22327613]

Kok BP *et al*. (2012) Unlike two peas in a pod: lipid phosphate phosphatases and phosphatidate phosphatases. *Chem. Rev.* **112**: 5121‐46 [PMID:22742522]

Liu Y *et al*. (2010) Phosphoinositide phosphatases in cell biology and disease. *Prog. Lipid Res.* **49**: 201‐17 [PMID:20043944]

Lyon AM *et al*. (2013) Structural insights into phospholipase C‐*β* function. *Mol. Pharmacol.* **84**: 488‐500 [PMID:23880553]

Quach ND *et al*. (2014) Secretory phospholipase A2 enzymes as pharmacological targets for treatment of disease. *Biochem. Pharmacol.* **90**: 338‐48 [PMID:24907600]

Selvy PE *et al*. (2011) Phospholipase D: enzymology, functionality, and chemical modulation. *Chem. Rev.* **111**: 6064‐119 [PMID:21936578]

Song MS *et al*. (2012) The functions and regulation of the PTEN tumour suppressor. *Nat. Rev. Mol. Cell Biol.* **13**: 283‐96 [PMID:22473468]

Stephens L *et al*. (2013) More paths to PI3K*γ*. *PLoS Biol.* **11**: e1001594 [PMID:23853549]

Sánchez‐Fernández G *et al*. (2014) G*α*q signalling: the new and the old. *Cell. Signal.* **26**: 833‐48 [PMID:24440667]

Woscholski R. (2014) Chemical intervention tools to probe phosphoinositide‐dependent signalling. *Biochem. Soc. Trans.* **42**: 1343‐8 [PMID:25233413]

Yagami T *et al*. (2014) The role of secretory phospholipase A_2 in the central nervous system and neurological diseases. *Mol. Neurobiol.* **49**: 863‐76 [PMID:24113843]

## 16 Haem oxygenase

### Overview

Haem oxygenase (heme,hydrogen‐donor:oxygen oxidoreductase (*α*‐methene‐oxidizing, hydroxylating)), E.C. 1.14.99.3, converts heme into biliverdin and carbon monoxide, utilizing NADPH as cofactor.

**Table:** 

Nomenclature	Haem oxygenase 1	Haem oxygenase 2
Common abreviation	HO1	HO2
HGNC, UniProt	*HMOX1*, P09601	*HMOX2*, P30519
EC number	1.14.99.3	1.14.99.3

### Comments

The existence of a third non‐catalytic version of haem oxygenase, HO3, has been proposed, although this has been suggested to be a pseudogene [202]. The chemical tin protoporphyrin IX acts as a haem oxygenase inhibitor in rat liver with an IC_50_ value of 11 nM [120].

### Further Reading

Abraham NG *et al*. (2008) Pharmacological and clinical aspects of heme oxygenase. *Pharmacol. Rev.* **60**: 79‐127 [PMID:18323402]

George EM *et al*. (2014) The heme oxygenases: important regulators of pregnancy and preeclampsia. *Am. J. Physiol. Regul. Integr. Comp. Physiol.* **307**: R769‐77 [PMID:24898840]

Gozzelino R *et al*. (2010) Mechanisms of cell protection by heme oxygenase‐1. *Annu. Rev. Pharmacol. Toxicol.* **50**: 323‐54 [PMID:20055707]

Poulos TL. (2014) Heme enzyme structure and function. *Chem. Rev.* **114**: 3919‐62 [PMID:24400737]

Rochette L *et al*. (2013) Carbon monoxide: mechanisms of action and potential clinical implications. *Pharmacol. Ther.* **137**: 133‐52 [PMID:23026155]

Wegiel B *et al*. (2013) The social network of carbon monoxide in medicine. *Trends Mol Med* **19**: 3‐11 [PMID:23140858]

## 17 Hydrogen sulphide synthesis

### Overview

Hydrogen sulfide is a putative gasotransmitter, with similarities to nitric oxide and carbon monoxide. Although the enzymes indicated have multiple enzymatic activities, the focus here is the generation of hydrogen sulphide and the enzymatic characteristics are described accordingly. Cystathionine *β*‐synthase and cystathionine *γ*‐lyase are pyridoxal phosphate‐dependent enzymes, while 3‐mercaptopyruvate sulfurtransferase functions as a pyridoxal phosphate‐independent pathway.

**Table:** 

Nomenclature	Cystathionine *β*‐synthase	Cystathionine *γ*‐lyase	L‐Cysteine:2‐oxoglutarate	3‐Mercaptopyruvate
			aminotransferase	sulfurtransferase
Common abreviation	CBS	CSE	CAT	MPST
HGNC, UniProt	*CBS*, P35520	*CTH*, P32929	*CCBL1*, Q16773	*MPST*, P25325
EC number	4.2.1.22	4.4.1.1	4.4.1.13	2.8.1.2
Endogenous substrates	L‐cysteine (*K*_m_6 × 10^−3^M) [74], L‐homocysteine	L‐cysteine	L‐cysteine	3‐mercaptopyruvic acid (*K*_m_1.2 × 10^−3^M) [345]
Products	cystathionine	NH_3_, pyruvic acid	NH_3_, pyruvic acid	pyruvic acid
Cofactors	pyridoxal phosphate	pyridoxal phosphate	pyridoxal phosphate	Zn^2+^
Inhibitors	aminooxyacetic acid	propargylglycine	–	–

### Further Reading

Bełtowski J. (2015) Hydrogen sulfide in pharmacology and medicine–An update. *Pharmacol Rep* **67**: 647‐58 [PMID:25933982]

Li L *et al*. (2011) Hydrogen sulfide and cell signaling. *Annu. Rev. Pharmacol. Toxicol.* **51**: 169‐87 [PMID:21210746]

Nagy P *et al*. (2014) Chemical aspects of hydrogen sulfide measurements in physiological samples. *Biochim. Biophys. Acta* **1840**: 876‐91 [PMID:23769856]

Wallace JL *et al*. (2015) Hydrogen sulfide‐based therapeutics: exploiting a unique but ubiquitous gasotransmitter. *Nat Rev Drug Discov* **14**: 329‐45 [PMID:25849904]

Wang R *et al*. (2015) The role of H2S bioavailability in endothelial dysfunction. *Trends Pharmacol. Sci.* [PMID:26071118]

## 18 Hydrolases

### Overview

listed in this section are hydrolases not accumulated in other parts of the Concise Guide, such as monoacylglycerol lipase and acetylcholinesterase. Pancreatic lipase is the predominant mechanism of fat digestion in the alimentary system; its inhibition is associated with decreased fat absorption. CES1 is present at lower levels in the gut than CES2 (P23141), but predominates in the liver, where it is responsible for the hydrolysis of many aliphatic, aromatic and steroid esters. Hormone‐sensitive lipase is also a relatively non‐selective esterase associated with steroid ester hydrolysis and triglyceride metabolism, particularly in adipose tissue. Endothelial lipase is secreted from endothelial cells and regulates circulating cholesterol in high density lipoproteins.

**Table:** 

Nomenclature	pancreatic lipase	lipase, endothelial	carboxylesterase 1	lipase, hormone‐sensitive
Common abreviation	PNLIP	LIPG	CES1	LIPE
HGNC, UniProt	*PNLIP*, P16233	*LIPG*, Q9Y5X9	*CES1*, P23141	*LIPE*, Q05469
EC number	3.1.1.3	3.1.1.3	3.1.1.1	3.1.1.79
Inhibitors	orlistat (pIC_50_ 8.9) [51]	–	–	–

### Further Reading

Markey, G.M. (2011) Carboxylesterase 1 (Ces1): from monocyte marker to major player. *J Clin Pathol* **64**: 107‐109 [PMID:21177752]

## 19 Inositol phosphate turnover

### Overview

The sugar alcohol D‐*myo*‐inositol is a component of the phosphatidylinositol signalling cycle, where the principal second messenger is inositol 1,4,5‐trisphosphate, IP_3_, which acts at intracellular ligand‐gated ion channels, IP_3_ receptors to elevate intracellular calcium. IP_3_ is recycled to inositol by phosphatases or phosphorylated to form other active inositol polyphosphates. Inositol produced from dephosphorylation of IP_3_ is recycled into membrane phospholipid under the influence of phosphatidyinositol synthase activity (CDP‐diacylglycerol‐inositol 3‐phosphatidyltransferase [EC 2.7.8.11]).

## 20 Inositol 1,4,5‐trisphosphate 3‐kinases

### Overview

Inositol 1,4,5‐trisphosphate 3‐kinases (E.C. 2.7.1.127, ENSFM00250000001260) catalyse the generation of inositol 1,3,4,5‐tetrakisphosphate (IP_4_) from IP_3_. IP_3_ kinase activity is enhanced in the presence of calcium/calmodulin(*CALM1* *CALM2* *CALM3*, P62158) [91].

## 21 Inositol polyphosphate phosphatases

### Overview

Members of this family exhibit phosphatase activity towards IP_3_, as well as towards other inositol derivatives, including the phospholipids PIP_2_ and PIP_3_. With IP_3_ as substrate, 1‐phosphatase (EC 3.1.3.57) generates 4,5,‐IP_2_, 4‐phosphatases (EC 3.1.3.66, ENSFM00250000001432) generate 1,5,‐IP_2_ and 5‐phosphatases (E.C. 3.1.3.36 or 3.1.3.56) generate 1,4,‐IP_2_.

### Comments

*In vitro* analysis suggested IP_3_ and IP_4_ were poor substrates for SKIP, synaptojanin 1 and synaptojanin 2, but suggested that PIP_2_ and PIP_3_ were more efficiently hydrolysed [422].

## 22 Inositol monophosphatase

### Overview

Inositol monophosphatase (E.C. 3.1.3.25, IMPase, *myo*‐inositol‐1(or 4)‐phosphate phosphohydrolase) is a magnesium‐dependent homodimer which hydrolyses *myo*‐inositol monophosphate to generate *myo*‐inositol and phosphate. Glycerol may be a physiological phosphate acceptor. Li^+^ is a nonselective un‐competitive inhibitor more potent at IMPase 1 (*p*K_i_ *ca*. 3.5, [324]; *p*IC_50_ 3.2, [359]) than IMPase 2 (*p*IC_50_ 1.8‐2.1, [359]). IMPase activity may be inhibited competitively by L690330(*p*K_i_ 5.5, [324]), although the enzyme selectivity is not yet established.

**Table:** 

Nomenclature	IMPase 1	IMPase 2
HGNC, UniProt	*IMPA1*, P29218	*IMPA2*, O14732
EC number	3.1.3.25	3.1.3.25
Rank order of affinity	inositol 4‐phosphate>inositol 3‐phosphate>inositol 1‐phosphate [324]	–
Inhibitors	Li^+^ (p*K*_i_ 3.5) [324]	–

### Comments

Polymorphisms in either of the genes encoding these enzymes have been linked with bipolar disorder [443, 444, 540]. Disruption of the gene encoding IMPase 1, but not IMPase 2, appears to mimic the effects of Li^+^ in mice [97, 98].

### Further Reading

Barker CJ *et al*. (2013) New horizons in cellular regulation by inositol polyphosphates: insights from the pancreatic *β*‐cell. *Pharmacol. Rev.* **65**: 641‐69 [PMID:23429059]

Billcliff PG *et al*. (2014) Inositol lipid phosphatases in membrane trafficking and human disease. *Biochem. J.* **461**: 159‐75 [PMID:24966051]

Chiu CT *et al*. (2010) Molecular actions and therapeutic potential of lithium in preclinical and clinical studies of CNS disorders. *Pharmacol. Ther.* **128**: 281‐304 [PMID:20705090]

Pirruccello M *et al*. (2012) Inositol 5‐phosphatases: insights from the Lowe syndrome protein OCRL. *Trends Biochem. Sci.* **37**: 134‐43 [PMID:22381590]

Schell MJ. (2010) Inositol trisphosphate 3‐kinases: focus on immune and neuronal signaling. *Cell. Mol. Life Sci.* **67**: 1755‐78 [PMID:20066467]

## 23 Lanosterol biosynthesis pathway

### Overview

Lanosterol is a precursor for cholesterol, which is synthesized primarily in the liver in a pathway often described as the mevalonate or HMG‐CoA reductase pathway. The first two steps (formation of acetoacetyl CoA and the mitochondrial generation of (S)‐3‐hydroxy‐3‐methylglutaryl‐CoA) are also associated with oxidation of fatty acids.

**Table:** 

Nomenclature	acetyl‐CoA acetyltransferase 1, acetyl‐CoA acetyltransferase 2	hydroxymethylglutaryl‐CoA synthase 1, hydroxymethylglutaryl‐CoA synthase 2
HGNC, UniProt	*ACAT1*, P24752, *ACAT2*, Q9BWD1	*HMGCS1*, Q01581, *HMGCS2*, P54868
EC number	2.3.1.9: 2acetyl CoA = acetoacetyl CoA + coenzyme A	2.3.3.10: acetyl CoA + H_2_O + acetoacetyl CoA ‐>(S)‐3‐hydroxy‐3‐methylglutaryl‐CoA + coenzyme A
Comments	–	HMGCoA synthase is found in cytosolic (HMGCoA synthase 1) and mitochondrial (HMGCoA synthase 2) versions; the former associated with (R)‐mevalonate synthesis and the latter with ketogenesis.

**Table:** 

Nomenclature	hydroxymethylglutaryl‐CoA	mevalonate kinase	phosphomevalonate kinase	diphosphomevalonate
	reductase			decarboxylase
HGNC, UniProt	*HMGCR*, P04035	*MVK*, Q03426	*PMVK*, Q15126	*MVD*, P53602
EC number	1.1.1.34: (S)‐3‐hydroxy‐3‐methylglutaryl‐CoA + NADPH ‐>(R)‐mevalonate + coenzyme A + NADP^+^ Reaction mechanism:: **First step:** (S)‐3‐hydroxy‐3‐methylglutaryl‐CoA + NADPH ‐> mevaldyl‐CoA + NADP^+^ **Second step:** mevaldyl‐CoA + H2O ‐> (R)‐mevalonate + NADP^+^	2.7.1.36: ATP + (R)‐mevalonate ‐>adenosine diphosphate + (R)‐5‐phosphomevalonate	2.7.4.2: ATP + (R)‐5‐phosphomevalonate = adenosine diphosphate + (R)‐5‐diphosphomevalonate	4.1.1.33: ATP + (R)‐5‐diphosphomevalonate ‐>adenosine diphosphate + isopentenyl diphosphate + CO_2_ + PO_3_^4‐^
Inhibitors	lovastatin (Competitive) (p*K*_i_ 9.2) [8], rosuvastatin (Competitive) (pIC_50_ 8.3) [228], cerivastatin (Competitive) (p*K*_i_ 8.2) [61], atorvastatin (Competitive) (pIC_50_ 8.1) [228], cerivastatin (Competitive) (pIC_50_ 8) [486], simvastatin (Competitive) (pIC_50_ 8) [228], fluvastatin (Competitive) (pIC_50_ 7.6) [228]	–	–	–
Comments	HMGCoA reductase is associated with intracellular membranes; enzymatic activity is inhibited by phosphorylation by AMP‐activated kinase. The enzymatic reaction is a three‐step reaction involving the intermediate generation of mevaldehyde‐CoA and mevaldehyde.	Mevalonate kinase activity is regulated by the downstream products farnesyl diphosphate and geranyl diphosphate as an example of feedback inhibition.	–	–

**Table:** 

Nomenclature	isopentenyl‐diphosphate	isopentenyl‐diphosphate	geranylgeranyl diphosphate synthase	farnesyl diphosphate synthase
	Δ‐isomerase 1	Δ‐isomerase 1		
HGNC, UniProt	*IDI1*, Q13907	*IDI2*, Q9BXS1	*GGPS1*, O95749	*FDPS*, P14324
EC number	5.3.3.2: isopentenyl diphosphate = dimethylallyl diphosphate	5.3.3.2: isopentenyl diphosphate = dimethylallyl diphosphate	2.5.1.29: trans,trans‐farnesyl diphosphate + isopentenyl diphosphate ‐>geranylgeranyl diphosphate + diphosphate 2.5.1.10: geranyl diphosphate + isopentenyl diphosphate ‐>trans,trans‐farnesyl diphosphate + diphosphate 2.5.1.1: dimethylallyl diphosphate + isopentenyl diphosphate = geranyl diphosphate + diphosphate	2.5.1.10: geranyl diphosphate + isopentenyl diphosphate ‐>trans,trans‐farnesyl diphosphate + diphosphate 2.5.1.1: dimethylallyl diphosphate + isopentenyl diphosphate = geranyl diphosphate + diphosphate
Inhibitors	–	–	–	risedronate (pIC_50_ 8.4) [31], zoledronic acid (p*K*_i_ 7.1) [121], alendronate (pIC_50_ 6.3) [31]
Selective inhibitors	–	–	–	ibandronic acid (p*K*_i_ 6.7) [121], pamidronic acid (pIC_50_ 6.7) [121]

**Table:** 

Nomenclature	squalene synthase	squalene monooxygenase	lanosterol synthase
HGNC, UniProt	*FDFT1*, P37268	*SQLE*, Q14534	*LSS*, P48449
EC number	2.5.1.21: 2trans,trans‐farnesyl diphosphate ‐>presqualene diphosphate + diphosphate presqualene diphosphate + NAD(P)H + H^+^ ‐>squalene + diphosphate + NAD(P)^+^	1.14.13.132: H^+^ + NADPH + O_2_ + squalene = H_2_O + NADP^+^ + (*S*)‐2,3‐epoxysqualene	5.4.99.7: (*S*)‐2,3‐epoxysqualene = lanosterol
Cofactors	NADPH	–	–
Inhibitors	zaragozic acid A (p*K*_i_ 10.1) [32] – Rat, zaragozic acid A (pIC_50_ 9.2) [488]	–	–

### Further Reading

Miziorko HM. (2011) Enzymes of the mevalonate pathway of isoprenoid biosynthesis. *Arch. Biochem. Biophys.* **505**: 131‐43 [PMID:20932952]

Rozman D *et al*. (2010) Perspectives of the non‐statin hypolipidemic agents. *Pharmacol. Ther.* **127**: 19‐40 [PMID:20420853]

Seiki S *et al*. (2009) Pharmacologic inhibition of squalene synthase and other downstream enzymes of the cholesterol synthesis pathway: a new therapeutic approach to treatment of hypercholesterolemia. *Cardiol Rev* **17**: 70‐6 [PMID:19367148]

Zhang H *et al*. (2014) Cholesterol and lipoprotein metabolism: Early Career Committee contribution. *Arterioscler. Thromb. Vasc. Biol.* **34**: 1791‐4 [PMID:25142876]

van der Burgh R *et al*. (2012) Mevalonate kinase deficiency, a metabolic autoinflammatory disease. *Clin. Immunol.* [PMID:23110805]

## 24 Nucleoside synthesis and metabolism

### Overview

The *de novo* synthesis and salvage of nucleosides have been targetted for therapeutic advantage in the treatment of particular cancers and gout. Dihydrofolate reductase produces tetrahydrofolate, a cofactor required for synthesis of purines, pyrimidines and amino acids. GART allows formylation of phosphoribosylglycinamide, an early step in purine biosynthesis. Dihydroorotate dehydrogenase produces orotate, a key intermediate in pyrimidine synthesis. IMP dehydrogenase generates xanthosine monophosphate, an intermediate in GTP synthesis.

**Table:** 

Nomenclature	dihydrofolate reductase	dihydroorotate dehydrogenase	IMP (inosine 5'‐monophosphate)	IMP (inosine 5'‐monophosphate)
		(quinone)	dehydrogenase 1	dehydrogenase 2
HGNC, UniProt	*DHFR*, P00374	*DHODH*, Q02127	*IMPDH1*, P20839	*IMPDH2*, P12268
EC number	1.5.1.3	1.3.5.2	1.1.1.205	1.1.1.205
Inhibitors	pemetrexed (p*K*_i_ 8.1) [161, 436], pralatrexate (p*K*_i_ 7.3) [231]	teriflunomide (p*K*_i_ 7.5) [204], leflunomide (p*K*_i_ 4.9) [372]	mycophenolic acid (pIC_50_ 7.7) [351], ribavirin (pIC_50_ 5.6–6) [526], mycophenolate mofetil, thioguanine [124, 502]	mycophenolic acid (pIC_50_ 7.7) [351], ribavirin (pIC_50_ 5.6–6) [526], mycophenolate mofetil (See **Inhibitor Comment** below), thioguanine [124, 502]
Selective inhibitors	methotrexate (p*K*_i_ 8.9) [412]	–	–	–

**Table:** 

Nomenclature	xanthine dehydrogenase	ribonucleotide reductase M1	ribonucleotide reductase M2	ribonucleotide reductase M2
				B (TP53 inducible)
HGNC, UniProt	*XDH*, P47989	*RRM1*, P23921	*RRM2*, P31350	*RRM2B*, Q7LG56
EC number	1.17.1.4	1.17.14.1	1.17.4.1	1.17.1.4
Inhibitors	febuxostat (p*K*_i_ 9.9) [361] – Bovine, febuxostat (pIC_50_ 7.5) [255] – Bovine, allopurinol (pIC_50_ 5.4) [34], allopurinol (p*K*_i_ 5.2) [34]	clofarabine (pIC_50_ 8.3) [375], fludarabine (pIC_50_ 6) [491], hydroxyurea (pIC_50_ 3.8) [433], gemcitabine [205]	clofarabine (pIC_50_ 8.3) [375], fludarabine (pIC_50_ 6) [491], hydroxyurea (pIC_50_ 3.8) [433], gemcitabine [205]	–

**Table:** 

Nomenclature	thymidylate synthetase	phosphoribosylglycinamide formyltransferase,	purine nucleoside phosphorylase
		phosphoribosylglycinamide synthetase,	
		phosphoribosylaminoimidazole synthetase	
Common abreviation	–	GART	–
HGNC, UniProt	*TYMS*, P04818	*GART*, P22102	*PNP*, P00491
EC number	2.1.1.45	2.1.2.2	–
		6.3.3.1	
		6.3.4.13	
Inhibitors	pemetrexed (p*K*_i_ 7) [436], capecitabine [63, 373]	pemetrexed (p*K*_i_ 5) [436] – Mouse	–
Selective inhibitors	raltitrexed (pIC_50_ 6.5) [162]	–	–

### Comments

Thymidylate synthetase allows the interconversion of dUMP and dTMP, thereby acting as a crucial step in DNA synthesis. Purine nucleoside phosphorylase allows separation of a nucleoside into the nucleobase and ribose phosphate for nucleotide salvage. Xanthine dehydrogenase generates urate in the purine degradation pathway. Post‐translational modifications of xanthine dehydrogenase convert the enzymatic reaction to a xanthine oxidase, allowing the interconversion of hypoxanthine and xanthine, with the production (or consumption) of reactive oxygen species. Ribonucleotide reductases allow the production of deoxyribonucleotides from ribonucleotides.

### Further Reading

Battelli MG *et al*. (2014) Pathophysiology of circulating xanthine oxidoreductase: new emerging roles for a multi‐tasking enzyme. *Biochim. Biophys. Acta* **1842**: 1502‐17 [PMID:24882753]

Cantu‐Medellin N *et al*. (2013) Xanthine oxidoreductase‐catalyzed reduction of nitrite to nitric oxide: insights regarding where, when and how. *Nitric Oxide* **34**: 19‐26 [PMID:23454592]

Glander P *et al*. (2012) Inosine 5'‐monophosphate dehydrogenase activity as a biomarker in the field of transplantation. *Clin. Chim. Acta* **413**: 1391‐7 [PMID:21889500]

Munier‐Lehmann H *et al*. (2013) On dihydroorotate dehydrogenases and their inhibitors and uses. *J. Med. Chem.* **56**: 3148‐67 [PMID:23452331]

## 26 Sphingosine 1‐phosphate turnover

### Overview

S1P (sphingosine 1‐phosphate) is a pro‐survival signal, in contrast to ceramide. It is formed by the sphingosine kinase‐catalysed phosphorylation of sphingosine. S1P can be released from cells to act as an agonist at a family of five G protein‐coupled receptors (S1P_1‐5_) but also has intracellular targets. S1P can be dephosphorylated back to sphingosine or hydrolysed to form hexadecanal and phosphoethanolamine. Sphingosine choline phosphotransferase (EC 2.7.8.10) generates sphingosylphosphocholine from sphingosine and CDP‐choline. Sphingosine *β*‐galactosyltransferase (EC 2.4.1.23) generates psychosine from sphingosine in the presence of UDP‐*α*‐D‐galactose. The molecular identities of these enzymes have not been confirmed.

### Further Reading

Chan H *et al*. (2013) Post‐translational regulation of sphingosine kinases. *Biochim. Biophys. Acta* **1831**: 147‐56 [PMID:22801036]

Khavandgar Z *et al*. (2015) Sphingolipid metabolism and its role in the skeletal tissues. *Cell. Mol. Life Sci.* **72**: 959‐69 [PMID:25424644]

Maceyka M *et al*. (2014) Sphingolipid metabolites in inflammatory disease. *Nature* **510**: 58‐67 [PMID:24899305]

Plano D *et al*. (2014) Importance of sphingosine kinase (SphK) as a target in developing cancer therapeutics and recent developments in the synthesis of novel SphK inhibitors. *J. Med. Chem.* **57**: 5509‐24 [PMID:24471412]

Pyne S *et al*. (2011) Translational aspects of sphingosine 1‐phosphate biology. *Trends Mol Med* **17**: 463‐72 [PMID:21514226]

Rosen H *et al*. (2013) Sphingosine‐1‐phosphate and its receptors: structure, signaling, and influence. *Annu. Rev. Biochem.* **82**: 637‐62 [PMID:23527695]

Schwalm S *et al*. (2014) Targeting the sphingosine kinase/sphingosine 1‐phosphate pathway to treat chronic inflammatory kidney diseases. *Basic Clin. Pharmacol. Toxicol.* **114**: 44‐9 [PMID:23789924]

## 27 Sphingosine kinase

**Table:** 

Nomenclature	sphingosine kinase 1 sphingosine kinase 2
Common abreviation	SPHK1, SPHK2
HGNC, UniProt	*SPHK1*, Q9NYA1 *SPHK2*, Q9NRA0
EC number	2.7.1.91: sphingosine + ATP = sphingosine 1‐phosphate + adenosine diphosphate ATP + sphinganine = sphinganine 1‐phosphate + adenosine diphosphate
Cofactors	Mg^2+^ [432]
Inhibitors	PF‐543 (pIC_50_ 8.7) [425], SK1‐I [377], ABC294640 [148], ROMe [287]
(Sub)family‐selective inhibitors	sphingosine kinase inhibitor (pIC_50_ 6.3) [147]

### Further Reading

Neubauer HA *et al*. (2013) Roles, regulation and inhibitors of sphingosine kinase 2. *FEBS J.* **280**: 5317‐36 [PMID:23638983]

Truman JP *et al*. (2014) Evolving concepts in cancer therapy through targeting sphingolipid metabolism. *Biochim. Biophys. Acta* **1841**: 1174‐88 [PMID:24384461]

## 28 Sphingosine 1‐phosphate phosphatase

**Table:** 

Nomenclature	sphingosine‐1‐phosphate phosphatase 1	sphingosine‐1‐phosphate phosphatase 2
Common abreviation	SGPP1	SGPP2
HGNC, UniProt	*SGPP1*, Q9BX95	*SGPP2*, Q8IWX5
EC number	3.1.3.‐: sphingosine 1‐phosphate ‐>sphingosine + inorganic phosphate	3.1.3.‐: sphingosine 1‐phosphate ‐>sphingosine + inorganic phosphate
Comments	Depletion of S1P phosphohydrolase‐1 (SPP1), which degrades intracellular S1P, induces the unfolded protein response and endoplasmic reticulum stress‐induced autophagy [307].	–

## 29 Sphingosine 1‐phosphate lyase

**Table:** 

Nomenclature	sphingosine‐1‐phosphate lyase 1
HGNC, UniProt	*SGPL1*, O95470
EC number	4.1.2.27: sphingosine 1‐phosphate ‐>phosphoethanolamine + hexadecanal
Cofactors	pyridoxal phosphate
Inhibitors	compound 31 [PMID: 24809814] (pIC_50_ 6.7) [519]
Comments	THI (2‐Acetyl‐5‐tetrahydroxybutyl imidazole) inhibits the enzyme activity in intact cell preparations [426].

### Further Reading

Bigaud M *et al*. (2014) Second generation S1P pathway modulators: research strategies and clinical developments. *Biochim. Biophys. Acta* **1841**: 745‐58 [PMID:24239768]

Van Veldhoven PP *et al*. (2000) Human sphingosine‐1‐phosphate lyase: cDNA cloning, functional expression studies and mapping to chromosome 10q22(1). *Biochim. Biophys. Acta* **1487**: 128‐34 [PMID:11018465]

## 30 Thyroid hormone turnover

### Overview

The thyroid hormones triiodothyronine and thyroxine, usually abbreviated as triiodothyronine and T_4_, respectively, are synthesized in the thyroid gland by sequential metabolism of tyrosine residues in the glycosylated homodimeric protein thyroglobulin (*TG*, P01266) under the influence of the haem‐containing protein iodide peroxidase. Iodide peroxidase/TPO is a haem‐containing enzyme, from the same structural family as eosinophil peroxidase (*EPX*, P11678), lactoperoxidase (LPO, P22079) and myeloperoxidase (MPO, P05164). Circulating thyroid hormone is bound to thyroxine‐binding globulin (SERPINA7, P05543).

**Tissue deiodinases**These are 1 TM selenoproteins that remove an iodine from T_4_(3,3',5,5'‐tetraiodothyronine) to generate triiodothyronine(3,3',5‐triiodothyronine, a more potent agonist at thyroid hormone receptors) or rT_3_(rT3, 3,3',5'‐triiodothyronine, a relatively inactive analogue). DIO1 is also able to deiodinate RT3 to form 3,3'‐diidothyronine (T_2_). Iodotyrosine deiodinase is a 1TM homodimeric enzyme.

**Table:** 

Nomenclature	thyroid peroxidase	deiodinase, iodothyronine,	deiodinase, iodothyronine,	deiodinase, iodothyronine,	iodotyrosine deiodinase
		type I	type II	type III	
Common abreviation	TPO	DIO1	DIO2	DIO3	IYD
HGNC, UniProt	*TPO*, P07202	*DIO1*, P49895	*DIO2*, Q92813	*DIO3*, P55073	*IYD*, Q6PHW0
EC number	1.11.1.8: [Thyroglobulin]‐L‐tyrosine + H_2_O_2_ + H^+^ + I^‐^ ‐> [Thyroglobulin]‐3,5,3'‐triiodo‐L‐thyronine + [thyroglobulin]‐aminoacrylate + H_2_O	1.97.1.10: T_4_ ‐>triiodothyronine rT_3_ ‐>T_2_	1.97.1.10: T_4_ ‐>triiodothyronine rT_3_ ‐>T_2_	1.97.1.11: T_4_ ‐>triiodothyronine rT_3_ ‐>T_2_	1.22.1.1: 3‐iodotyrosine ‐>L‐tyrosine + I^‐^ 3,5‐diiodo‐L‐tyrosine ‐>3‐iodotyrosine + I^‐^
Cofactors	Ca^2+^	–	–	–	flavin adenine dinucleotide, NADPH
Inhibitors	methimazole [349], propylthiouracil [349]	–	–	–	–
Comments	Carbimazole is a pro‐drug for methimazole	–	–	–	–

### Further Reading

Arrojo E Drigo R *et al*. (2013) Role of the type 2 iodothyronine deiodinase (D2) in the control of thyroid hormone signaling. *Biochim. Biophys. Acta* **1830**: 3956‐64 [PMID:22967761]

Darras VM *et al*. (2015) Intracellular thyroid hormone metabolism as a local regulator of nuclear thyroid hormone receptor‐mediated impact on vertebrate development. *Biochim. Biophys. Acta* **1849**: 130‐41 [PMID:24844179]

Darras VM *et al*. (2012) Iodothyronine deiodinase structure and function: from ascidians to humans. *J. Endocrinol.* **215**: 189‐206 [PMID:22825922]

Gereben B *et al*. (2008) Cellular and molecular basis of deiodinase‐regulated thyroid hormone signaling. *Endocr. Rev.* **29**: 898‐938 [PMID:18815314]

Waung JA *et al*. (2012) Thyroid hormone metabolism in skeletal development and adult bone maintenance. *Trends Endocrinol. Metab.* **23**: 155‐62 [PMID:22169753]

## 31 1.14.11.29 2‐oxoglutarate oxygenases

### Overview

The hypoxia inducible factor (HIF) is a transcriptional complex that is involved in oxygen homeostasis [429]. At normal oxygen levels, the alpha subunit of HIF (HIF‐1*α*) is targeted for degradation by prolyl hydroxylation by the PHD proteins 1‐3 (HIF‐PHs) whch are 2‐oxoglutarate (2OG) oxygenases responsible for the post‐translational modification of a specific proline in each of the oxygen‐dependent degradation (ODD) domains of HIF‐1*α*. Hydroxylated HIFs are then targeted for proteasomal degradation *via* the von Hippel‐Lindau ubiquitination complex [232]. Under hypoxic conditions, the hydroxylation reaction is blunted which results in decreased HIF degradation. The surviving HIFs are then available to translocate to the nucleus where they heterodimerize with HIF‐1*β*, effecting increased expression of hypoxia‐inducible genes.

HIF‐PH enzymes are being investigated as pharmacological targets as their inhibition mimics the hypoxic state and switches on transcription of genes associated with processes such as erythropoiesis and vasculogenesis [142]. Small molecule HIF‐PH inhibitors are in clinical trial as novel therapies for the amelioration of anemia associated with chronic kidney disease [46].

**Table:** 

Nomenclature	egl‐9 family hypoxia‐inducible factor 2	egl‐9 family hypoxia‐inducible factor 1	egl‐9 family hypoxia‐inducible factor 3
Common abreviation	PHD1	PHD2	PHD3
HGNC, UniProt	*EGLN2*, Q96KS0	*EGLN1*, Q9GZT9	*EGLN3*, Q9H6Z9
EC number	–	1.14.11.29	1.14.11.29
Inhibitors	–	IOX2 (pIC_50_ 7.7) [84]	–

## 32 2.4.2.30 poly(ADP‐ribose)polymerases

### Overview

The Poly ADP‐ribose polymerase family is a series of enzymes, where the best characterised members are nuclear proteins which are thought to function by binding to single strand breaks in DNA, allowing the recruitment of repair enzymes by the synthesis of NAD‐derived ADP‐ribose polymers, which are subsequently degraded by a glycohydrolase (*PARG*, Q86W56).

**Table:** 

Nomenclature	poly (ADP‐ribose) polymerase 1	poly (ADP‐ribose) polymerase 2	poly (ADP‐ribose) polymerase 3
Common abreviation	PARP1	PARP2	PARP3
HGNC, UniProt	*PARP1*, P09874	*PARP2*, Q9UGN5	*PARP3*, Q9Y6F1
EC number	2.4.2.30	2.4.2.30	–
Selective inhibitors	AG14361 (p*K*_i_ 8.2) [445]	–	–

### Further Reading

Bürkle A *et al*. (2013) Poly(ADP‐ribose): PARadigms and PARadoxes. *Mol. Aspects Med.* **34**: 1046‐65 [PMID:23290998]

De Vos M *et al*. (2012) The diverse roles and clinical relevance of PARPs in DNA damage repair: current state of the art. *Biochem. Pharmacol.* **84**: 137‐46 [PMID:22469522]

Lord CJ *et al*. (2012) The DNA damage response and cancer therapy. *Nature* **481**: 287‐94 [PMID:22258607]

Sonnenblick A *et al*. (2015) An update on PARP inhibitors–moving to the adjuvant setting. *Nat Rev Clin Oncol* **12**: 27‐41 [PMID:25286972]

## 33 2.5.1.58 Protein farnesyltransferase

### Overview

Farnesyltransferase is a member of the prenyltransferases family which also includes geranylgeranyltransferase types I (EC 2.5.1.59) and II (EC 2.5.1.60) [66]. Protein farnesyltransferase catalyses the post‐translational formation of a thioether linkage between the C‐1 of an isoprenyl group and a cysteine residue fourth from the C‐terminus of a protein (*ie* to the CaaX motif, where 'a' is an aliphatic amino acid and 'X' is usually serine, methionine, alanine or glutamine; leucine for EC 2.5.1.59) [156]. Farnesyltransferase is a dimer, composed of an alpha and beta subunit and requires Mg^2+^ and Zn^2+^ ions as cofactors. The active site is located between the subunits. Prenylation creates a hydrophobic domain on protein tails which acts as a membrane anchor.

Substrates of the prenyltransferases include Ras, Rho, Rab, other Ras‐related small GTP‐binding proteins, G‐protein *γ*‐subunits, nuclear lamins, centromeric proteins and many proteins involved in visual signal transduction.

In relation to the causative association between oncogenic Ras proteins and cancer, farnesyltransferase has become an important mechanistic drug discovery target.

## 35 3.5.3.15 Peptidyl arginine deiminases (PADI)

### Overview

In humans, the peptidyl arginine deiminases (PADIs; HGNC family link) are a family of five enzymes, PADI1‐4 and PADI6. PADIs catalyze the deimination of protein L‐arginine residues to L‐citrulline and ammonia. The human isozymes exhibit tissue‐specific expression patterns [244].

## 36 RAS subfamily

### Overview

The RAS proteins (HRAS, NRAS and KRAS) are small membrane‐localised G protein‐like molecules of 21 kd. They act as an on/off switch linking receptor and non‐receptor tyrosine kinase activation to downstream cytoplasmic or nuclear events. Binding of GTP activates the switch, and hydrolysis of the GTP to GDP inactivates the switch.

The RAS proto‐oncogenes are the most frequently mutated class of proteins in human cancers. Common mutations compromise the GTP‐hydrolysing ability of the proteins causing constitutive activation [457], which leads to increased cell proliferation and decreased apoptosis [550]. Because of their importance in oncogenic transformation these proteins have become the targets of intense drug discovery effort [23].

## 37 4.2.1.1 Carbonate dehydratases

### Overview

Carbonic anhydrases facilitate the interconversion of water and carbon dioxide with bicarbonate ions and protons (EC 4.2.1.1), with over a dozen gene products identified in man. The enzymes function in acid‐base balance and the movement of carbon dioxide and water. They are targetted for therapeutic gain by particular antiglaucoma agents and diuretics.

**Table:** 

Nomenclature	carbonic anhydrase I	carbonic anhydrase VII	carbonic anhydrase XII
HGNC, UniProt	*CA1*, P00915	*CA7*, P43166	*CA12*, O43570
EC number	4.2.1.1	4.2.1.1	4.2.1.1
Inhibitors	chlorthalidone (p*K*_i_ 6.5)	methazolamide (p*K*_i_ 8.7) [430], acetazolamide (p*K*_i_ 8.6) [18], brinzolamide (p*K*_i_ 8.6) [430], chlorthalidone (p*K*_i_ 8.6) [482]	chlorthalidone (p*K*_i_ 8.4) [482], diclofenamide (p*K*_i_ 7.3) [504]

### Further Reading

Alterio V *et al*. (2012) Multiple binding modes of inhibitors to carbonic anhydrases: how to design specific drugs targeting 15 different isoforms? *Chem. Rev.* **112**: 4421‐68 [PMID:22607219]

Cummins EP *et al*. (2014) Carbon dioxide‐sensing in organisms and its implications for human disease. *Cell. Mol. Life Sci.* **71**: 831‐45 [PMID:24045706]

Imtaiyaz Hassan M *et al*. (2013) Structure, function and applications of carbonic anhydrase isozymes. *Bioorg. Med. Chem.* **21**: 1570‐82 [PMID:22607884]

Sjöblom M. (2011) Duodenal epithelial sensing of luminal acid: role of carbonic anhydrases. *Acta Physiol (Oxf)* **201**: 85‐95 [PMID:20632999]

## 38 5.99.1.2 DNA Topoisomerases

### Overview

DNA topoisomerases regulate the supercoiling of nuclear DNA to influence the capacity for replication or transcription. The enzymatic function of this series of enzymes involves cutting the DNA to allow unwinding, followed by re‐attachment to reseal the backbone. Members of the family are targetted in anti‐cancer chemotherapy.

**Table:** 

Nomenclature	topoisomerase (DNA) I	topoisomerase (DNA) II alpha 170kDa
HGNC, UniProt	*TOP1*, P11387	*TOP2A*, P11388
EC number	5.99.1.2	5.99.1.2
Inhibitors	irinotecan [117, 477] – Bovine	etoposide (pIC_50_ 7.3), teniposide [119] – Mouse

### Further Reading

Castelli S *et al*. (2012) Interaction between natural compounds and human topoisomerase I. *Biol. Chem.* **393**: 1327‐40 [PMID:23109546]

Chen SH *et al*. (2013) New mechanistic and functional insights into DNA topoisomerases. *Annu. Rev. Biochem.* **82**: 139‐70 [PMID:23495937]

Kathiravan MK *et al*. (2013) Topoisomerase as target for antibacterial and anticancer drug discovery. *J Enzyme Inhib Med Chem* **28**: 419‐35 [PMID:22380774]

Tomicic MT *et al*. (2013) Topoisomerase degradation, DSB repair, p53 and IAPs in cancer cell resistance to camptothecin‐like topoisomerase I inhibitors. *Biochim. Biophys. Acta* **1835**: 11‐27 [PMID:23006513]
